# Supplementary material for: Fission yeast RPA–TERT–Tpz1TPP1 complex promotes telomere extension and suppresses telomere recombination
Source: PLoS Genet. 2026 Sep 8;22(9):e1012297. doi: 10.1371/journal.pgen.1012297 (PMC13577582; doi:10.1371/journal.pgen.1012297)

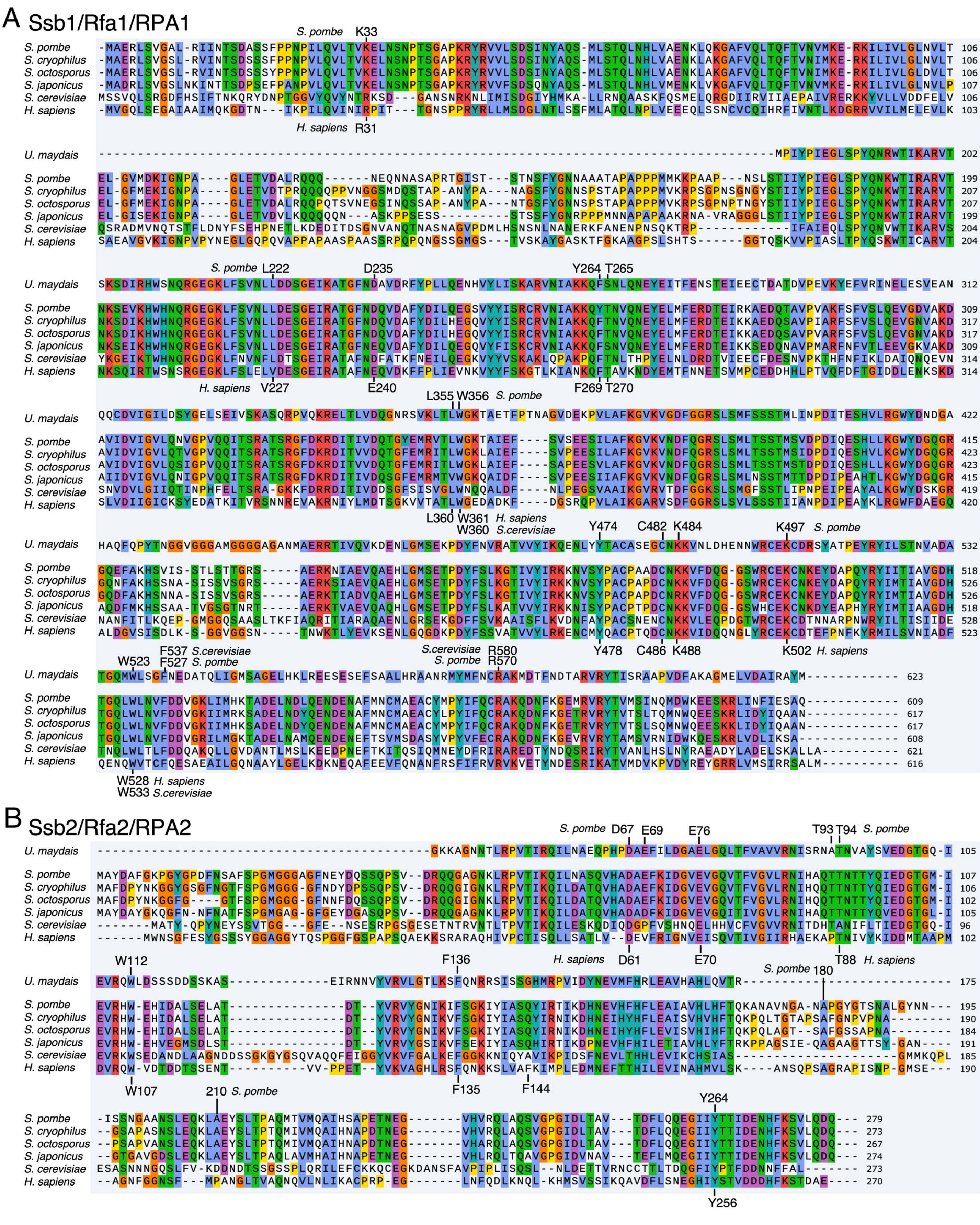
A Ssb1/Rfa1/RPA1
S. pombe
S. cryophilus
S. octosporus
S. japonicus
S. cerevisiae
H. sapiens
U. maydais
S. pombe K33
H. sapiens R31
S. pombe L222
D235
Y264 T265
H. sapiens V227
E240
F269 T270
L355 W356 S. pombe
L360 W361 H. sapiens
W360 S.cerevisiae
Y474
C482 K484
K497 S. pombe
Y478
C486 K488
K502 H. sapiens
W523
F537 S.cerevisiae
F527 S. pombe
S.cerevisiae R580
S. pombe R570
W528 H. sapiens
W533 S.cerevisiae
B Ssb2/Rfa2/RPA2
S. pombe D67
E69
E76
T93 T94 S. pombe
H. sapiens D61
E70
T88 H. sapiens
W112
F136
S. pombe 180
W107
F135
F144
210 S. pombe
Y264
Y256

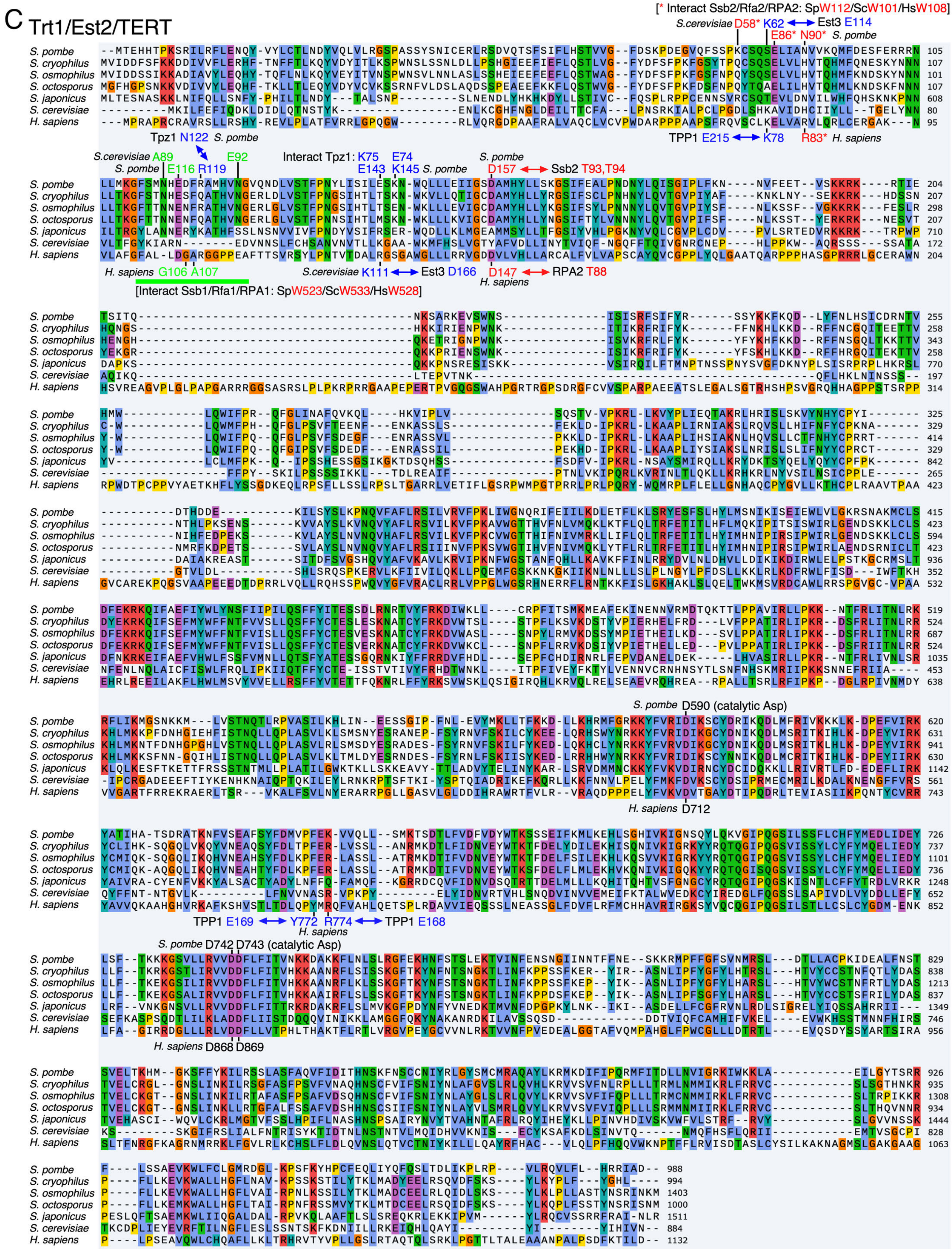
C Trt1/Est2/TERT
[* Interact Ssb2/Rfa2/RPA2: SpW112/ScW101/HsW108]
S.cerevisiae D58* K62 Est3 E114
E86* N90* S. pombe
S. pombe
S. cryophilus
S. osmophilus
S. octosporus
S. japonicus
S. cerevisiae
H. sapiens
Tpz1 N122 S. pombe
TPP1 E215 K78
R83* H. sapiens
S.cerevisiae A89
S. pombe E116 R119
E92
Interact Tpz1: K75 E74
E143 K145 S. pombe
S. pombe
D157 Ssb2 T93,T94
H. sapiens G106 A107
[Interact Ssb1/Rfa1/RPA1: SpW523/ScW533/HsW528]
S.cerevisiae K111 Est3 D166
D147 RPA2 T88
H. sapiens
S. pombe D590 (catalytic Asp)
H. sapiens D712
TPP1 E169 Y772 R774 TPP1 E168
H. sapiens
S. pombe D742 D743 (catalytic Asp)
H. sapiens D868 D869

D

Tpz1/Est3/TPP1 N-terminal domain (Adjusted based on AlphaFold3 structure)

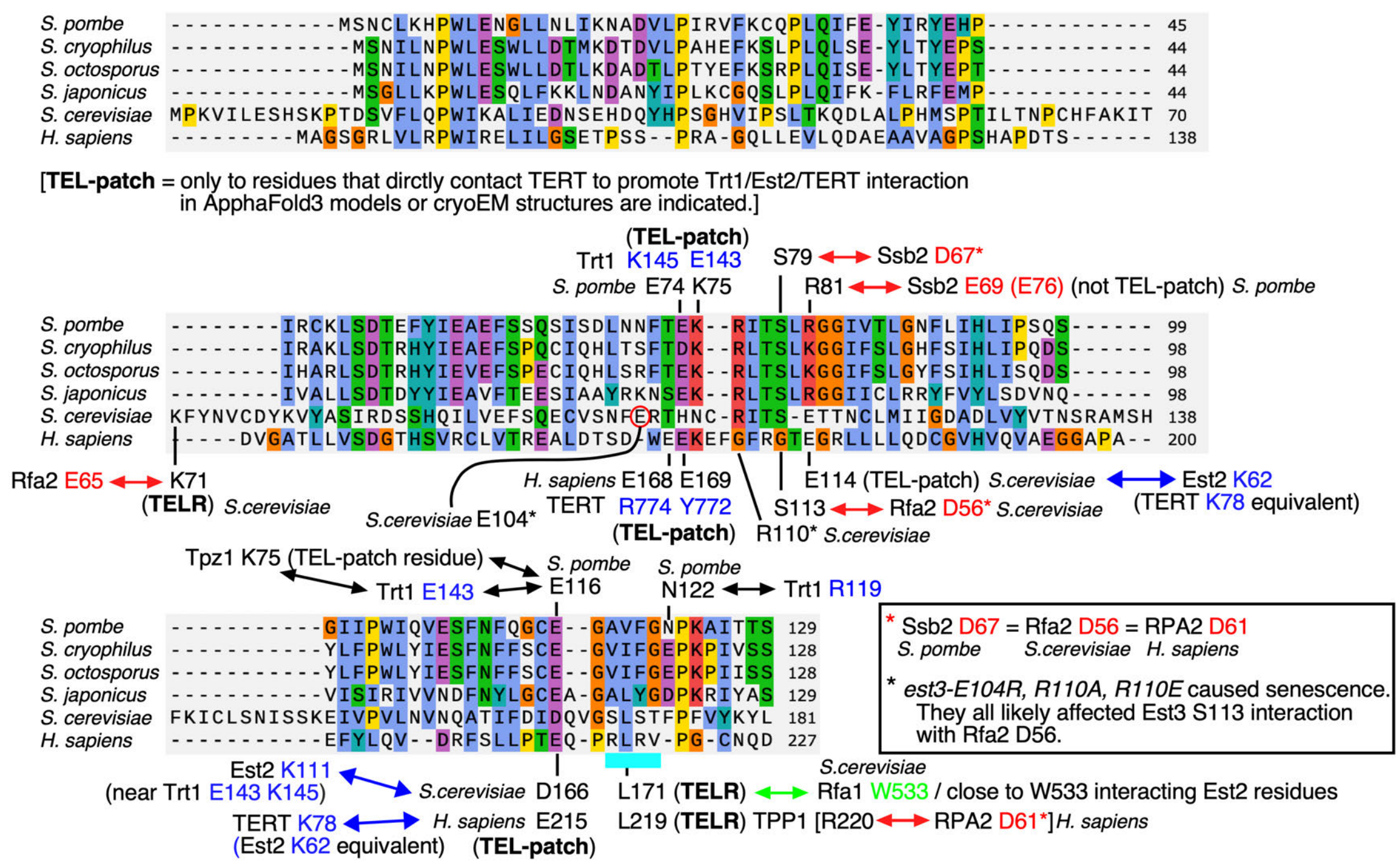

Supplement: Supplementary file 1 [file pgen.1012297.s003.pdf]
