# Supplementary material for: Fission yeast RPA–TERT–Tpz1TPP1 complex promotes telomere extension and suppresses telomere recombination
Source: PLoS Genet. 2026 Sep 8;22(9):e1012297. doi: 10.1371/journal.pgen.1012297 (PMC13577582; doi:10.1371/journal.pgen.1012297)

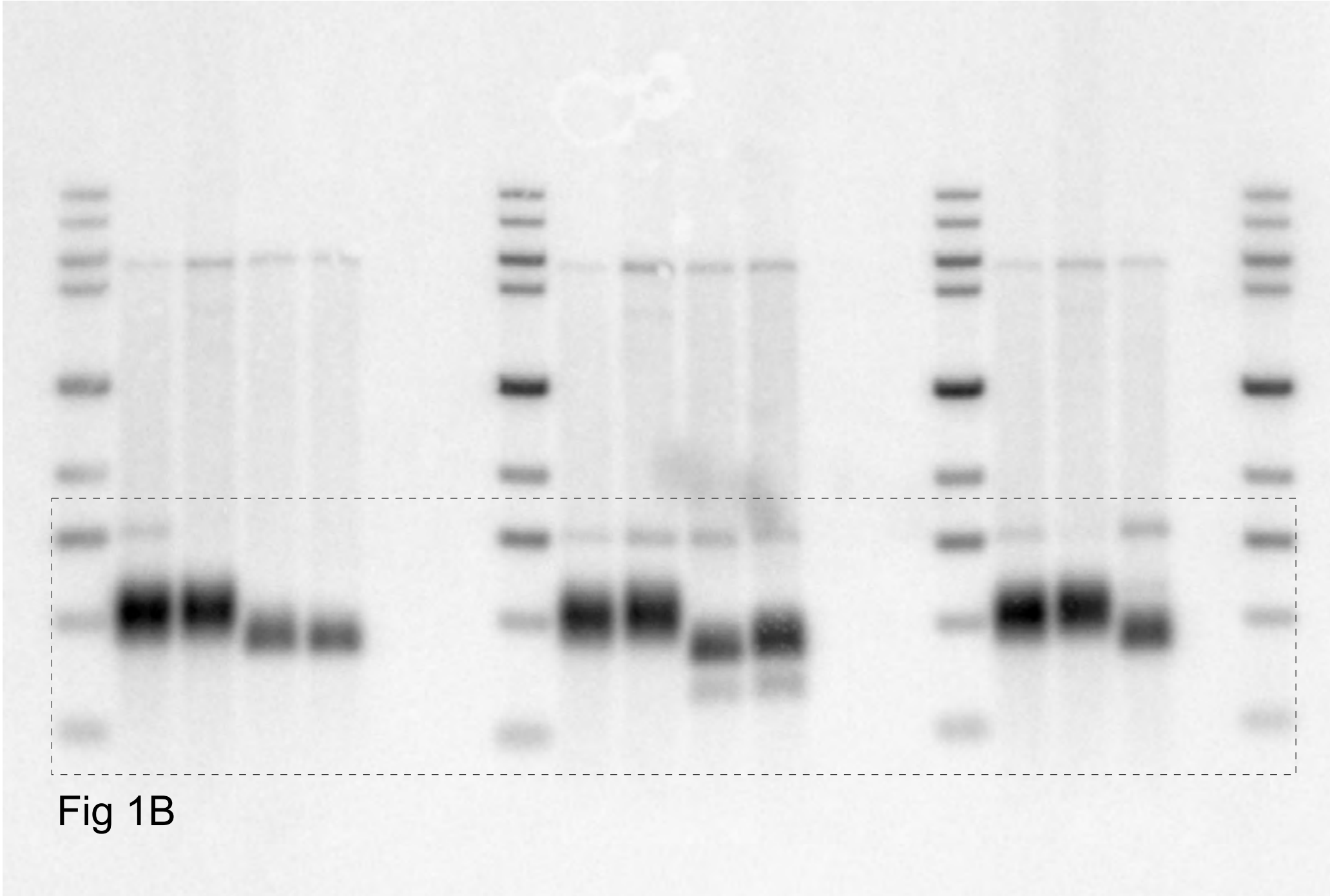
Fig 1B

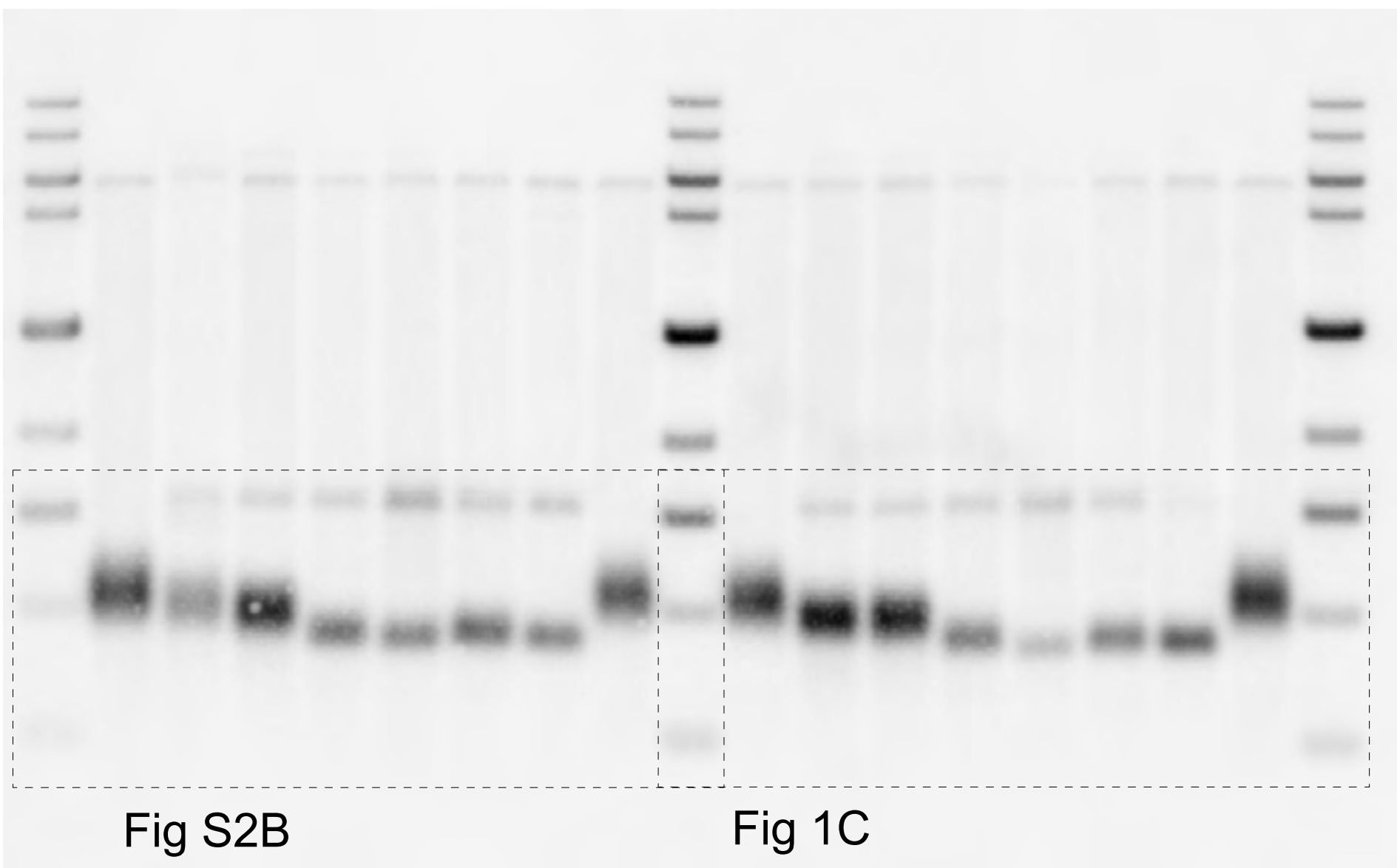
Fig S2B
Fig 1C

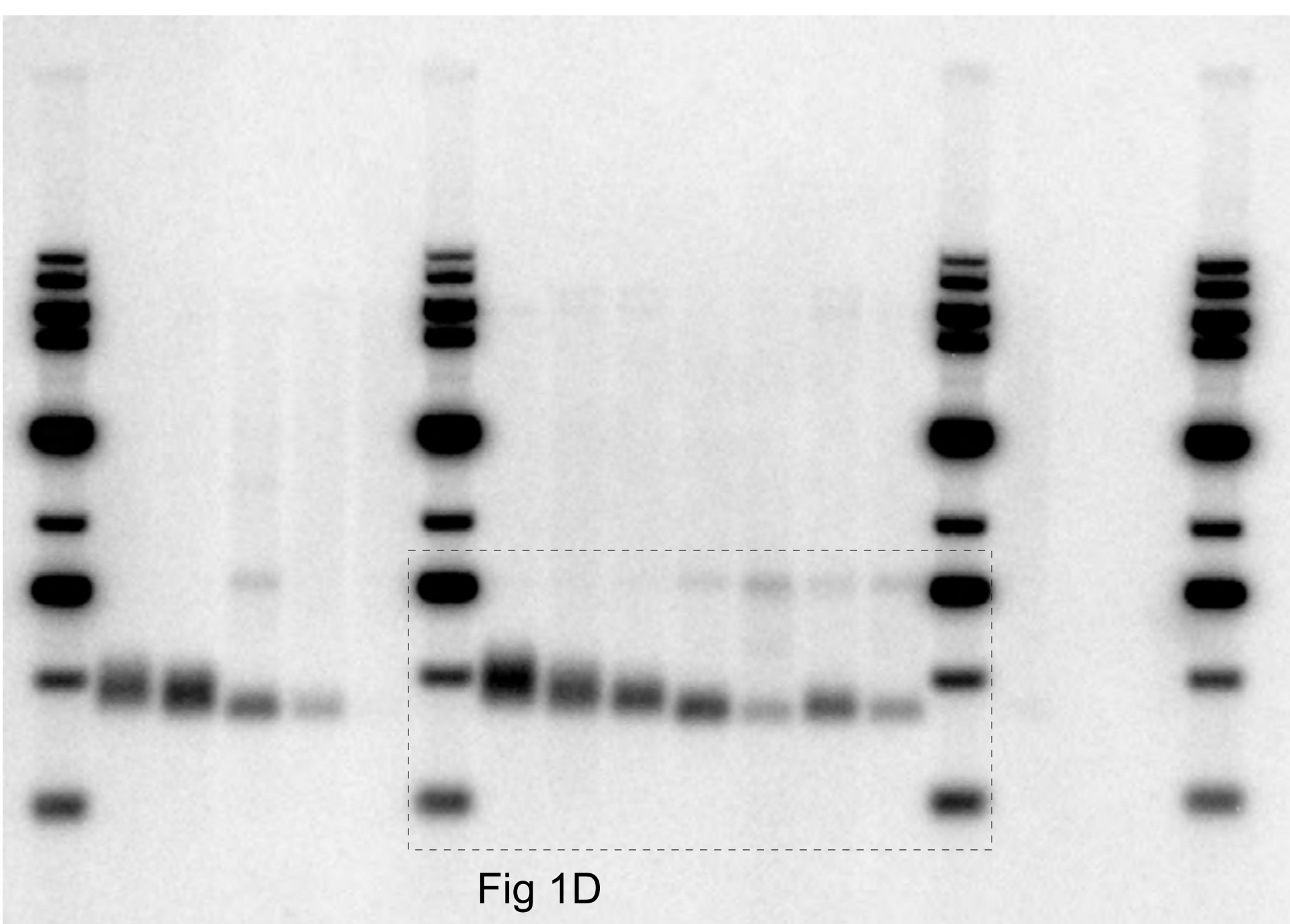
Fig 1D

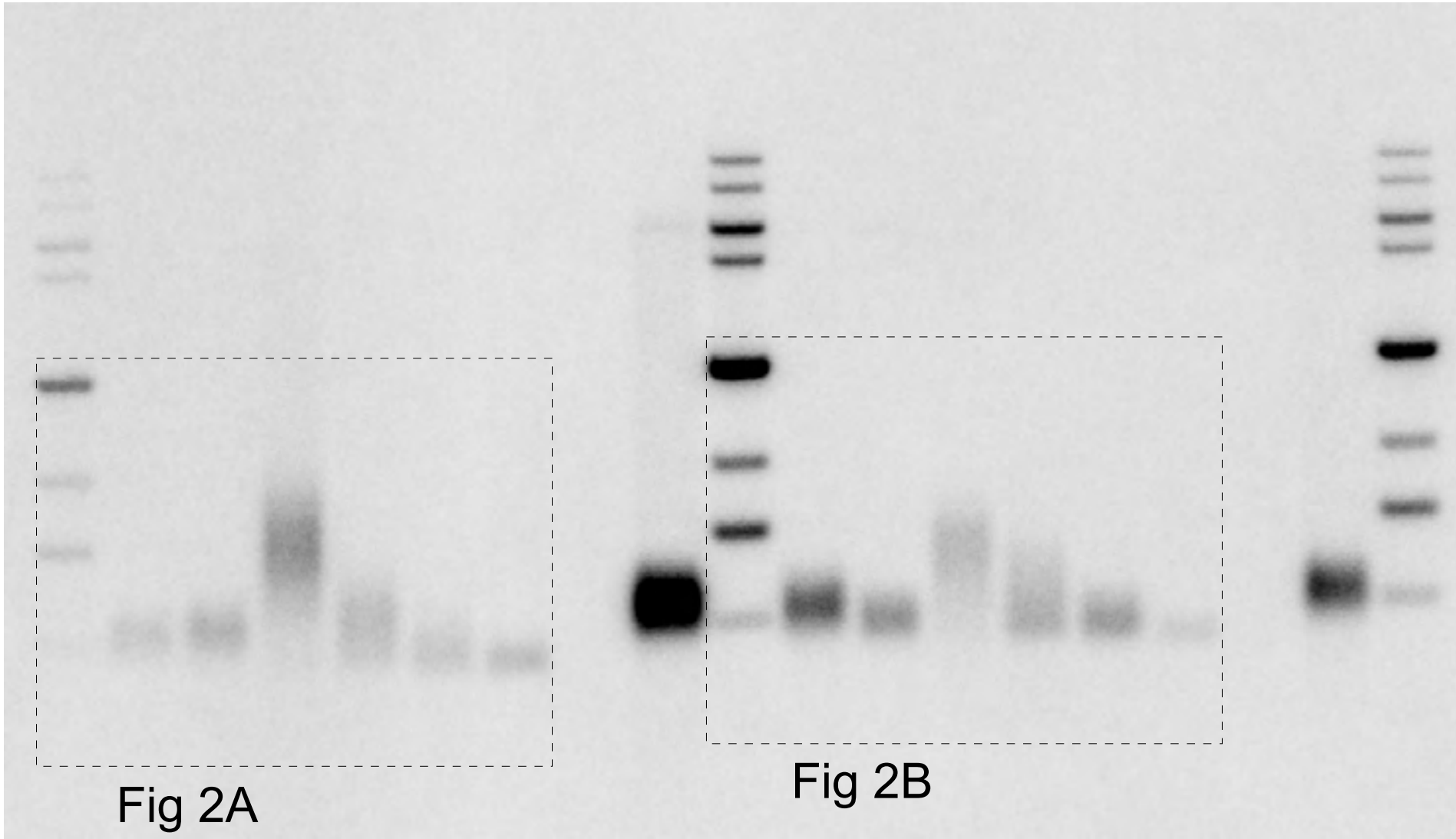

Fig 2A Fig 2B

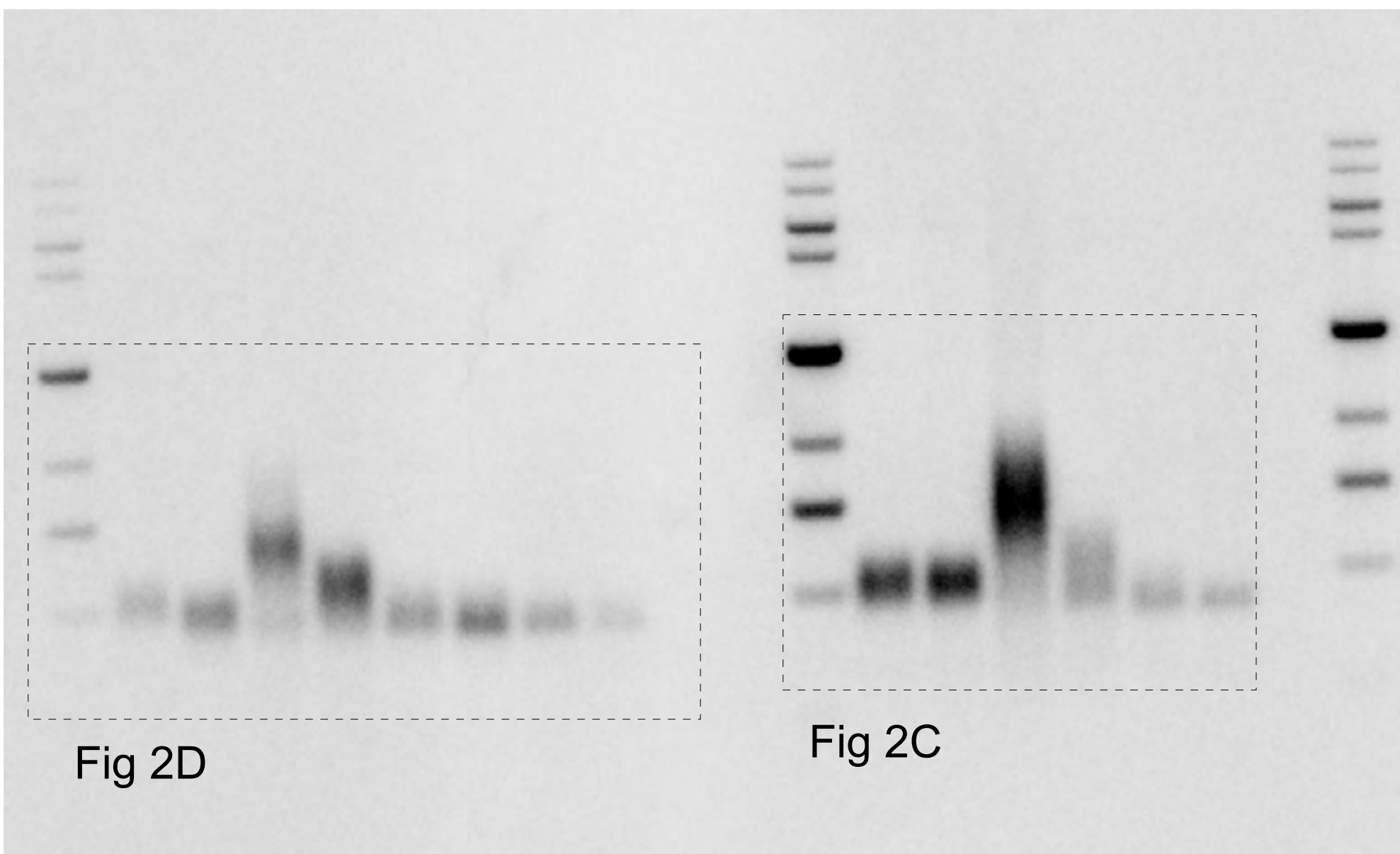

Fig 2D Fig 2C

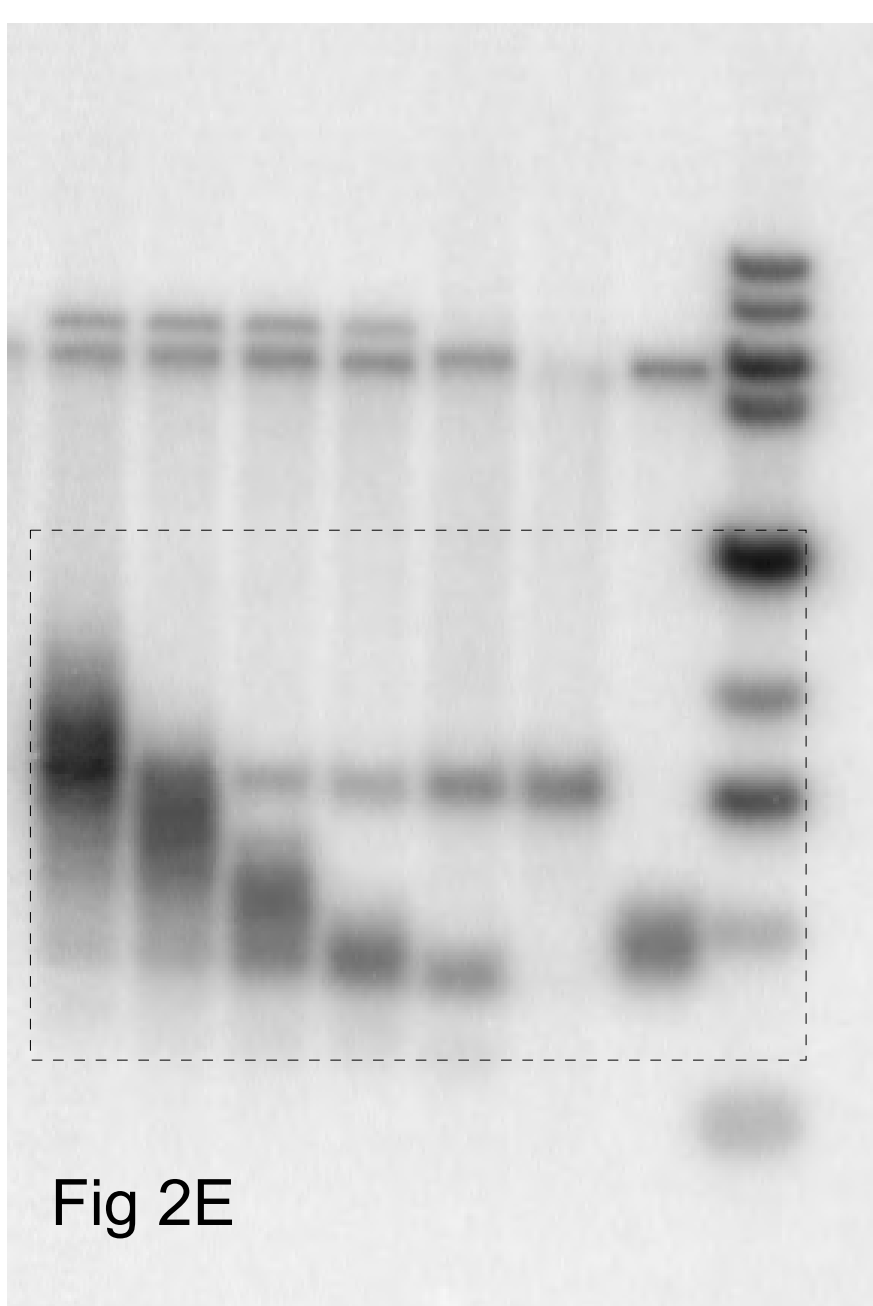

Fig 2E

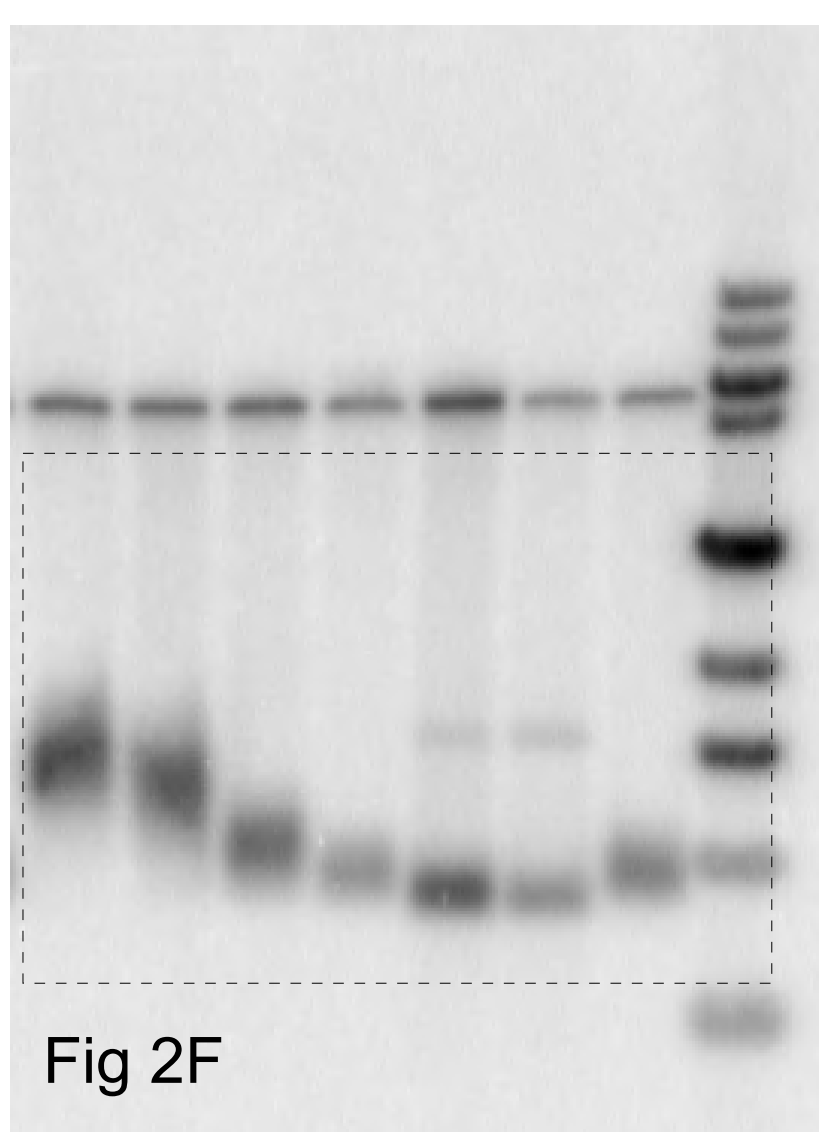

Fig 2F

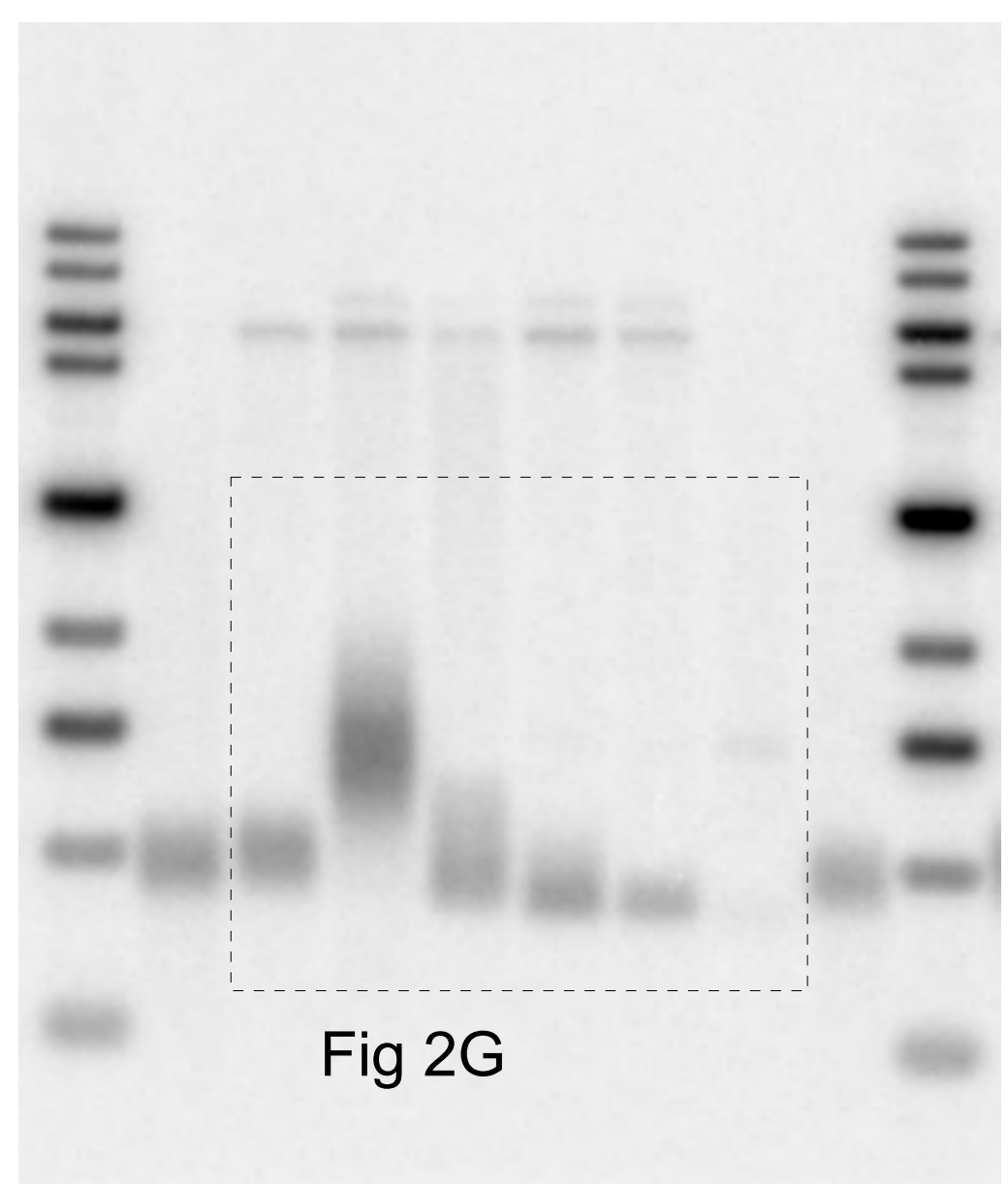

Fig 2G

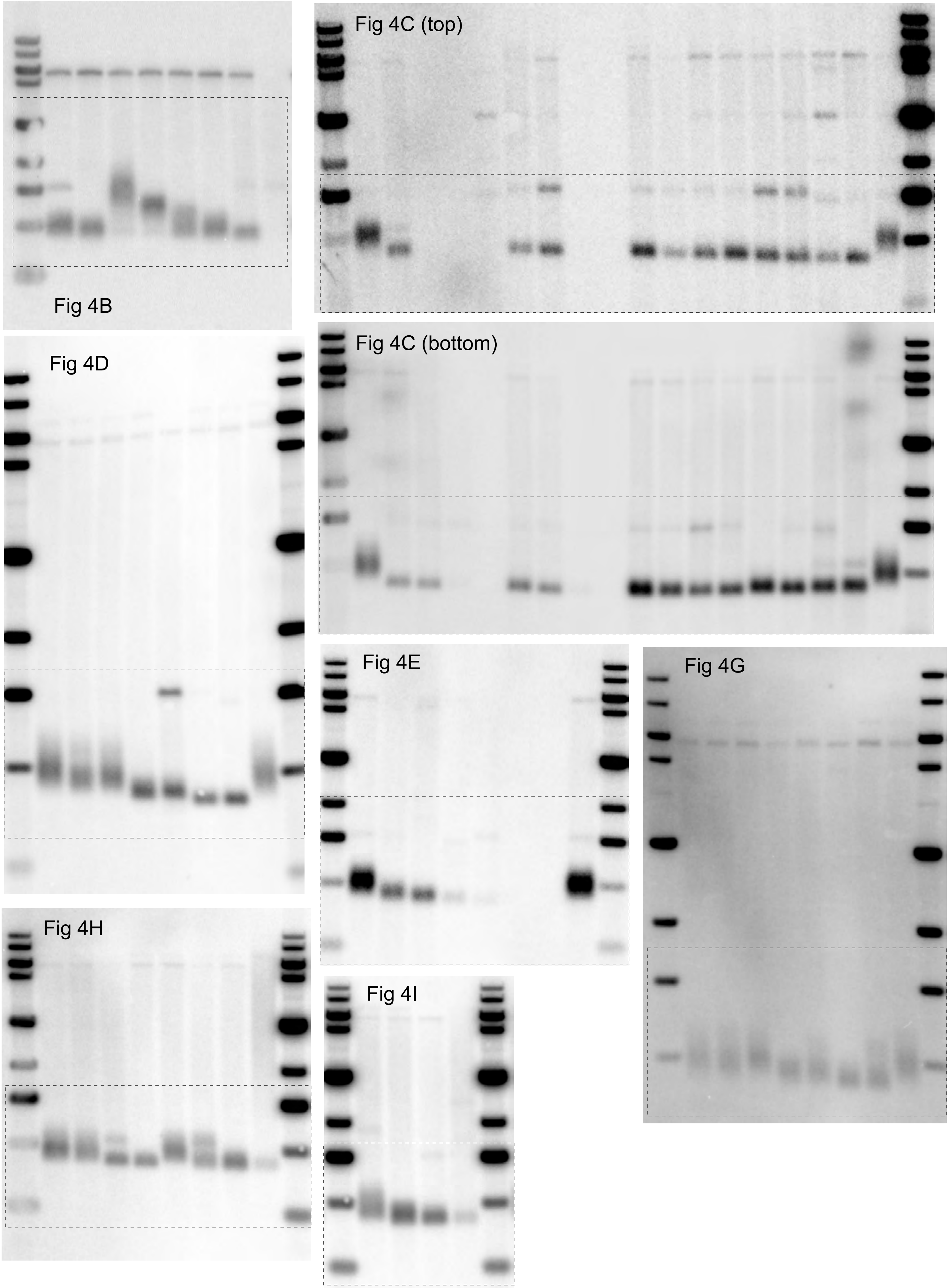
Fig 4B
Fig 4C (top)
Fig 4C (bottom)
Fig 4D
Fig 4E
Fig 4G
Fig 4H
Fig 4I

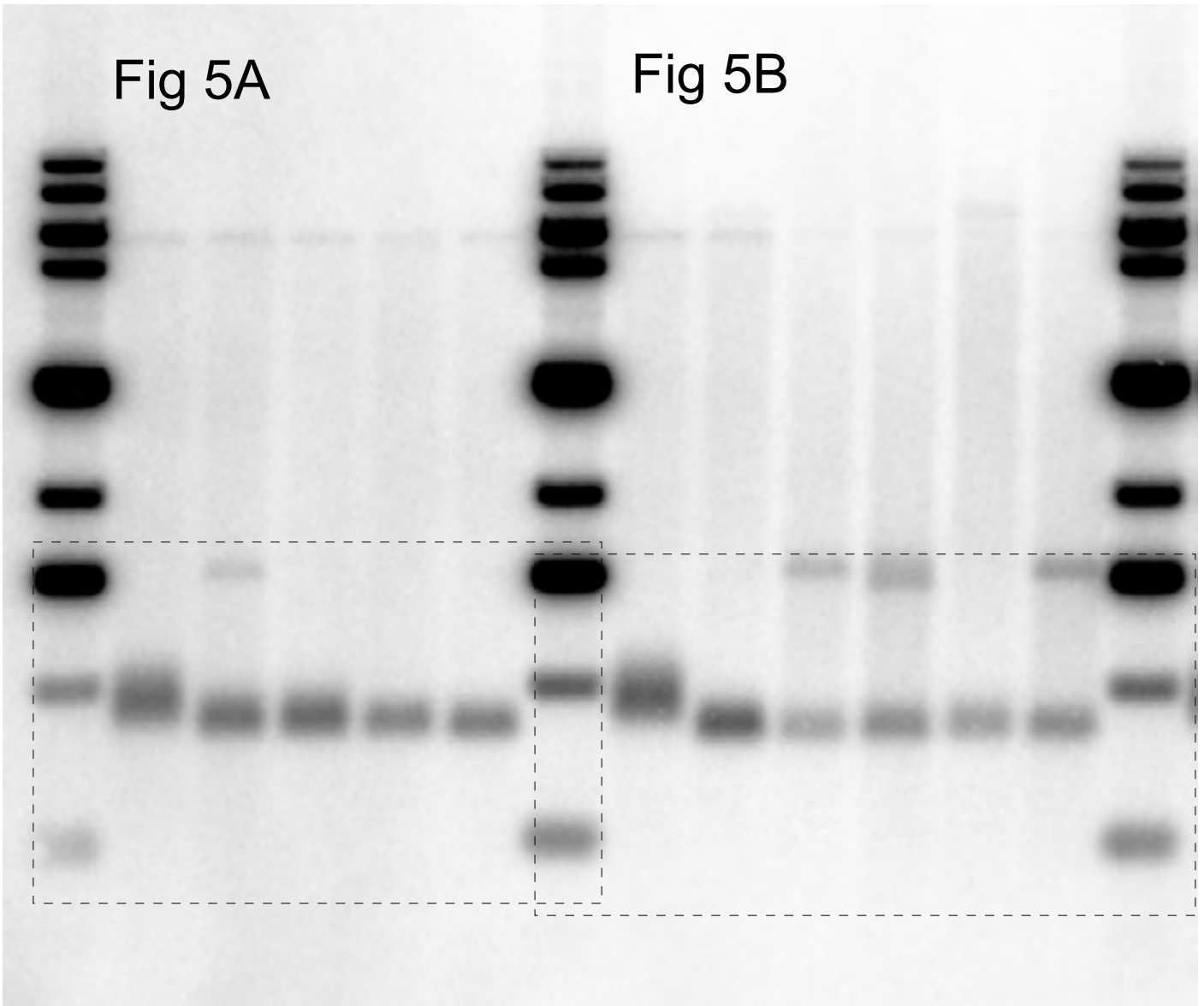
Fig 5A
Fig 5B
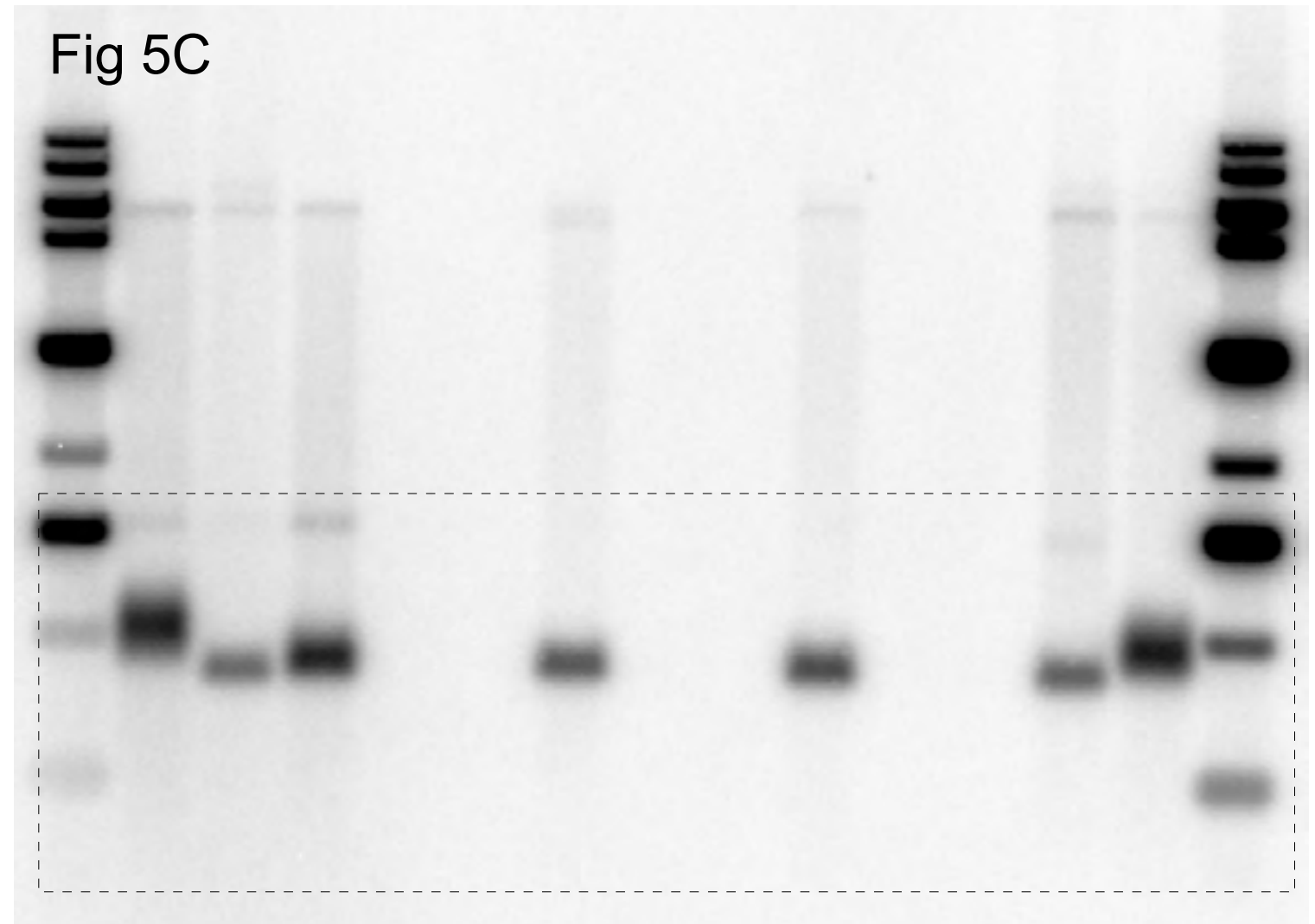
Fig 5C
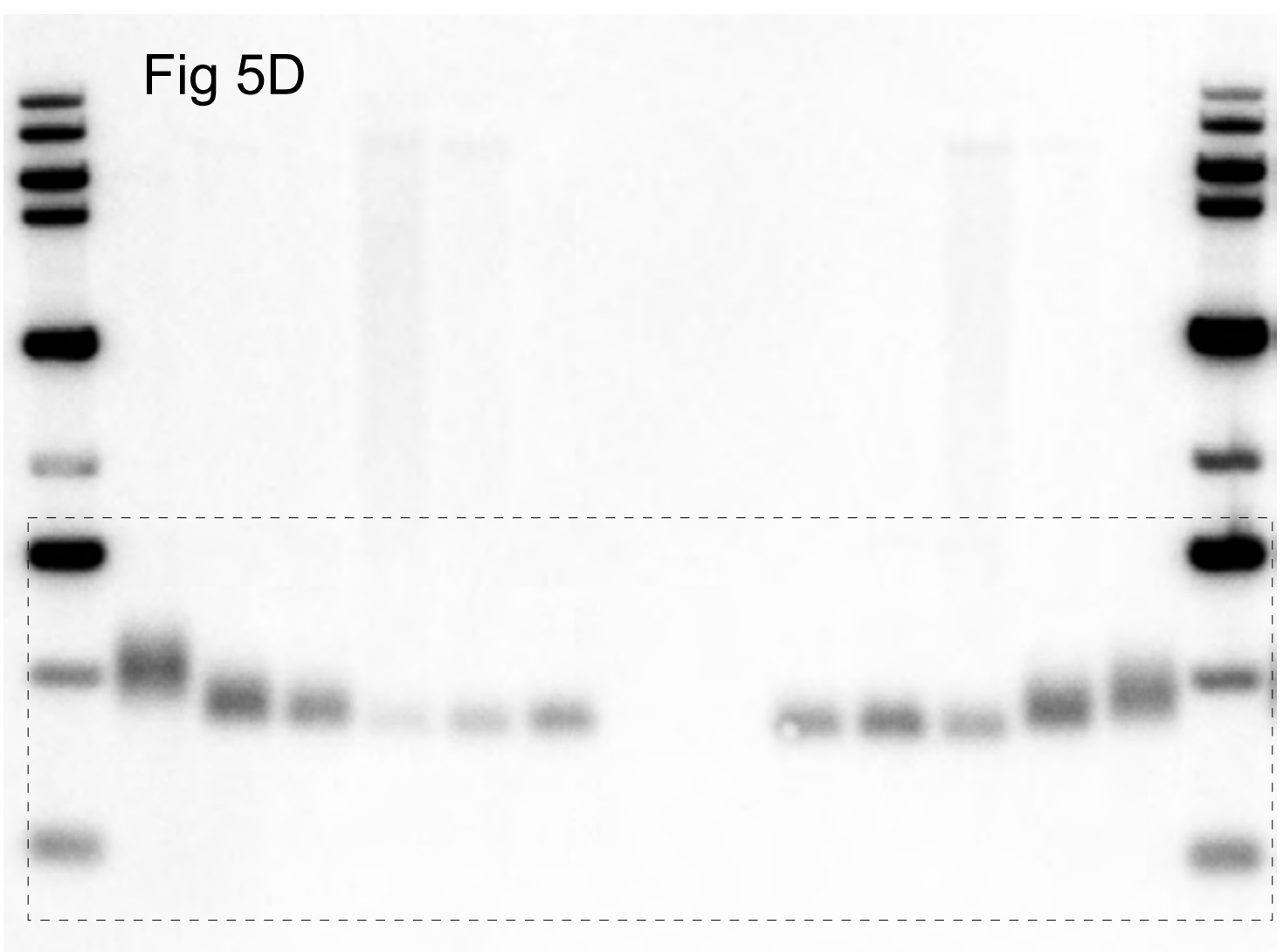
Fig 5D
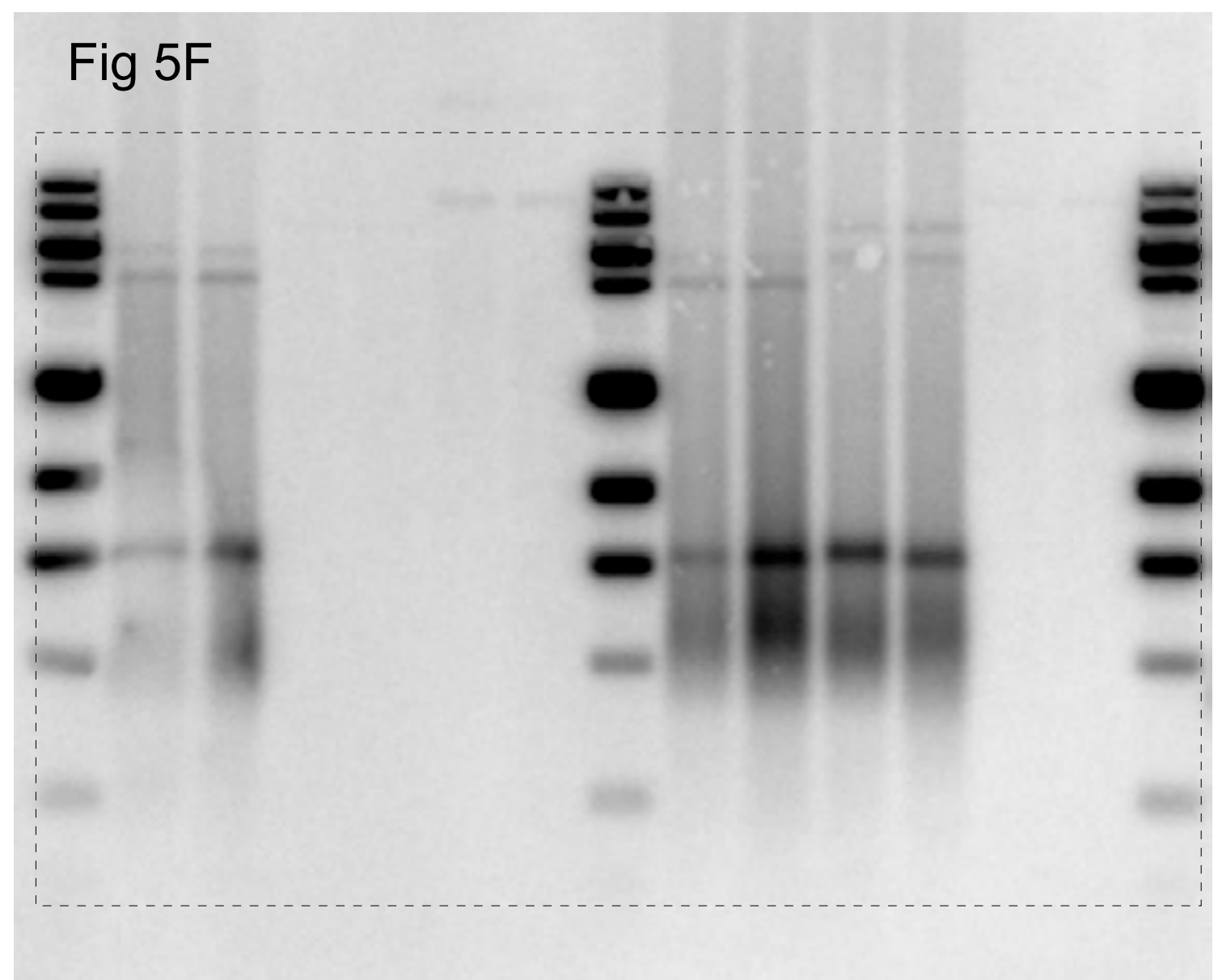
Fig 5F
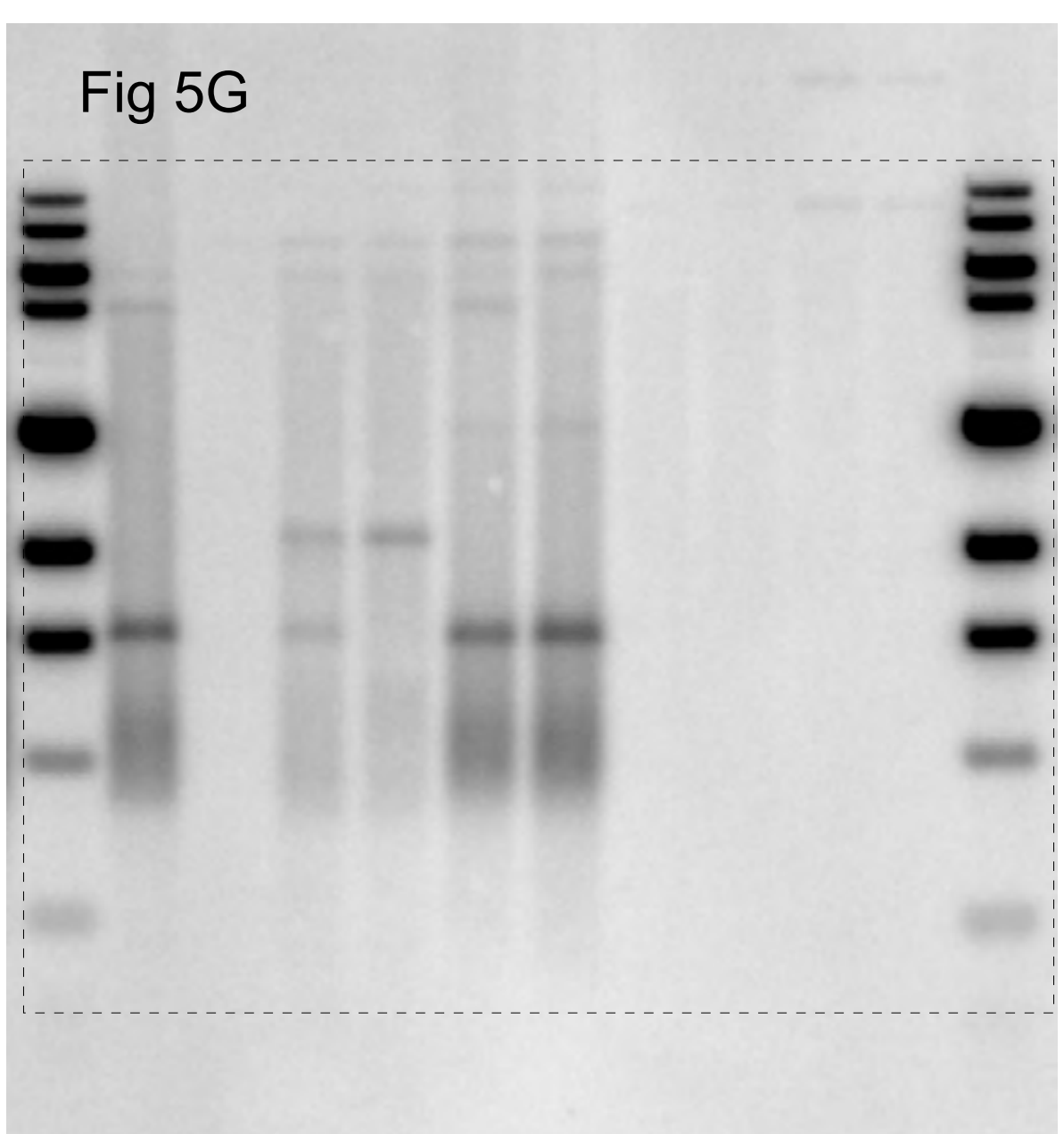
Fig 5G
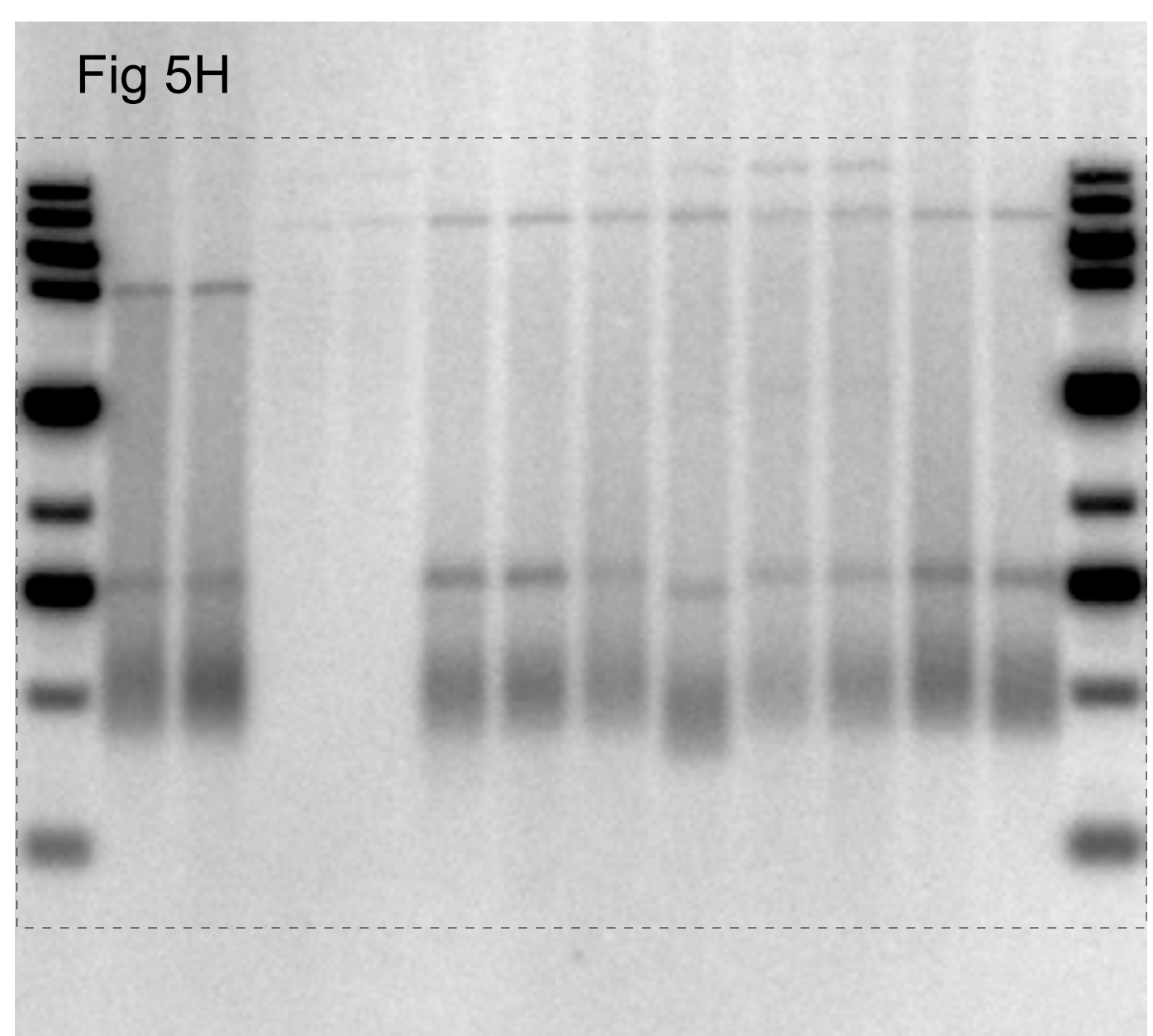
Fig 5H

Fig 6B

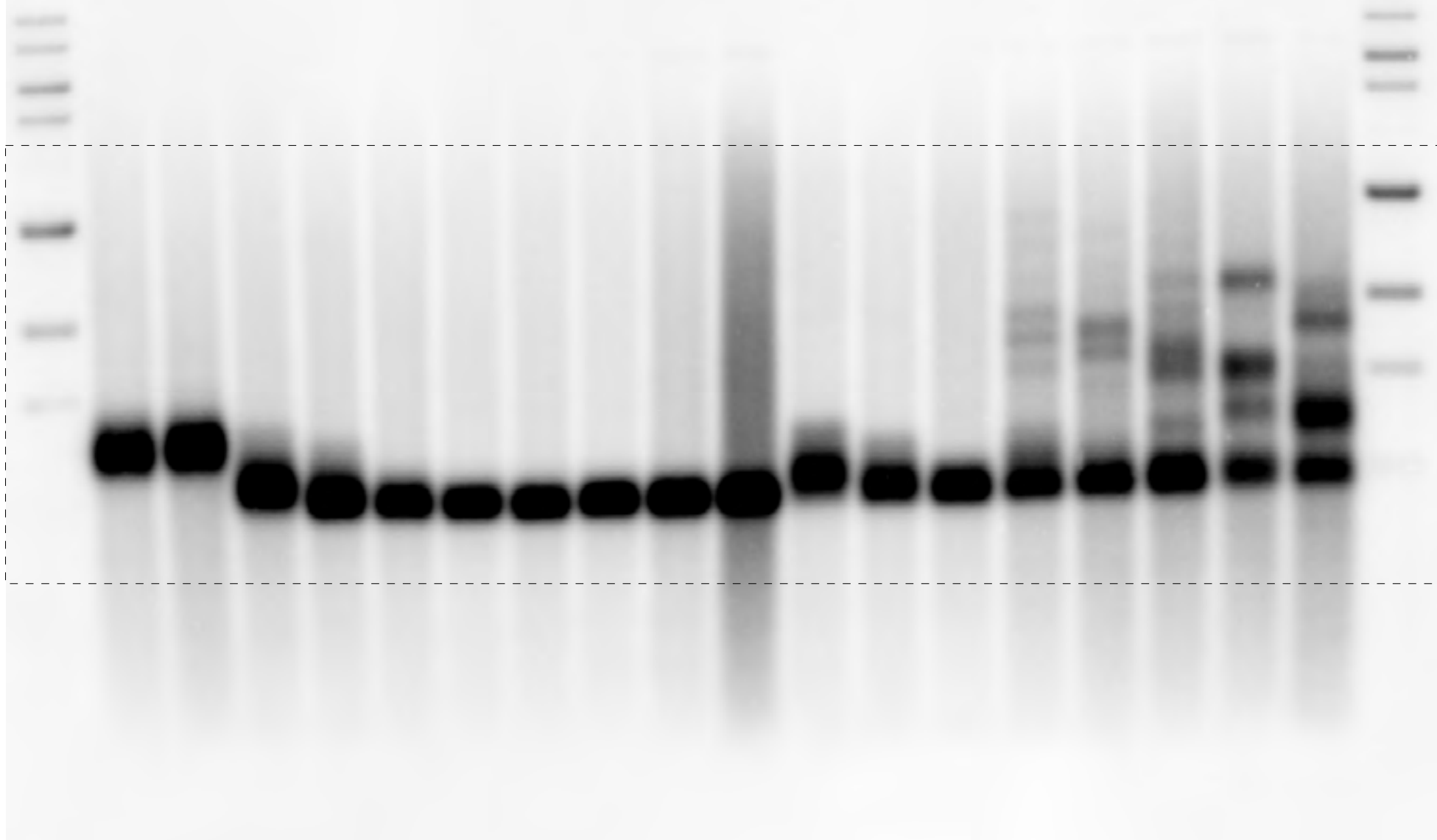

Fig 6C

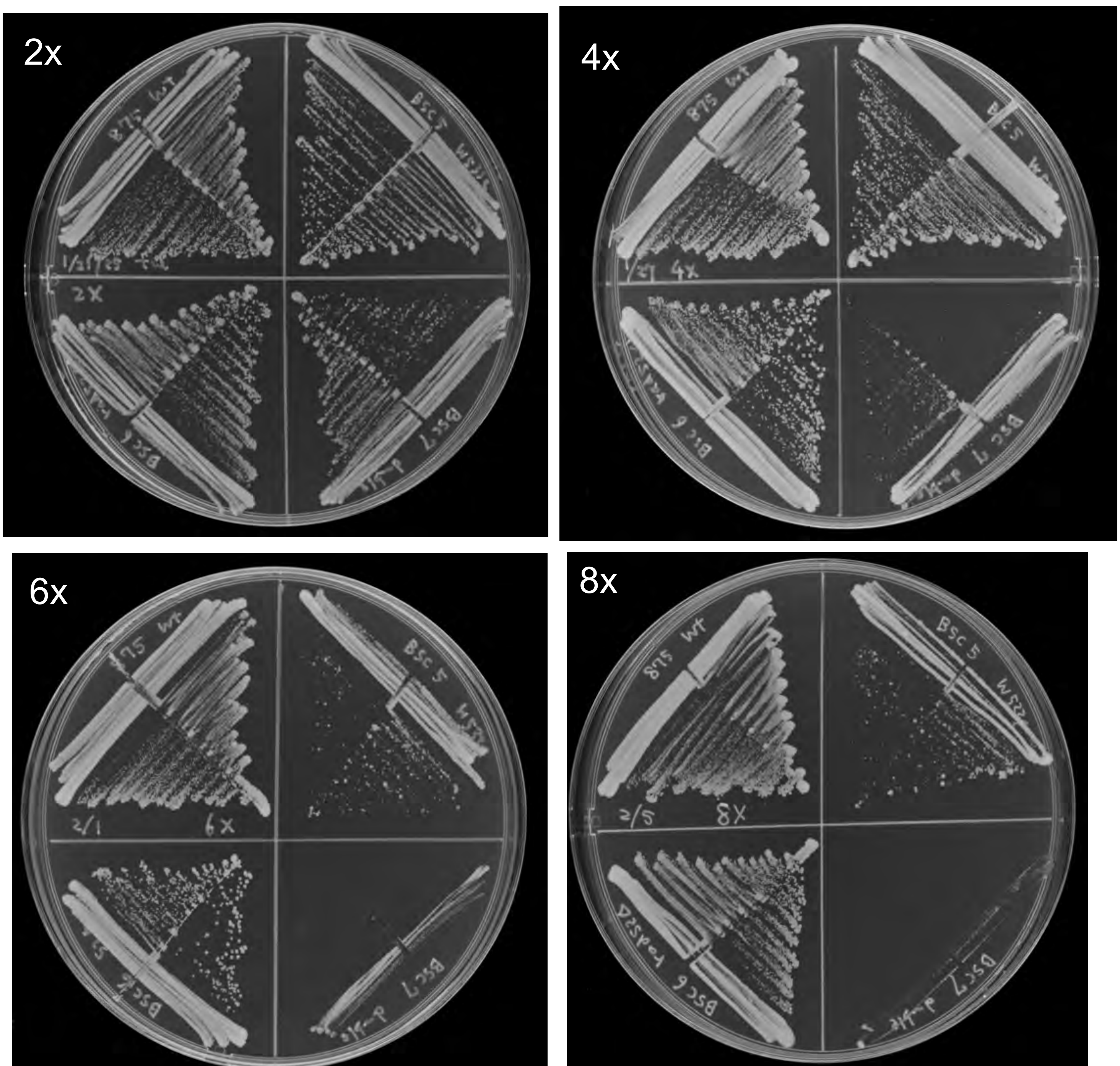

Fig 6E

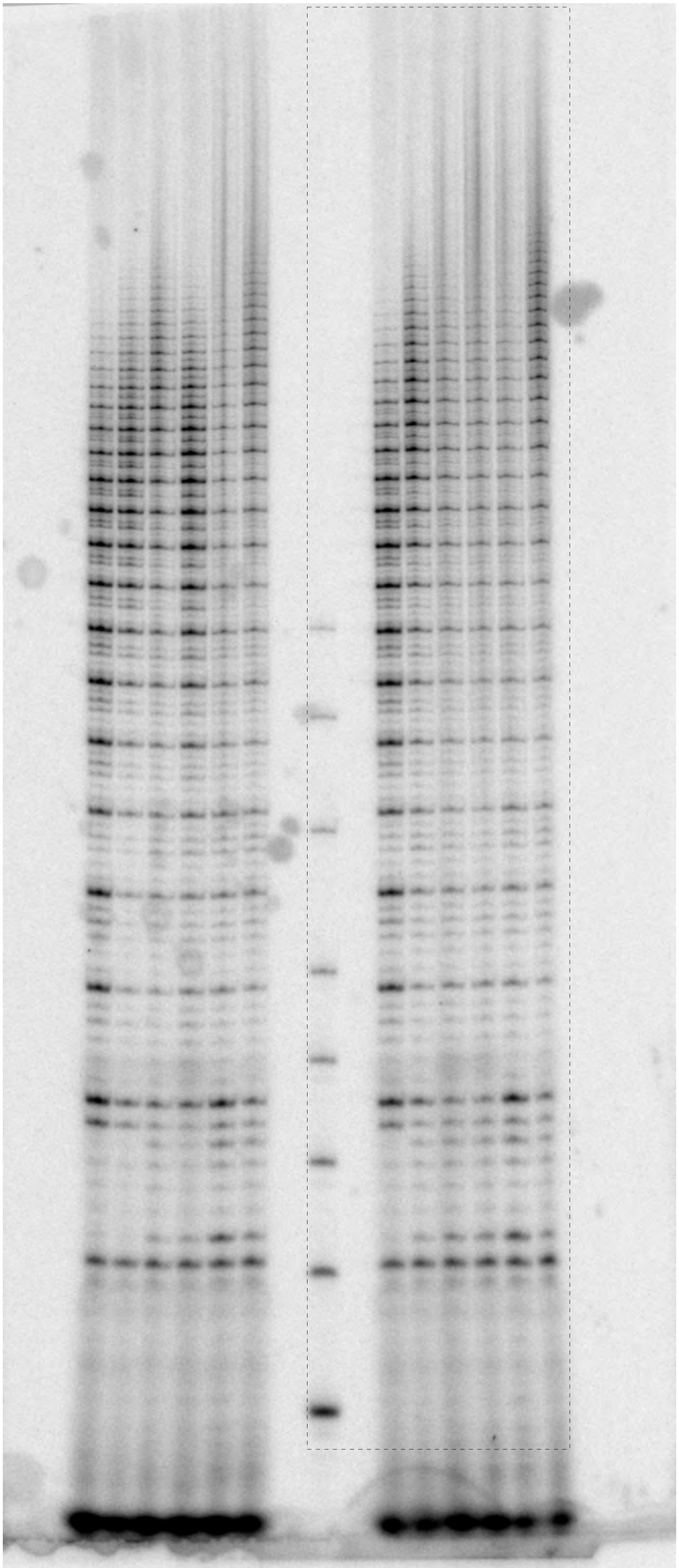

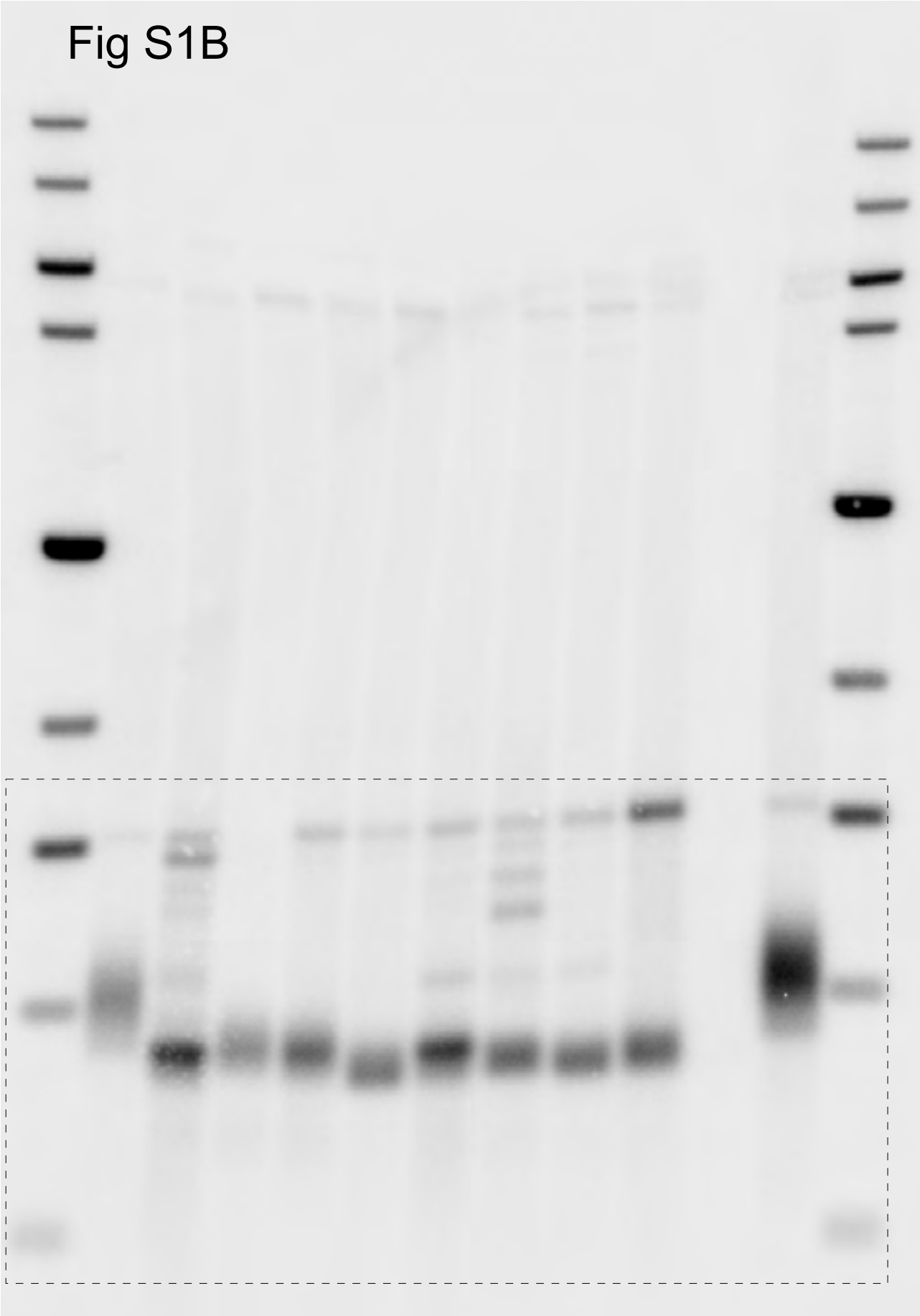
Fig S1B

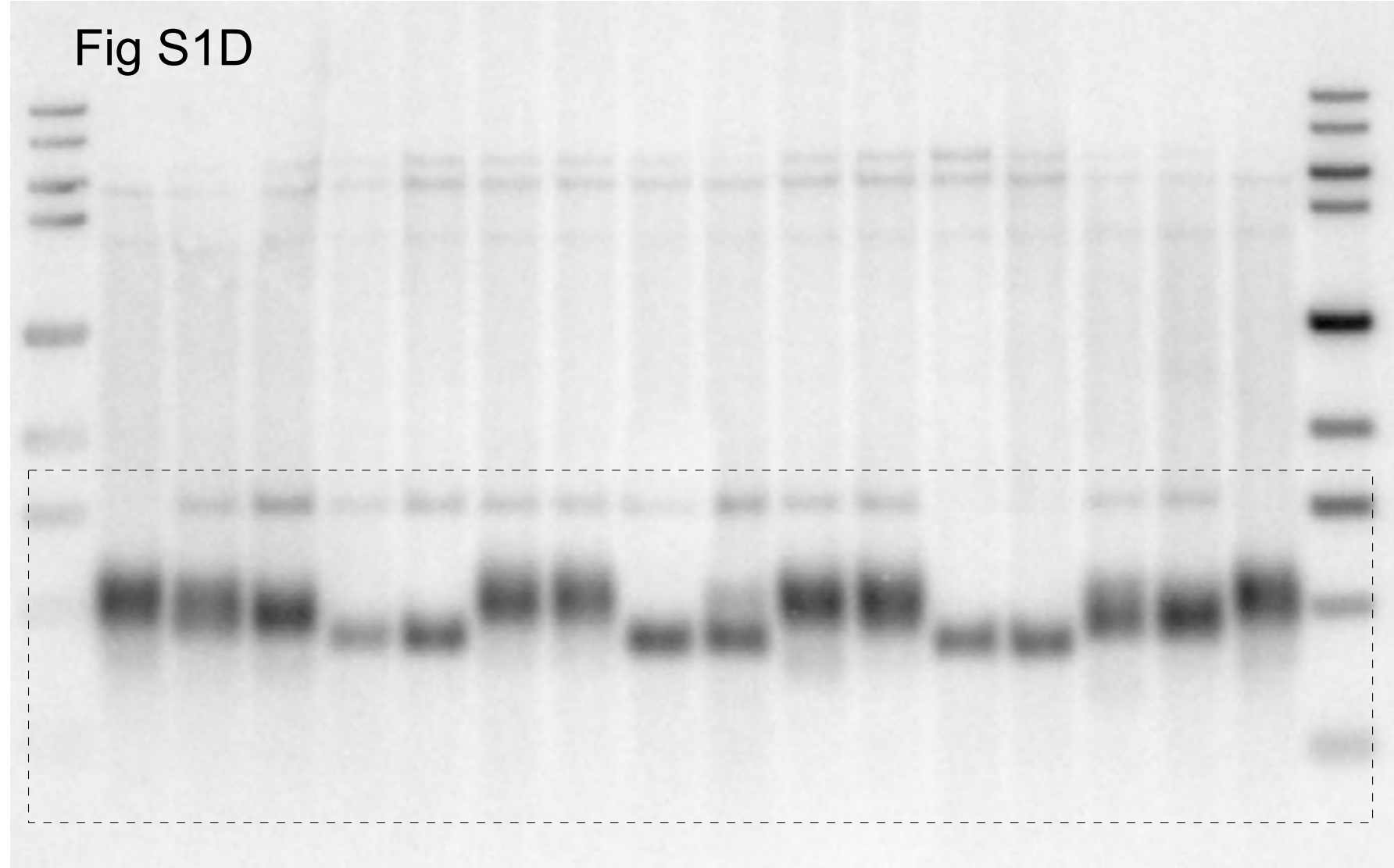
Fig S1D

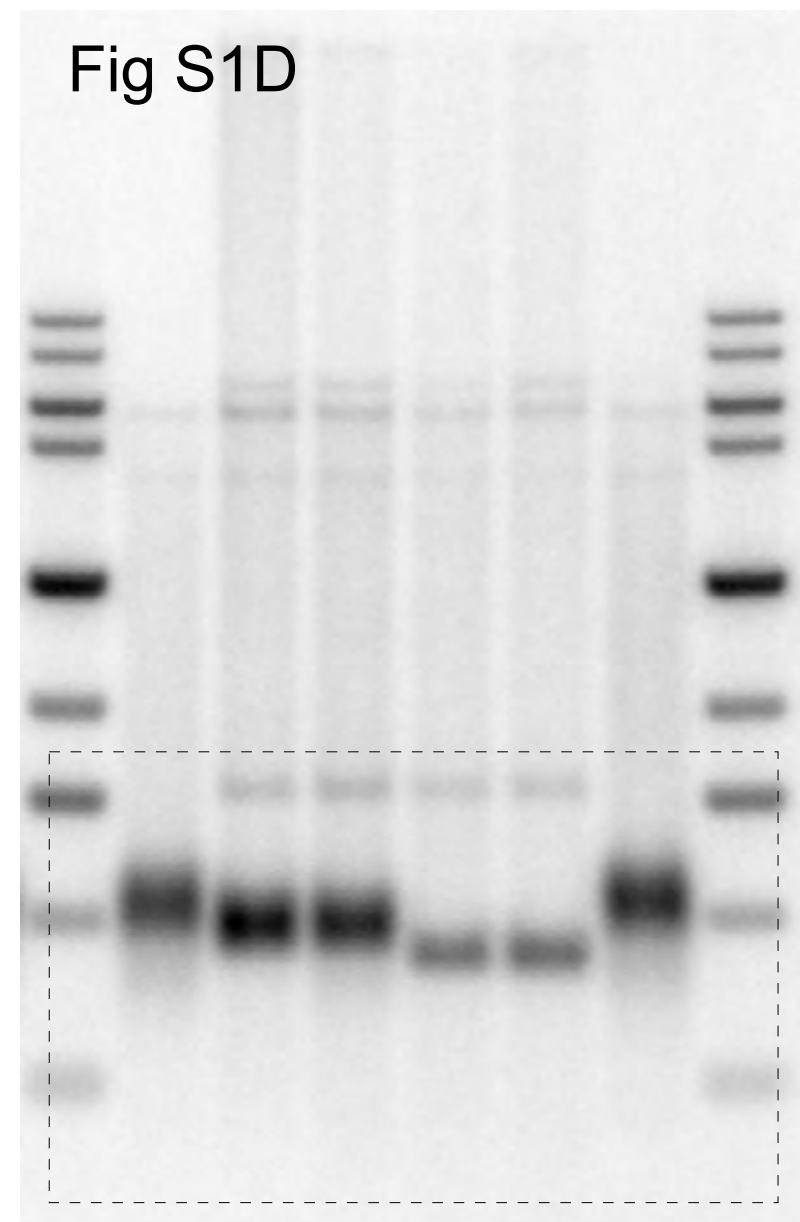
Fig S1D

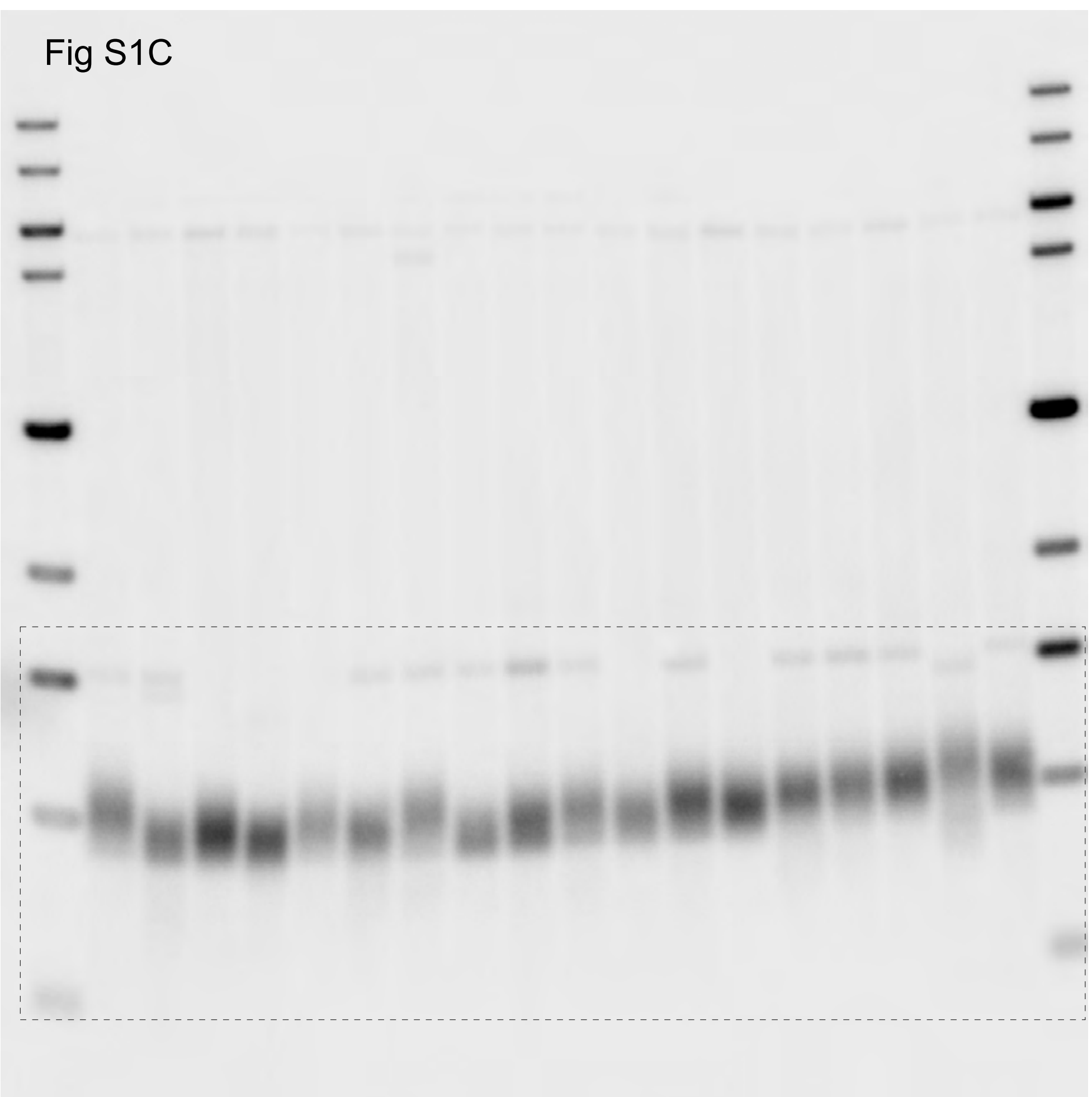
Fig S1C

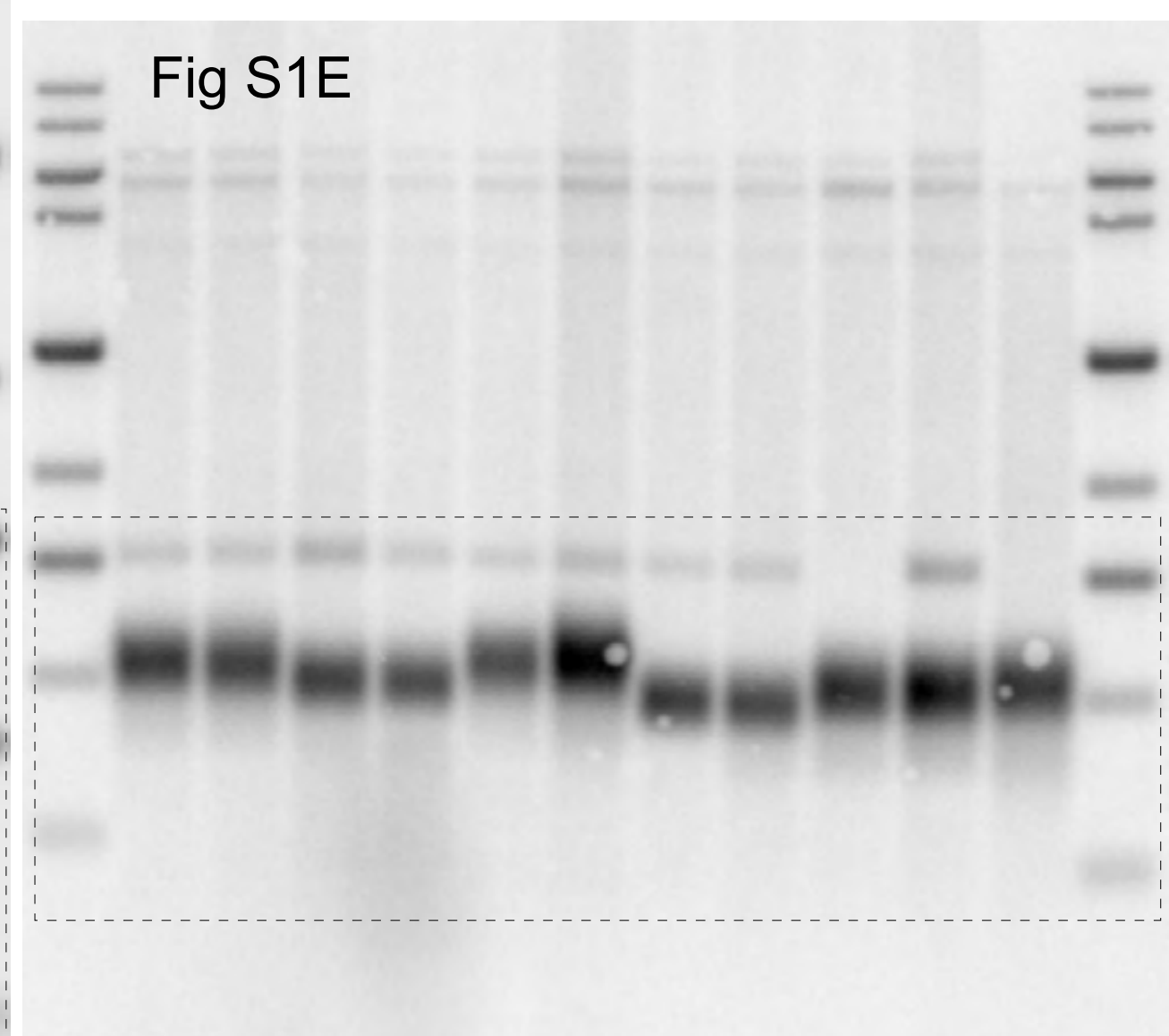
Fig S1E

Fig S2D

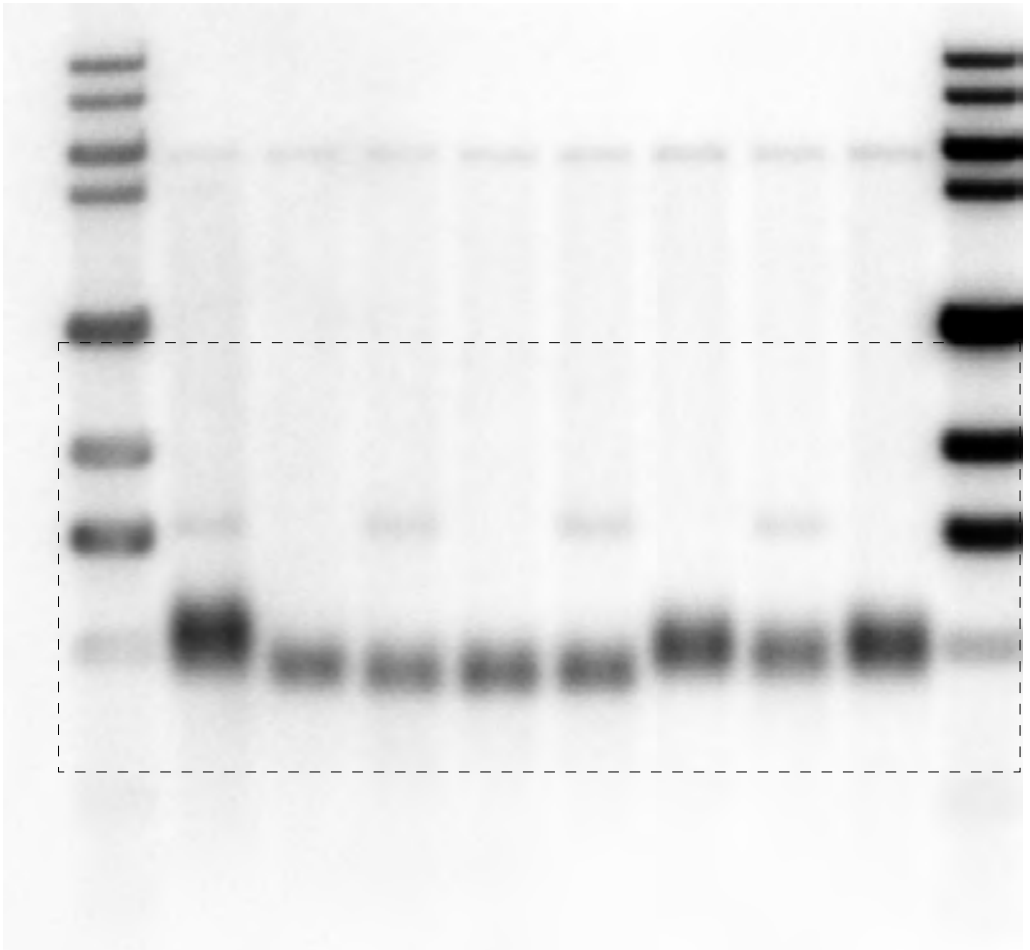

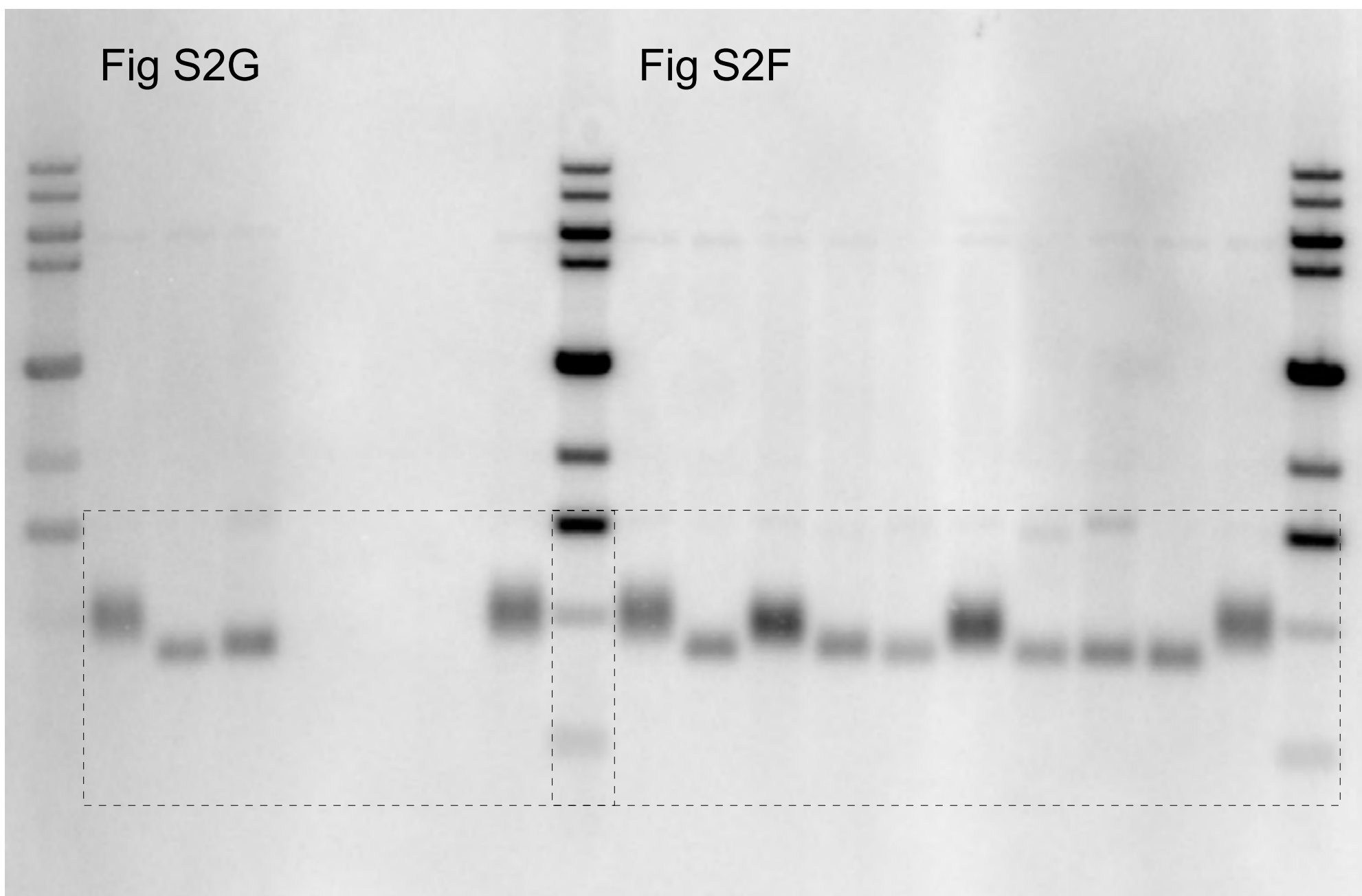

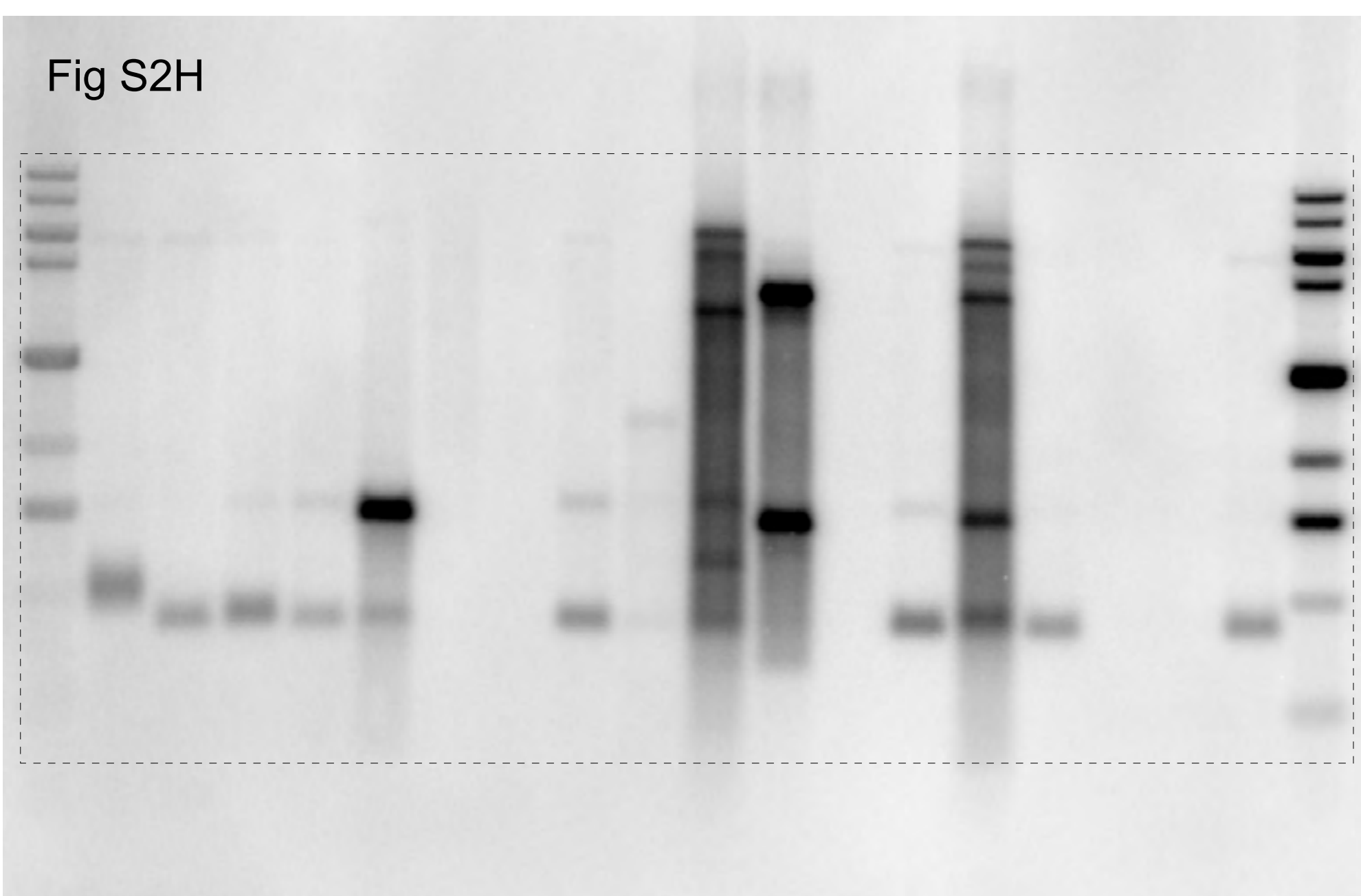

Fig S2C

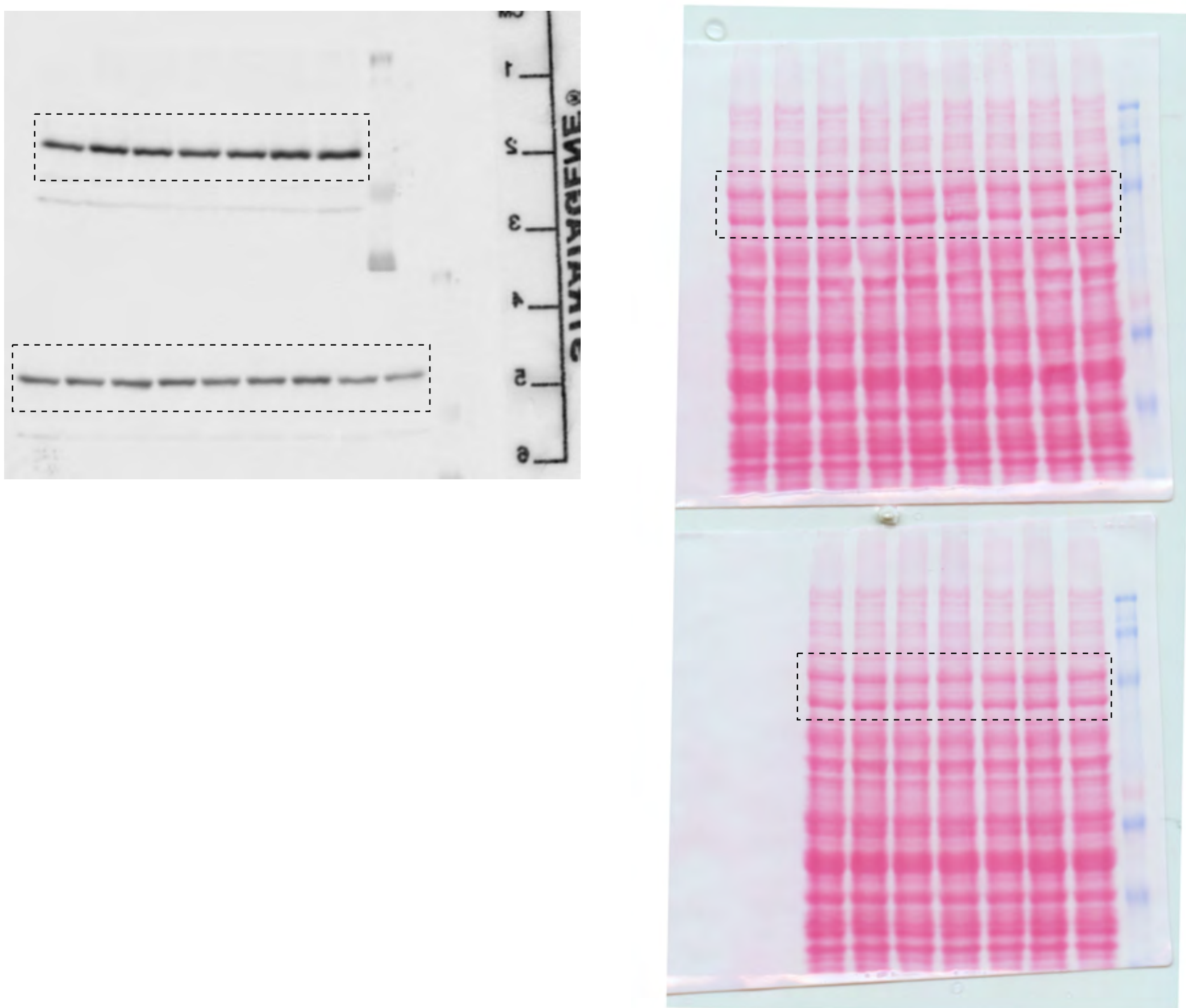

Fig S4A

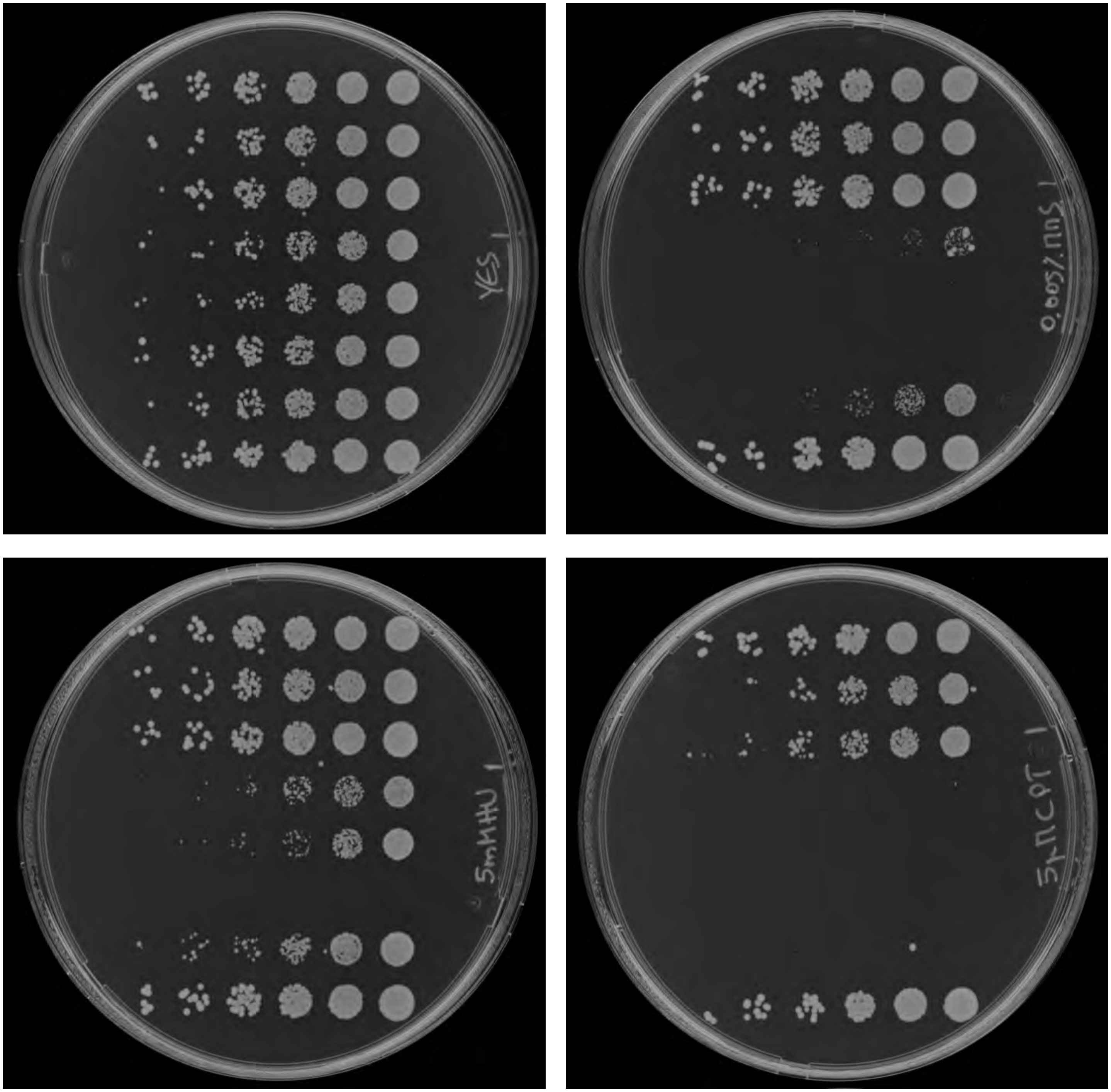

Fig S4B

ssb3-GFP

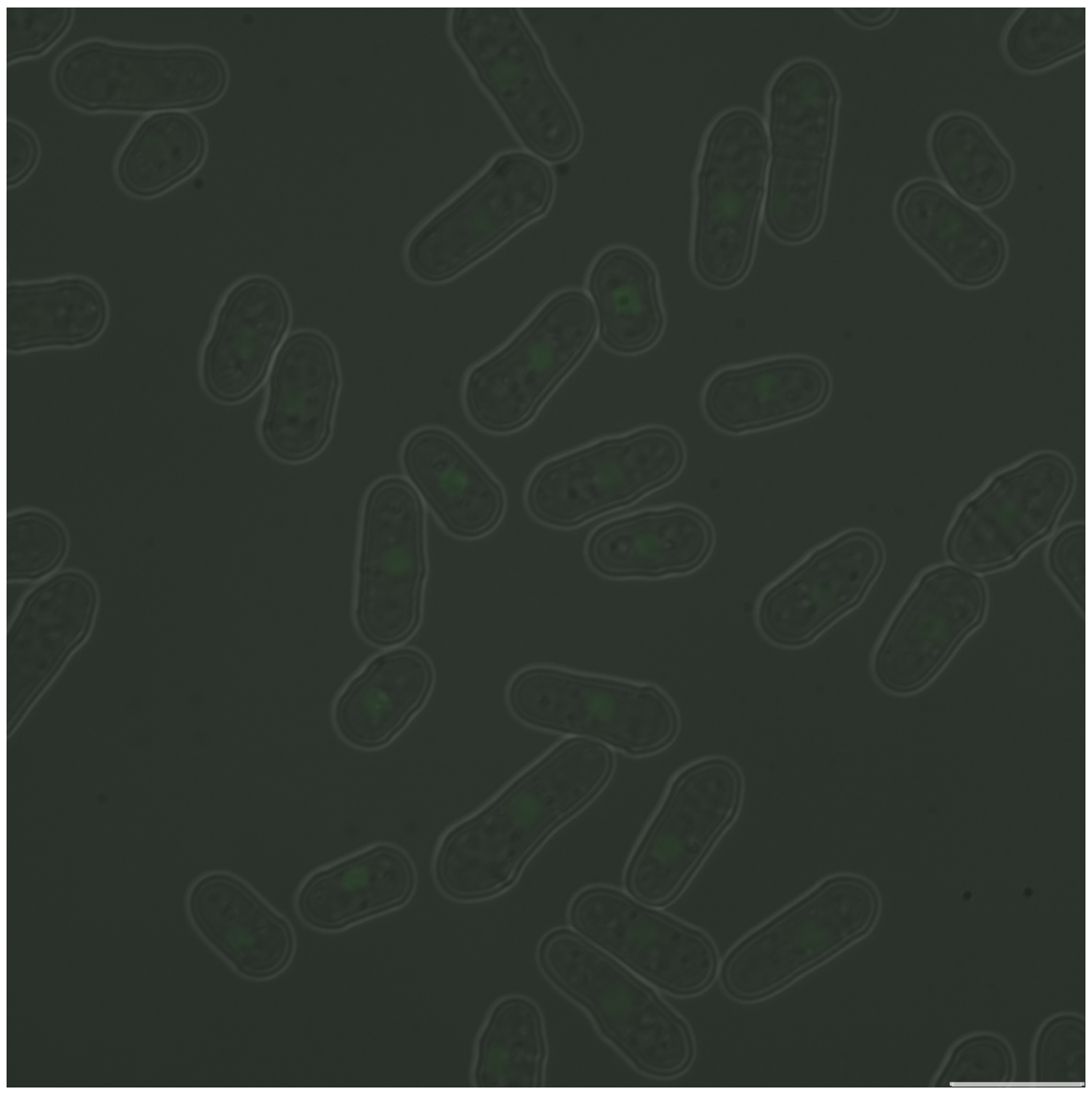

ssb1^{+} (wt)

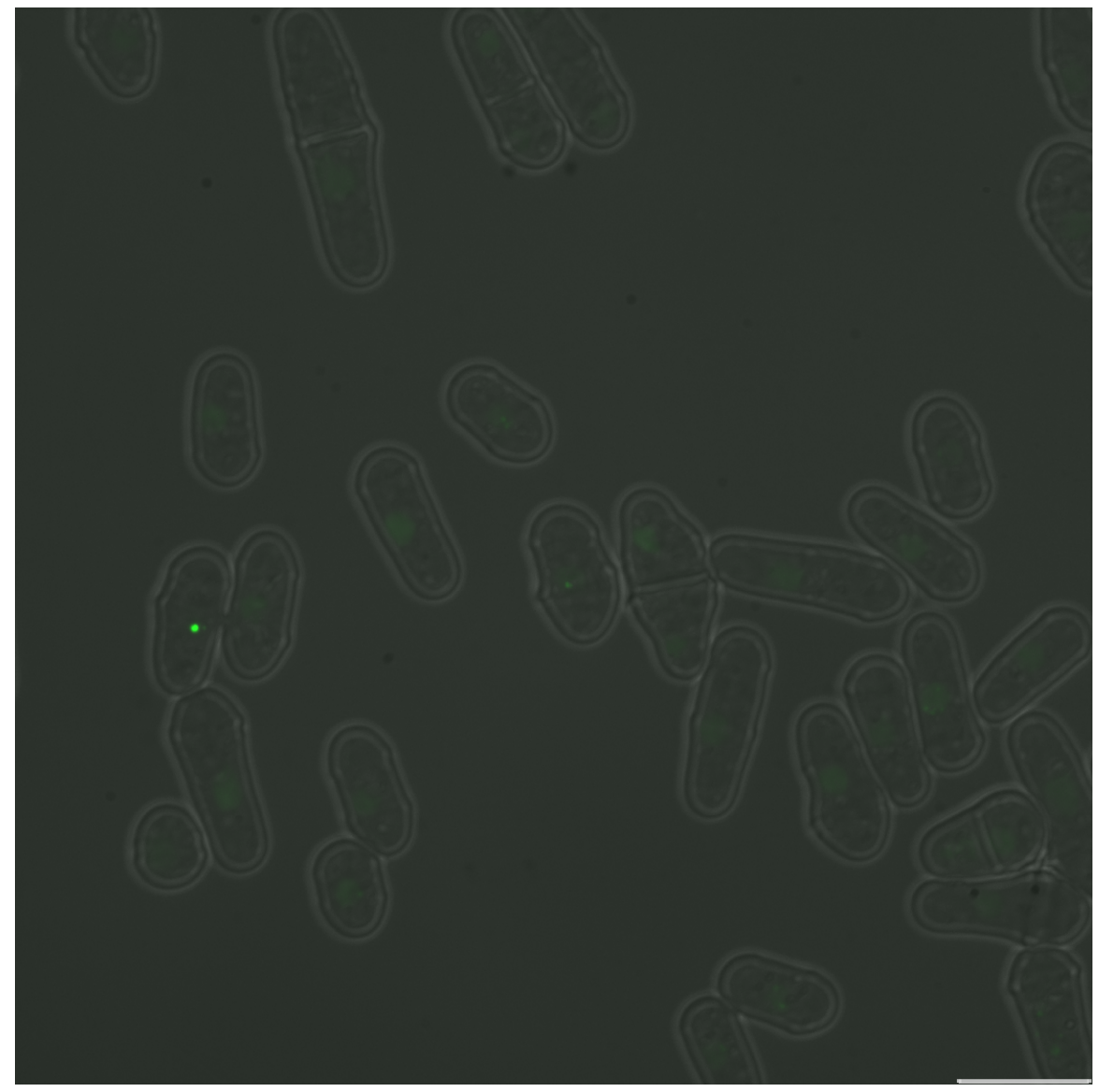

ssb1-W523C (1x)

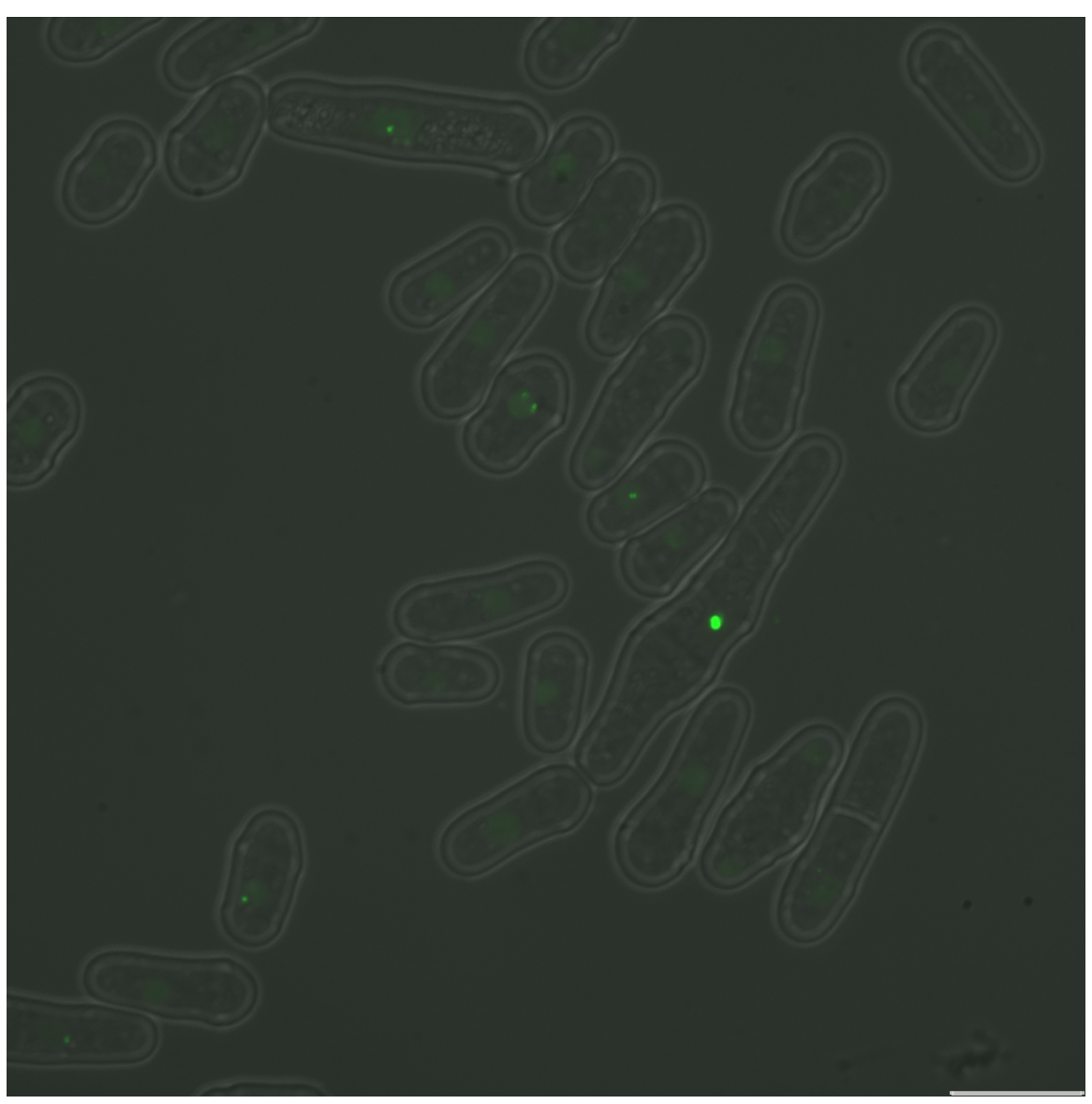

ssb1-W523C (6x)

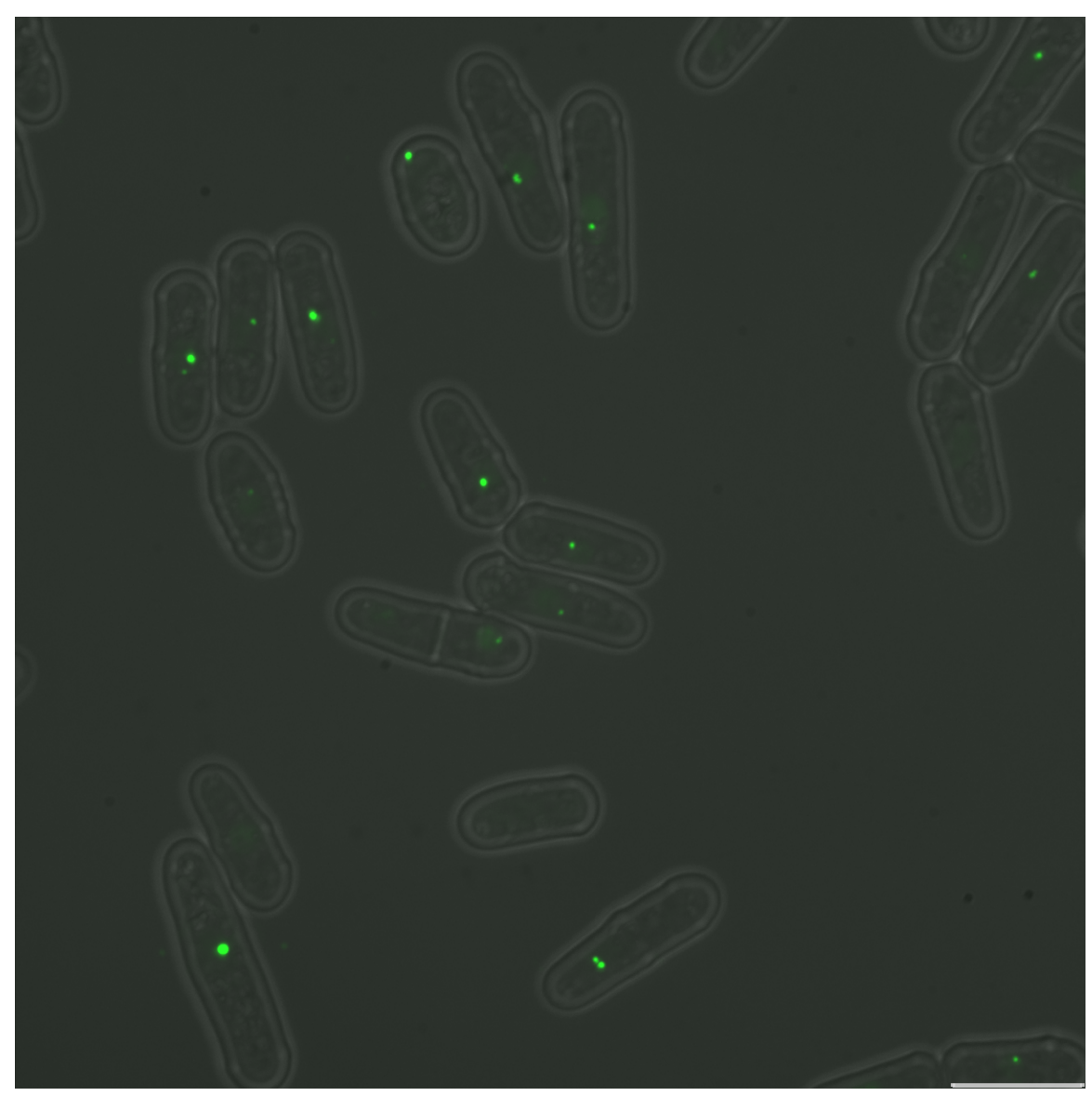

ssb1-W523C (13x)

Fig S5B

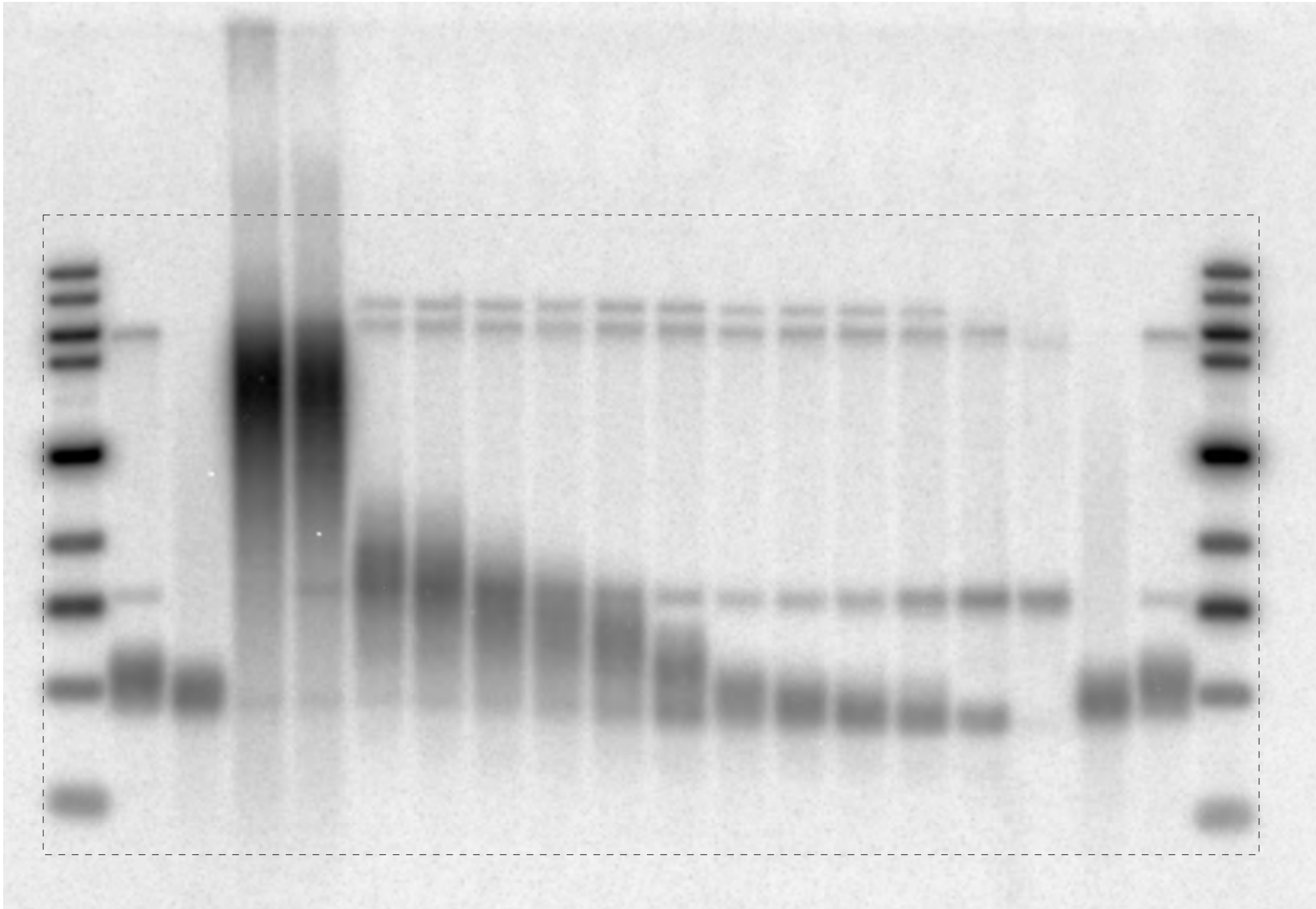

Fig S5C

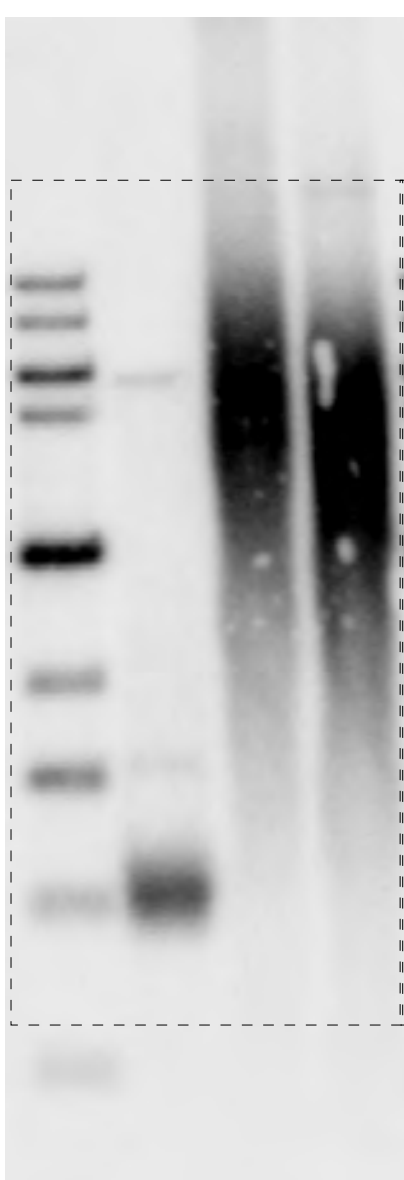

Fig S5D

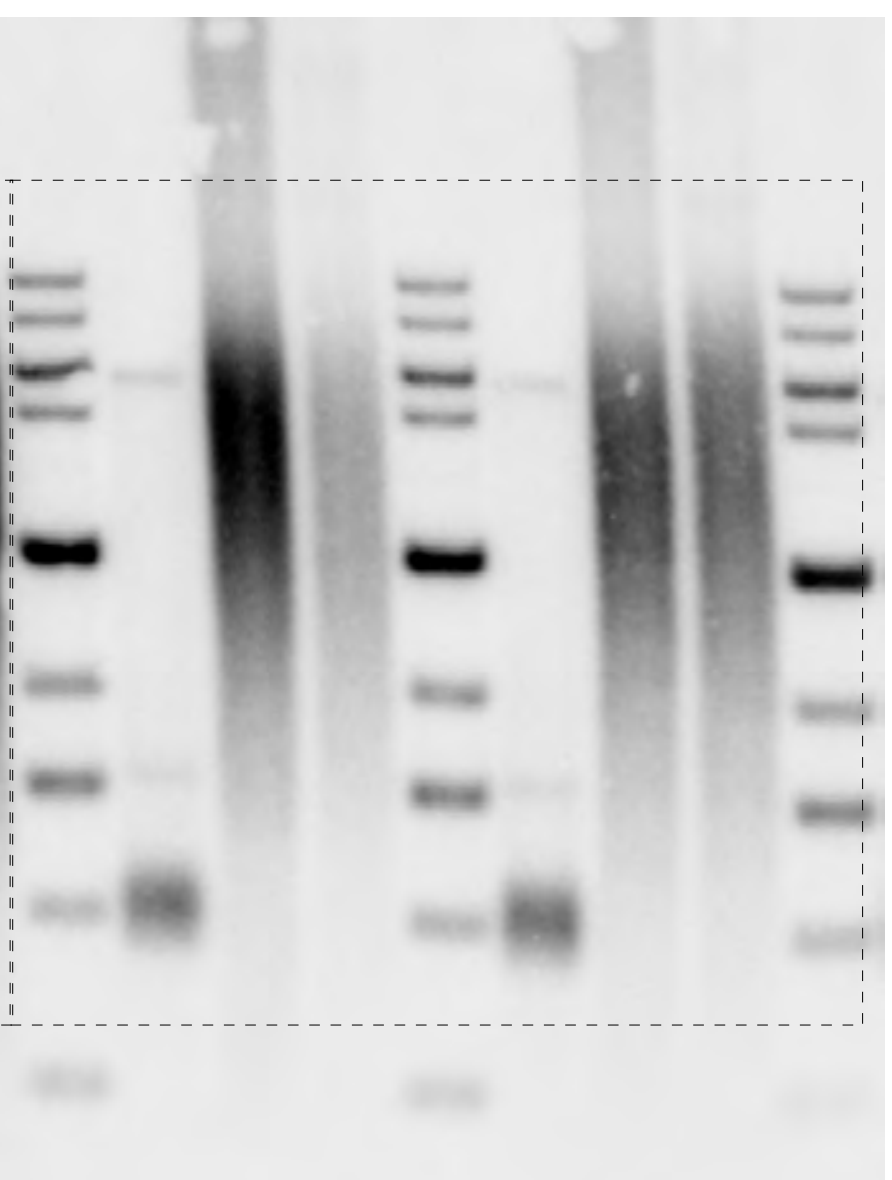

Fig S5E

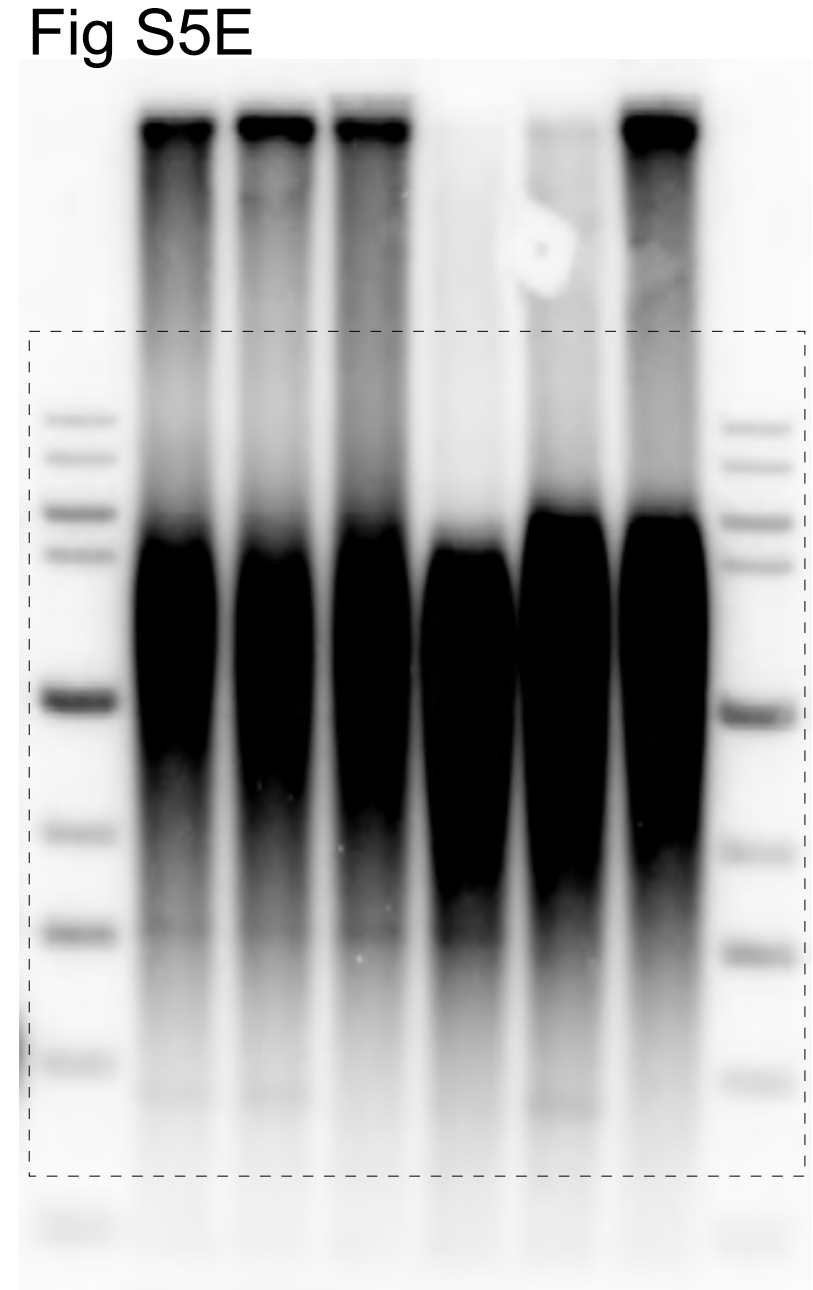

Fig S7B

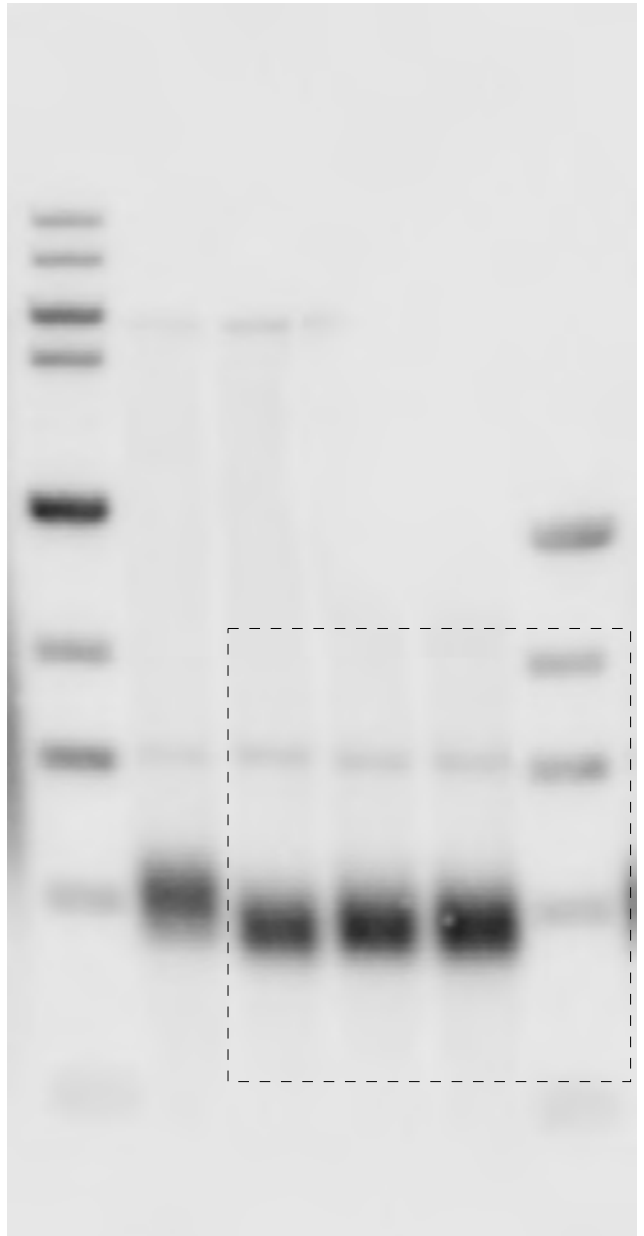

Fig S7C

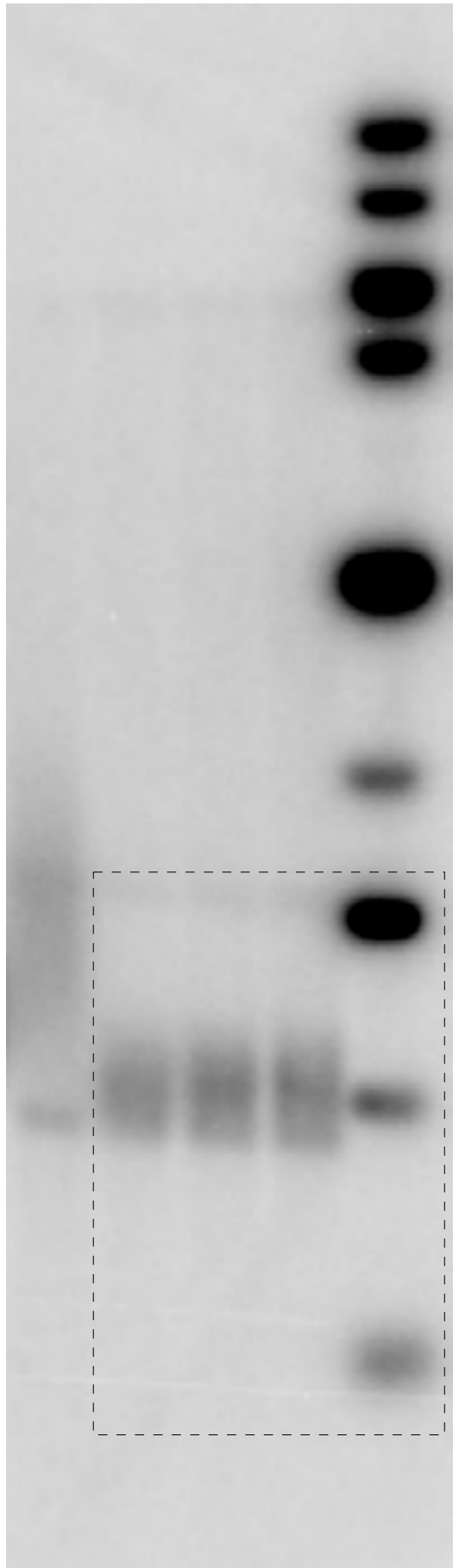

Fig S8A

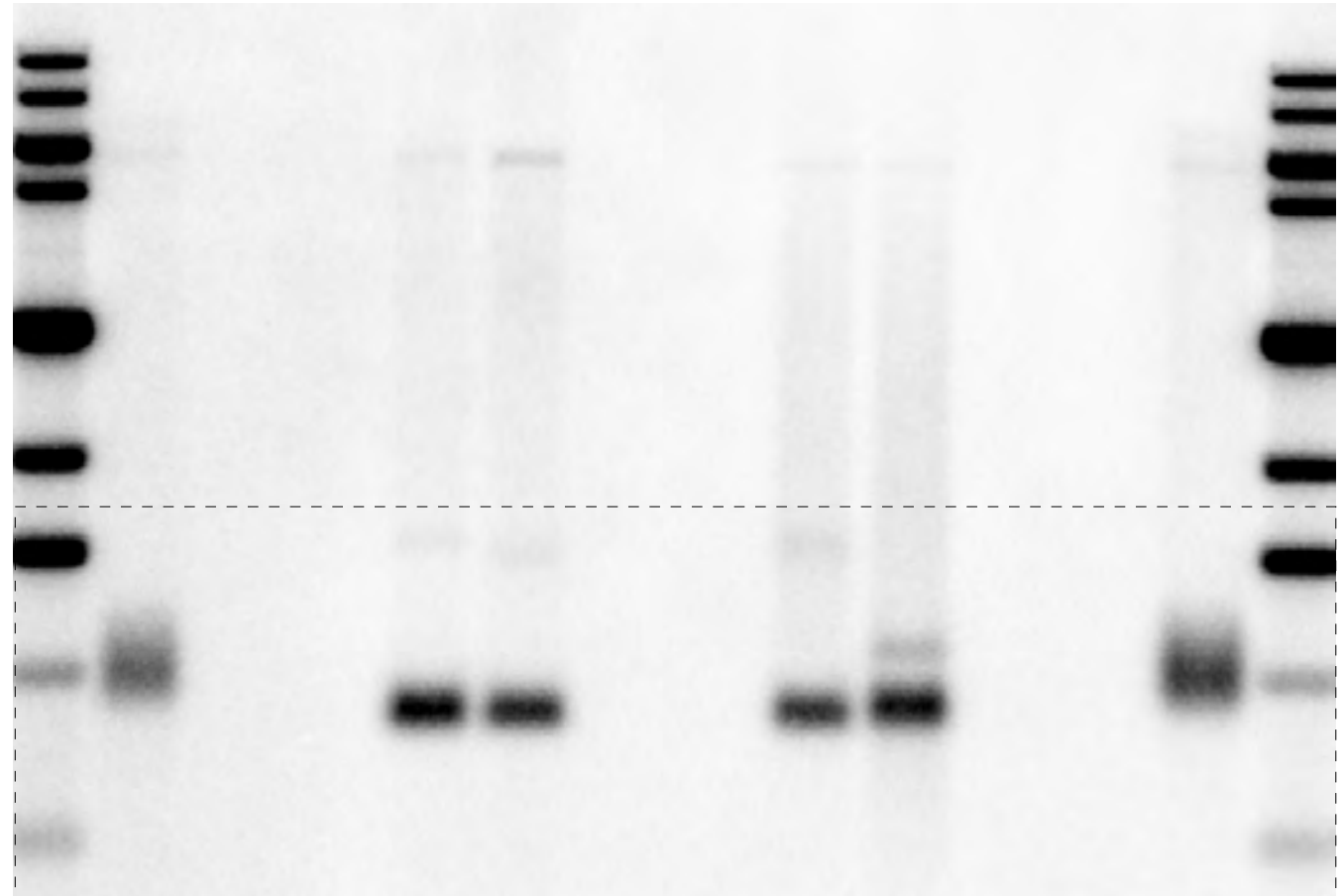

Fig S8B

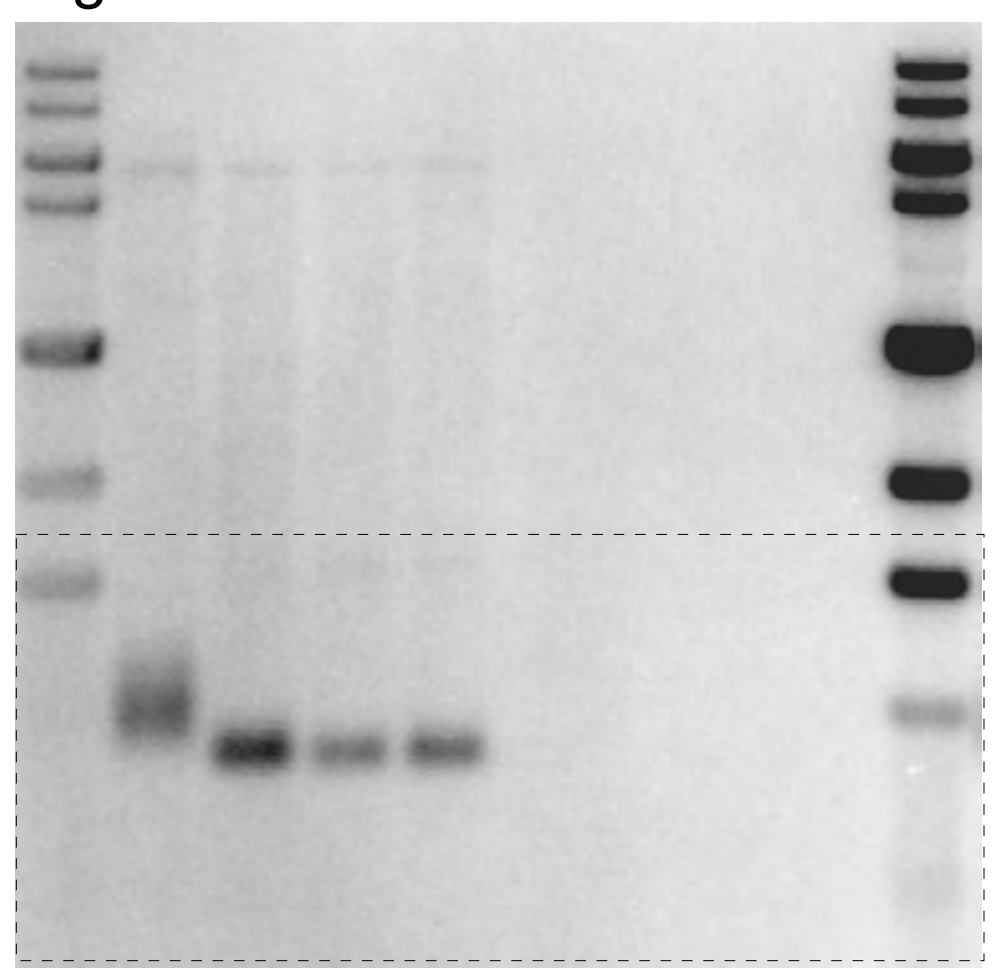

Fig S8C

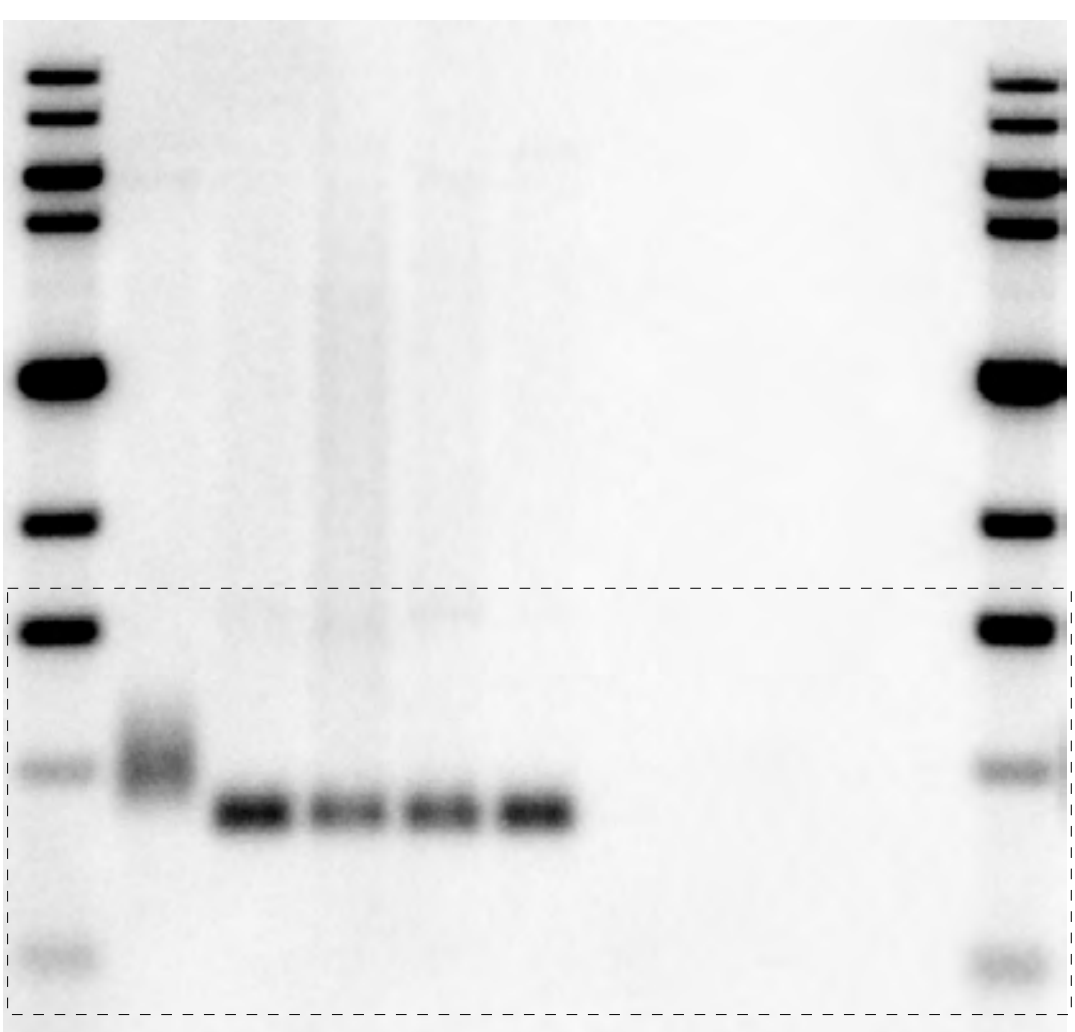

Fig S8D

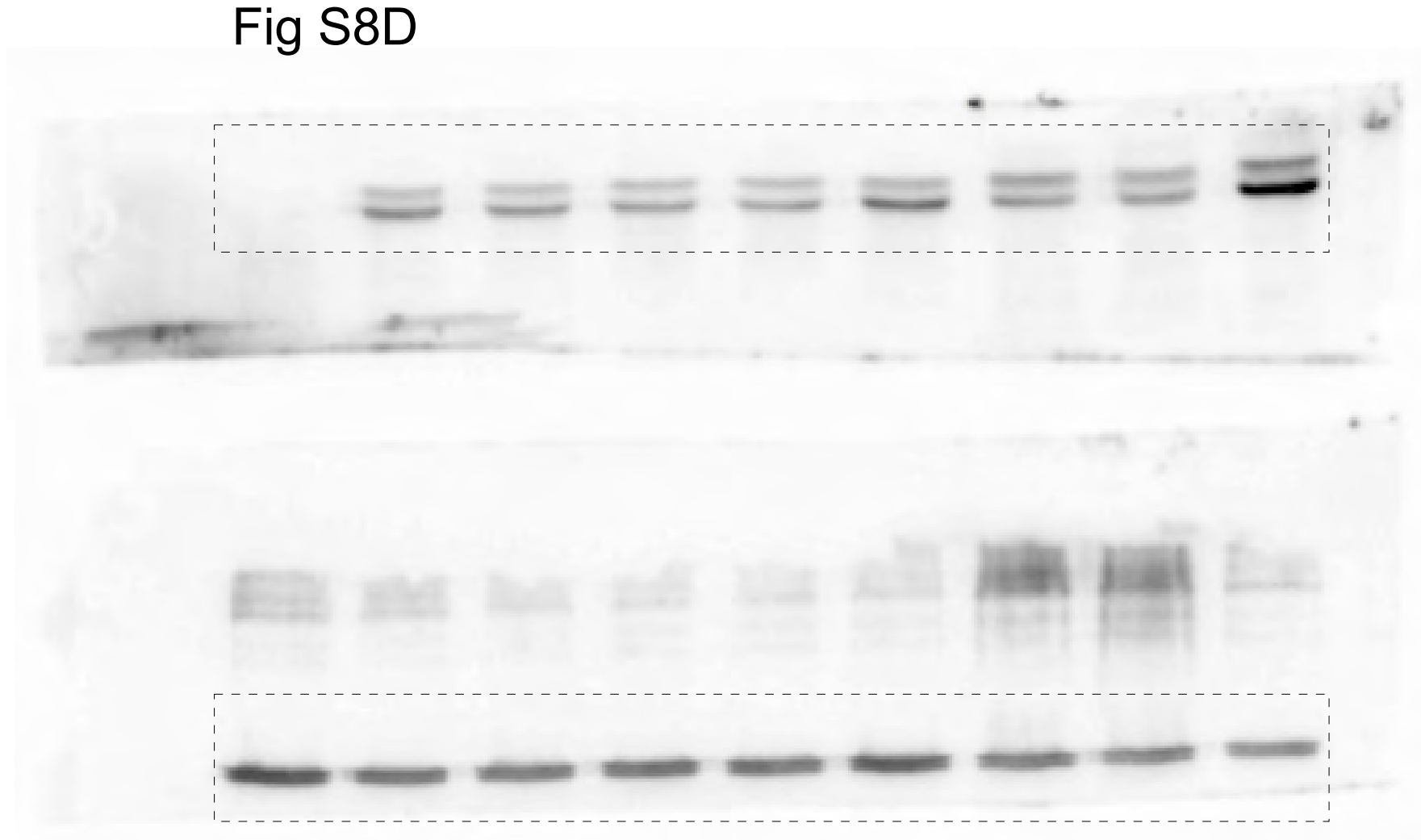

Fig S8G

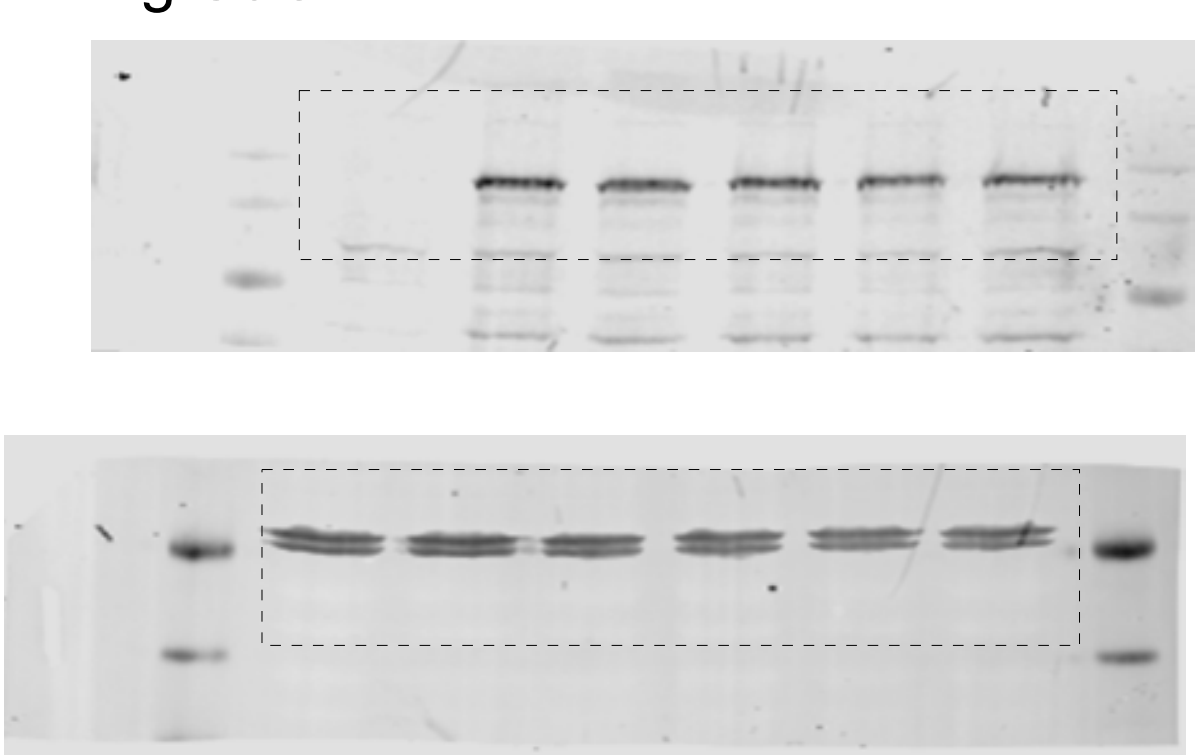

Fig S8E

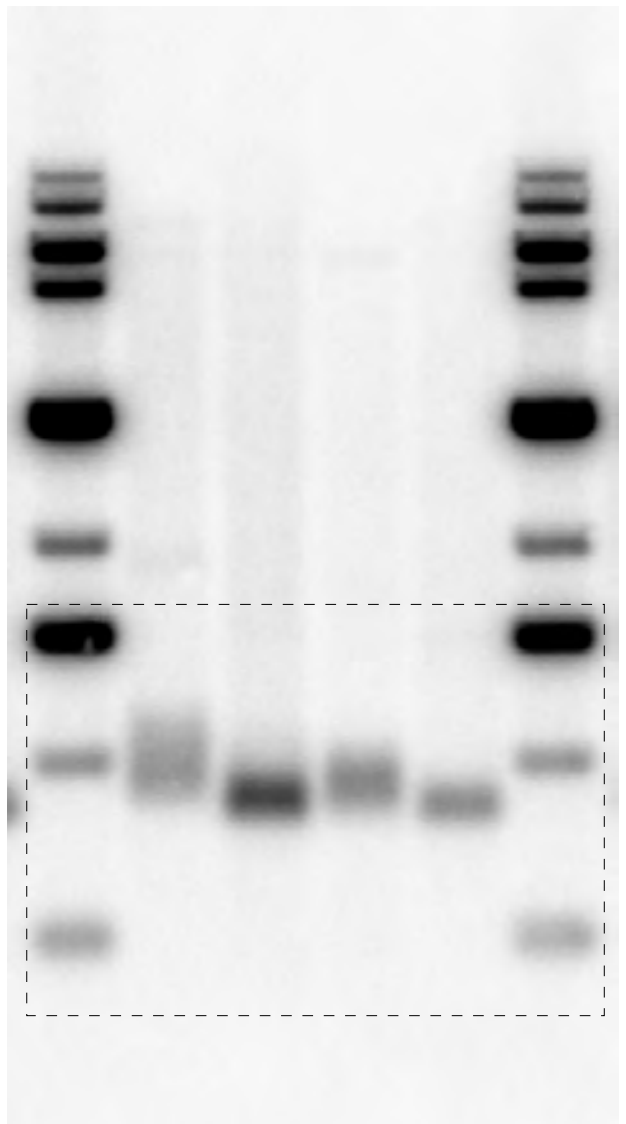

Fig S8F (left)

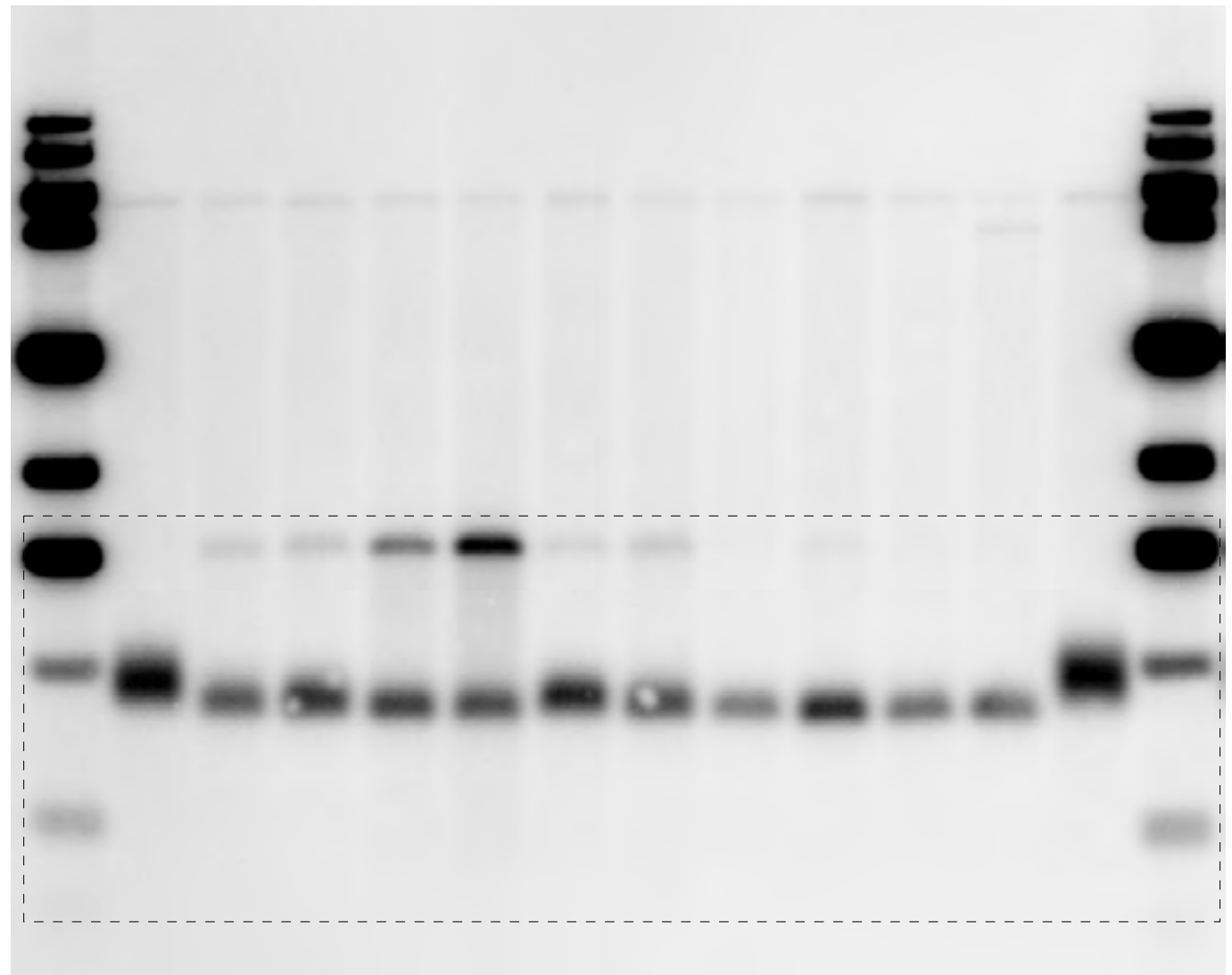

Fig S8F (right)

Fig S8H (right)

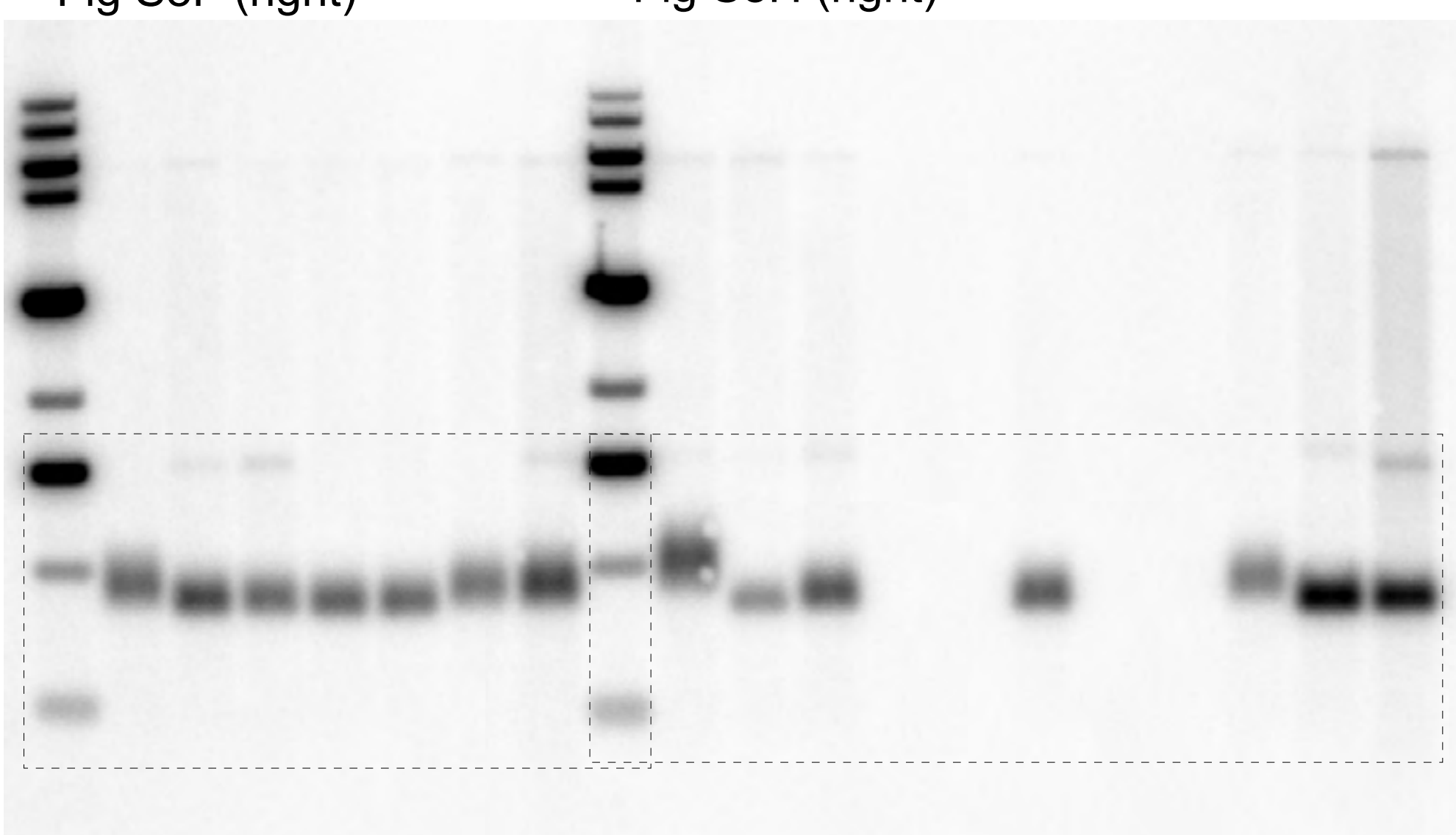

Fig S8H (left)

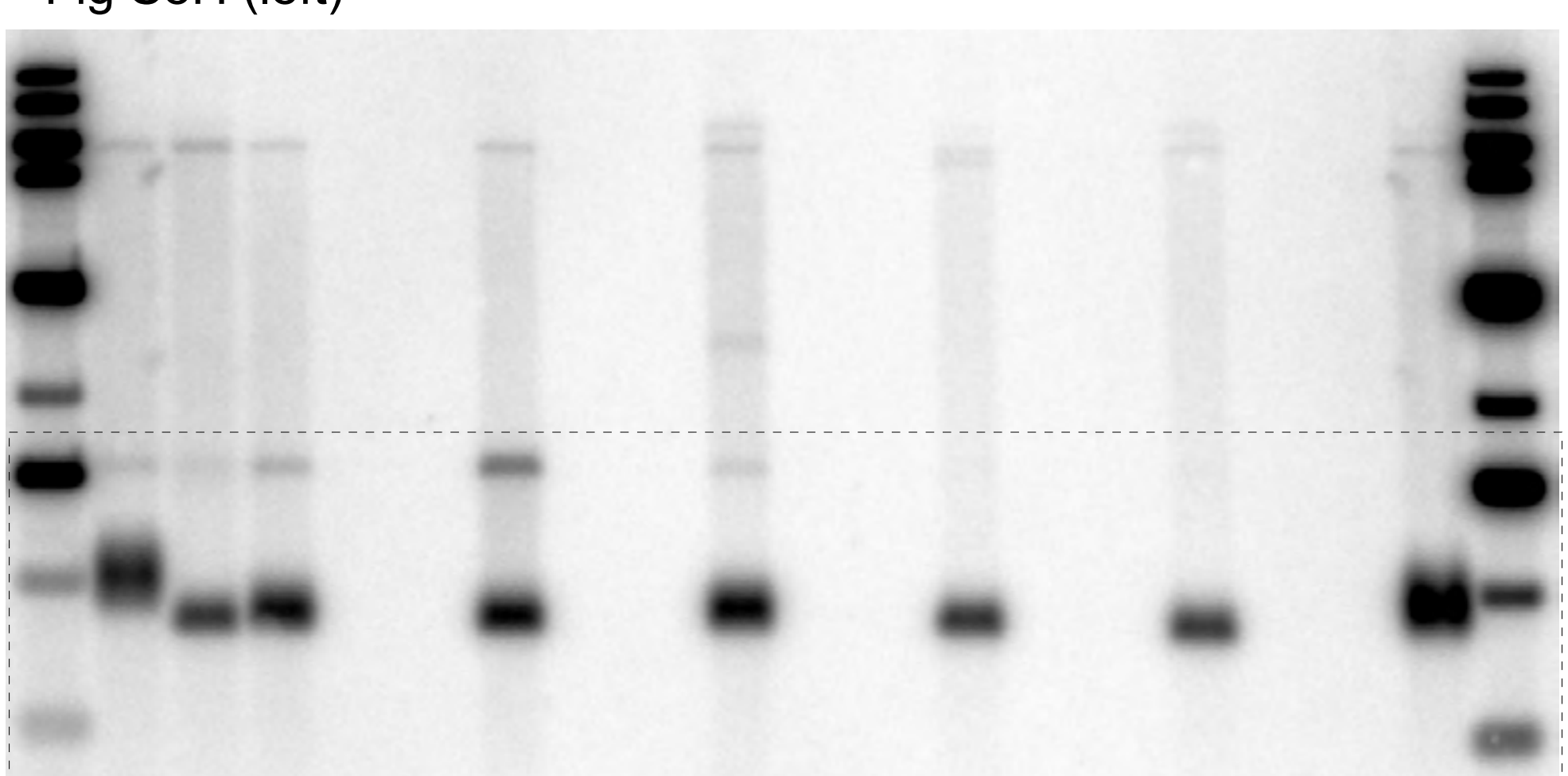

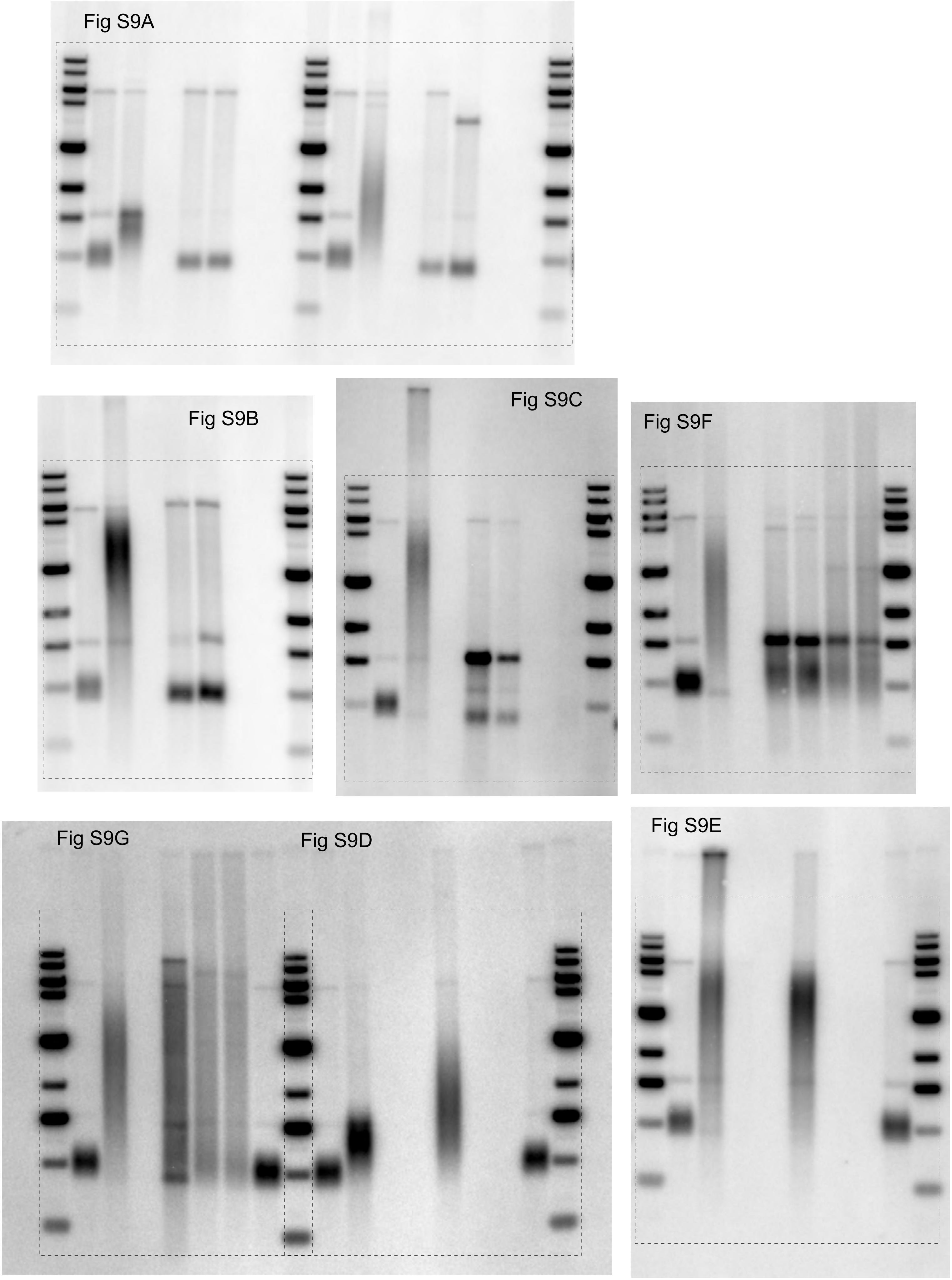
Fig S9A
Fig S9B
Fig S9C
Fig S9F
Fig S9G
Fig S9D
Fig S9E

Fig S10

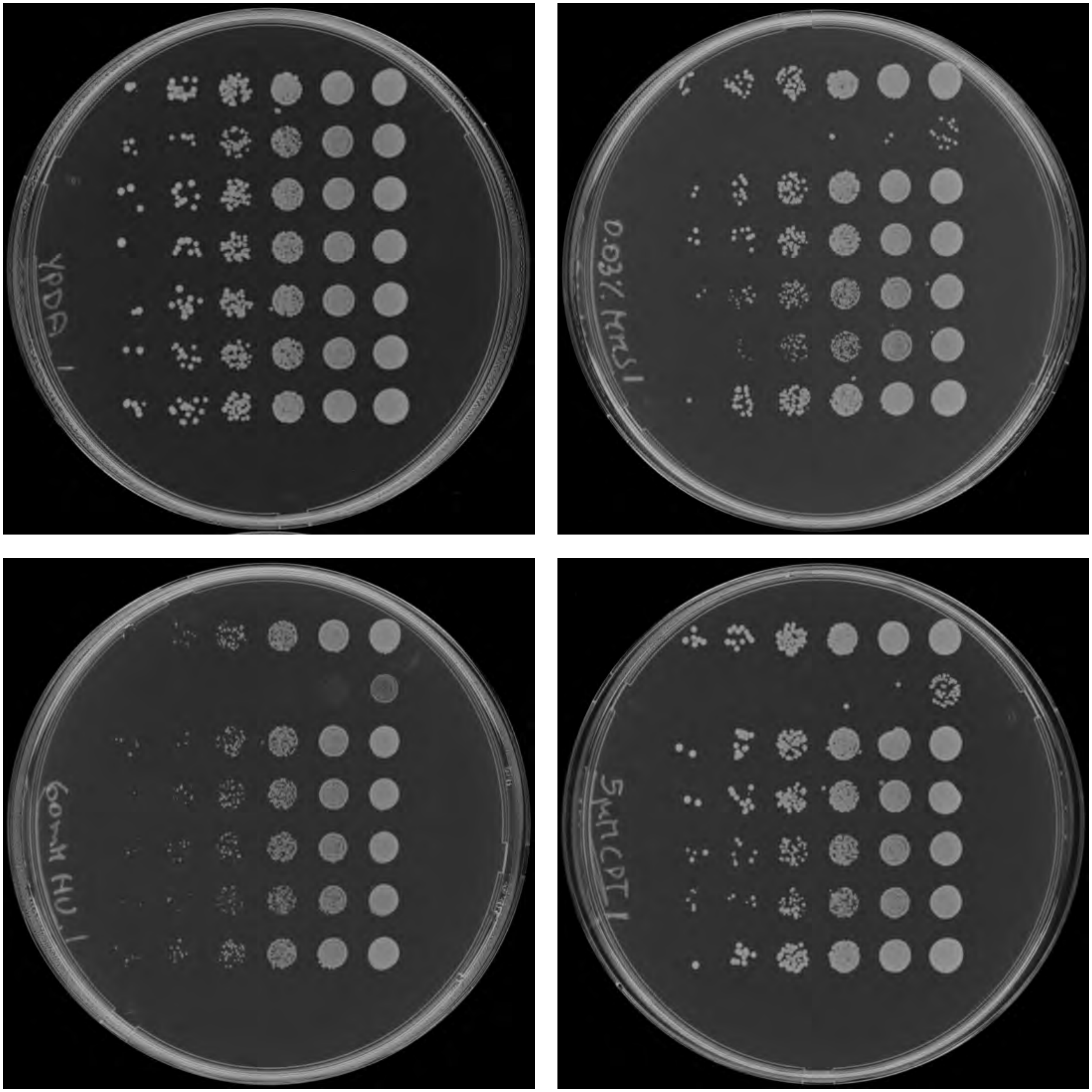

Supplement: Supplementary file 2 [file pgen.1012297.s022.pdf]
