# Supplementary material for: Fission yeast RPA–TERT–Tpz1TPP1 complex promotes telomere extension and suppresses telomere recombination
Source: PLoS Genet. 2026 Sep 8;22(9):e1012297. doi: 10.1371/journal.pgen.1012297 (PMC13577582; doi:10.1371/journal.pgen.1012297)
Supplement: Supplementary file 4 [file pgen.1012297.s028.docx]

**Reviewer #1:** In the manuscript, Fission yeast RPA–TERT–Tpz1TPP1 complex promotes telomere extension and suppresses telomere recombination, the authors examine the role of the single-stranded DNA binding protein RPA in telomerase activation at fission yeast telomeres. While a role for RPA has been demonstrated in budding yeast and human cells, this manuscript is the first to characterize these interactions in fission yeast. The authors take advantage of AlphaFold 3 modeling combined with related experimental structures and existing genetic characterization across multiple species to propose interactions between the catalytic subunit of telomerase, the TPP1/Tpz1/Est3 protein, and the largest and second largest subunits of RPA that appear to facilitate telomerase action at telomeres. The authors present an analysis of an impressive number of mutants with characterization of telomere length, protein association with telomeres by ChIP, and expression levels. In some cases, reciprocal suppression and epistasis experiments support the proposed model. The work expands our understanding of interactions that regulate telomerase activity at telomeres and the analysis of similarities and differences between human telomerase and major model systems is useful and informative.

While the experiments are extensive and the results are presented clearly, in some cases it is difficult to ascertain reproducibility and no statistical tests are presented to support claims. Additional quantification and attention to statistical significance is warranted. This is particularly true for the ChIP experiments since the analysis of Trt1 association with telomeric DNA underpins a major conclusion of the paper that the interactions analyzed primarily affect telomerase action and not recruitment.

We thank Reviewer #1 for a positive overall assessment of our manuscript. As detailed below, we now provide raw data points and statistical analyses for all our ChIP assays in the supplemental S1 Data Excel sheet.

**Major comments:**

1. In cases where telomere length is measured, the number of times independent cultures were analyzed should be indicated (at least in cases where only a single sample is shown). Ideally, telomere length should be quantified to demonstrate reproducibility and the extent of the effect, particularly when the changes are subtle.

For Southern blots, where changes in telomere length were examined, we have shown 2 independent clones (or in some cases more clones) per given mutation in all cases (Fig 1B, 4C-E, 4G, 5C-D, 5F-H, 6B, S1D-E, S2D, S2F-H, S7C, S8A-C, S8F, S8H, S9A-G / Note: these panels numbers refer to figure numbers in the revised manuscript, as some numbers have shifted between original submission and revised manuscript), except for Fig 1C.

For Fig 1C, we chose to show one of 2 clones we ran on a Southern blot in our original submission because of the way we ran this gel. We did not feel it was necessary to show essentially identical results in 2 separate panels and wanted to save space. However, in response to the question raised by Reviewer #1, we have now restored the Southern blot results for the 2nd independent clone in the new Fig S2B. (See also S2 data for the original full gel where these 2 panels are derived.) It should also be noted that telomere length for these ssb1 mutations shows a similar pattern of shortening for these alleles in Fig 1D, where trt1-myc is additionally introduced.

The only other panel in which we showed results from a single clone per mutation was Fig S7B, which included ssb2(1-180)-FLAG and ssb2(1-210)-FLAG. For these clones, we obtained only a single clone per allele, but we did not pursue further, as they did not affect telomere length for both of those truncation constructs compared to the ssb2-FLAG full-length clone.

Southern blots shown in association with ChIP results (Figs 1D, 2A-G, 4B, 4H-I, 5A-B, S8E) were primarily used to aid interpretation of the ChIP results; therefore, the strains (or restreaks of isogenic strains) used for the ChIP assays are shown. For those gels, telomere length is not necessarily terminal length, but rather often in the process of changing. While we have generated multiple clones for strains with the indicated genotypes and analyzed telomere length in genetic crosses prior to choosing a given strain for ChIP assays, the whole point of these Southern blots was to indicate telomere length for each chosen strain per genotype.

2. 142-144: Authors state, “Surprisingly, D235K led to a slight increase in telomere length.” Figure S1E. If there is an increase, it is very hard to discern given that the markers didn’t run the same amount on the two sides of the gel and these samples are in the middle (and the two colonies don’t look the same). This example illustrates why telomere lengths should be quantified throughout the manuscript.

We agree that the change in telomere length for D235K is very small and very similar to that observed in cells expressing the wt ssb1 plasmid. The plasmid-based assay was performed for D235K primarily to determine whether this mutation might cause telomere shortening, as in human cells. Since we did not observe shortening, we did not pursue further.

Since this mutation is not the main point of the paper, and speculation regarding increased RPA binding is probably premature at this time, we have decided to simplify our statement regarding this mutant as follows: *D235K* did not cause telomere shortening (Fig S1E).

Except for D235K, we focused on clones that showed significant changes, and we feel those changes are obvious without quantification. Therefore, we find it unnecessary to quantify all Southern blots shown in the manuscript in order to draw conclusions about the importance of the analyzed mutated residues in telomere maintenance.

3. The authors should state more clearly what the n values are for the ChIP experiments and what each point on the graph represents. It looks to me like there are often different numbers of points for different restreaks (Figure 2D-G). Why is that? I assume that the Southern blot is taken from one repeat? That should be stated.

4. ChIP assays require statistical analysis to support the statements made, with appropriate adjustment for multiple comparisons.

To address major points #3 & #4, we now include the S1 Data supplemental Excel sheet, which contains experimental values for individual data points used to generate ChIP assay plots and all possible pairwise comparisons by 2-tailed Student's t-tests, as well as one-way ANOVA with post-hoc test with Dunnett’s multiple comparison test.

For a given ChIP experiment, n values are not always identical because some samples were lost during ChIP processing, or, in some cases, different numbers of independent cultures were set up for a given strain. For telomere shortening, the ChIP series shown in Figs 2A-G, earlier-generation strains, or certain restreaks have more n, as we ran test ChIP assays with fewer selected restreaks in initial test runs, which were combined with later ChIP repeats that included additional restreaks.

As already mentioned in response to major point #1 raised by Reviewer #1, the Southern blots shown alongside the ChIP assays were included to primarily aid interpretation of the ChIP results, since recruitment of RPA and Trt1 is telomere-length-dependent. Thus, while each blot includes a single clone for a given ChIP genotype, we ran multiple Southern blots across multiple isogenic strains to help guide the choice of strains for ChIP assays. Figure legends for those ChIP-associated Southern blots mentioned that “corresponding telomere length analysis by Southern blot of the strains used for ChIP” were shown. On the other hand, including these details for every strain not shown or further analyzed seems unnecessary, and thus we chose not to add these details in main text of the revised version.

5. Why are the protein expression levels not quantified in S2B? Even with only 2 samples, it would be useful to have the quantification given.

We have now quantified Fig S2C (S2B in the original submission); the results are shown in the Western blot gels below.

**Minor comments:**

1. In experiments like those shown in Figure 2D, it would be helpful to be clearer that the mutant trt1 protein is the Myc-tagged version that is being immunoprecipitated. As shown, the labeling seems to indicate that WT trt1-myc is being expressed in these cells.

We assume that Reviewer #1 meant Figure 2E, as ChIP shown in Fig 2D utilized wild-type Trt1-myc only. To help clarify that we used Trt1-myc or Trt1-D743A-myc for ChIP, we modified the panel to indicate “Trt1-myc ChIP (wt or D743A)” on top of the graph for Figure 2E.

2. In Figure 5, it would be very helpful to color code the different interactions to help the reader keep track.

While we did not color-code it, we modified Fig 5C & 5D to indicate which interface was predicted to be disrupted by a given mutation.

3. 6E needs to be done additional times and statistical analysis done before claiming there is a difference.

We performed additional replicates of Fig 6E-G (human telomerase stimulation assay by RPA) and also provided raw data values for quantification and statistical analysis in S1 Data Excel sheet (n =4).

4. Figure 6J: spiens rather than sapiens

Thank you for pointing out this error. It has been fixed.

**Reviewer #2:** Recent studies have demonstrated that RPA activates telomerase in humans and budding yeast by interacting with TERT and TPP1 orthologs, indicating a direct role of RPA in its activation. In this study, Moser and colleagues used genetic and structural modelling to demonstrate the formation of a ternary RPA–Trt1–Tpz1 complex in fission yeast. This is interesting work that brings new insights into the mechanism underlying RPA-driven telomerase activation in the light of previous studies. However, some conclusions are sometimes reached without accounting for all possible hypotheses (e.g. for W523C mutant). A large number of mutants have been tested, which makes the reading difficult to follow. I would recommend to remove some data to lighten the study and to focus on the most interesting results that support the ternary RPA–Trt1–Tpz1 complex. I raised several points that should be addressed before publication.

-Mutants L222A, D235K and T265A that are equivalent to mutation associated with telomeropathies in human have been tested in fission yeast. D235K led to a slight increase of telomere length (TL). Their interpretation is that gain of function of D235K increase RPA-Rad3 association. In contrast, the L222A which is also a gain of function mutant shows a modest shortening. Since the affinity for ssDNA have not been tested for L222A, D235K and T265A mutants, I will be cautious with the Rad3 interpretation that is contradicted by the L222A mutant. Moreover, the genetic analysis of these mutants adds little to the article’s main focus and can safely be omitted if not developed further.

As mentioned earlier in response to Reviewer #1, we have removed speculation regarding increased RPA-ATR binding or telomere elongation in D235K, and it now simply states that it did not cause telomere shortening.

We would like to keep the results shown in Fig S1E, since L222A, which was not found in our genetic screen, was further characterized by mutant allele integration, and it is difficult to explain why this mutation was included in these analyses without showing plasmid complementation results for characterization of human RPA mutant equivalent strains shown in Fig S1E. While we agree these mutants were not central to this paper, they are shown only in the supplemental figure for this exact reason.

-“Chromatin immunoprecipitation (ChIP) assays for Trt1 detected moderate increases in telomere binding for all mutants except Y474N, which showed slightly reduced binding (Figs 1D and S2D). Thus, telomere shortening in these strains is unlikely to result from reduced telomerase recruitment and instead reflects impaired telomerase-mediated extension.”

In figure 1D, telomerase recruitment has been tested in rpa1 mutants with short TL. I agree that telomerase seems to be recruited at a higher level than WT (although we miss statistical analysis here), however one possible explanation for this increase would be that telomeric replication stress provoked by Rpa1 mutations causes telomerase recruitment, since it has been shown that telomerase can be recruited at stalled or broken replication forks.

The important point we can draw from these results is that a loss or reduction of telomerase binding cannot account for the telomere shortening observed in these mutants, and we believe this conclusion is fully supported by our analysis.

We do not quite understand Reviewer #2’s point regarding how replication fork stress increases telomerase binding in these mutants. It could be, but why would that be relevant here? Importantly, these RPA mutants, despite showing a statistically significant increase in Trt1 binding (except for Y474N and K484E, where changes from wt ssb1 were deemed not statistically significant), fail to maintain wt telomere length.

This is particularly true for the W523C mutation for which the authors conclude that “Taken together, these results identify ssb1-W523C as a separation-of-function mutant that uncouples telomerase recruitment from telomerase activation.” According to the Alphafold model, this residue contacts ssDNA and previous published results from the Nakamura lab showed that this mutant strain is sensitive to replication stress (HU and MMS), indicating that this substitution highly likely affects ssDNA properties of RPA. (ssDNA binding assays and DNA 2D-gel electrophoresis might eventually be done to demonstrate the neutrality of this mutation). Thus, it seems to me that this mutant it clearly not a separation-of-function mutant. On the top of that, the equivalent mutation of W523C (RPA1-W528C in human) has a modest effect on telomerase processivity (Fig 6E). This mutation is somehow reminiscent to D223Y mutation that impairs ssDNA binding properties of RPA, cause sensitivity to DNA damaging agents and provokes the accumulation of RPA at telomeres.

It is likely that the amount of RPA detected by ChIP at telomeres in this study reflects mainly the level of RPA recruited during replication at forks and not the level of RPA that act as telomerase-processivity factor, leading to a misinterpretation of the results.

We respectfully disagree with Reviewer #2’s assertion that *ssb1-W523C* does not represent a separation-of-function mutant, and so it does not add anything new compared to the telomere defect in the *D223Y* mutant, in terms of showing increased telomerase recruitment at telomeres.

First, as shown in our new data (Fig S4A), early generation *ssb1-W523C* mutant cells do not show hypersensitivity to various drugs (HU, MMS, or CPT), and only *ssb2-W523C* mutant cells with circular chromosomes, much like *trt1∆* cells with circular chromosomes, show sensitivity to these drugs. By contrast, *ssb1-D223Y*, as previously established, is very sensitive to these drugs and drastically reduces the expression level of RPA, so the phenotype observed for this mutation is highly pleiotropic, yet the telomere defect is arguably less severe than that of the *W523C* mutant.

Second, we have now monitored foci formation by Ssb3-GFP in the *ssb1-W523C* strain background with differing telomere length (Fig S4B-C), and found that cells that carry longer telomeres show a comparable spontaneous Ssb3-GFP foci frequency as ssb1 wild-type cells, while progressive telomere shortening is accompanied by a progressive increase in Ssb3-GFP foci formation frequency. These results clearly indicate that the *ssb1-W523C* mutation itself does not cause a global defect in DNA replication or DNA damage responses, but any increase in RPA binding observed in this mutant is due to telomere shortening. Since this mutant appears to be primarily defective in telomere maintenance without causing much defect in other functions of RPA, this is truly a remarkable and clean “separation-of-function” mutant.

Third, since RPA or telomerase recruitment to telomeres in *ssb1-D223Y* cells has only been monitored in cells with already substantially shortened telomeres, it is also possible that even for this mutant, if we were to take extra effort in generating *ssb1-D223Y* strains with longer or comparable telomere length as wild-type cells, they will not show much increase in RPA or Trt1 recruitment. Hence, the observed increased bindings of RPA and Trt1 may be secondary consequences of having shorter telomeres, even for *ssb1-D223Y*.

In addition, “A similar pattern was observed in the catalytically inactive trt1-D743A mutant (Fig 2E), indicating that ssb1-W523C cells are not defective in telomerase recruitment, but instead fail to engage in productive elongation of telomeres.” I don’t understand this conclusion. Comparing Fig 2D and 2E, it seems clear that Trt1-D734A exacerbates the shortening of telomeres, clearly indicating that W523C mutant shortens its telomeres not only because of a defect of telomerase activation

Our point of comparison to catalytically dead Trt1 was that the *ssb1-W523C* strain, like the catalytically dead *trt1-D743A* strain, shows a progressive increase in telomerase binding, followed by a loss of telomerase binding at telomeres as telomeres become critically short. We are not claiming that *trt1-D743A* and *ssb1-W523C* have identical telomere shortening rates or are completely equivalent. In fact, our suppressor analyses with *poz1∆*, etc. (Fig S9), indicate that the inability of the *ssb1-W523C* strain to maintain telomeres can be overcome by removing the inhibitory function of shelterin. In addition, the results shown in Fig 5F indicated that disruption of *ssb1-W523C* can suppress the ability of Trt1-D743A to prevent HR-dependent telomere maintenance, so it makes total sense that Trt1-D743A cells have a faster senescence rate compared to *ssb1-W523C*. (In fact, we would predict that *trt1-D743A* *ssb1-W523C* cells would show slower senescence since Trt1-D743C can no longer effectively interfere with HR at telomeres.)

In conclusion, I found the analysis of W523C mutant uncomplete and misinterpreted, burdening this work. I would recommend to focus the work on residues that alter Rpa1-Trt1 and Rpa2-Tr1t interactions.

As we argued above in rebuttal of Reviewer #2’s claims, we believe *ssb1-W523C* represents a clean separation-of-function telomere-specific defect mutant with remarkably strong and specific defect in telomere maintenance. As such, we cannot disagree more with the suggestion of entirely removing this most interesting mutant from our current study.

-line 253: tpz1-E74 instead of tpz1-K74?

Yes. It should have been *tpz1-E74R*. It has been corrected.

-Again, I advise caution in interpreting the Rpa2-W112A data, since this W112 contacts ssDNA. Overall, It is intriguing that ssDNA contact residues are also involved in Trt1 interaction. Does it reveal a change in the mode of binding of RPA to ssDNA to accommodate telomerase association.

Our model is indeed that the RPA-Trt1-Tpz1 complex formation affects how RPA interacts with ssDNA and may help free the 3’ end so it can be guided to the catalytic center, and this has already been discussed at length.

-Direct telomerase assay in Fig 6E showed that the equivalent mutation of W523C (RPA1-W528C) has a modest effect on telomerase processivity. Thus, it seems contradictory with the separation-of-function W523C mutant.

Does the equivalent mutation of Rpa2-W112A has been tested by telomerase assay?

While the RPA1 W528C mutant had a milder defect in RPA-dependent stimulation of telomerase activity than the RPA2 W107A (*ssb2-W112A* equivalent), as shown in the same gel and previously published, it does have a measurable effect, and such a result does not “contradict” any conclusion with regard to this mutation contributing to RPA-TERT-TPP1 complex function. In addition, our new replicates and statistical analysis (please see our updated Fig 6F and S1 Data) showed that the alanine mutant (W528A) has a stronger effect and a similar effect to RPA2 W107A, which further supports our interpretation that W528 plays a role in RPA stimulation of human telomerase processivity. We have added additional sentences in the result section, lines 450-454, to emphasize this point.

-Budding yeast data doesn’t seem to bring more insights. This can be removed to lighten the work.

We respectfully disagree. Budding yeast data indicate that an equivalent residue of ssb1-W523C has an important function in budding telomere maintenance, which adds significantly to the notion that conserved equivalent interaction between RPA1 and telomerase catalytic subunit is important for telomere maintenance in both budding yeast and fission yeast.

- One aspect that is not discussed here is that RPA is mainly recruited at telomeres in S-phase than much earlier telomerase (shown by the Nakamura lab EMBO 2009). How does this fit with the model presented here?

Again, a main concern here is that asynchronous ChIP of RPA at telomeres presented here reflects mainly the level of RPA recruited during S-phase and mask the amount of RPA that acts as a processivity factor for telomerase.

While the peak of RPA binding occurs slightly earlier than Trt1, Tpz1, and Ccq1, one can detect increases in binding for those factors upon the arrival of the replication fork and the increase in RPA. This overlap was more clearly seen in our follow-up PLoS Genetics paper (Chang et al.), where telomerase recruitment was monitored by a ChIP assay targeting telomere repeats (by measuring hybridization signals to a telomere repeat probe), rather than by a qPCR-based assay using sub-telomere primer pairs.

In any case, RPA is also bound to telomeres throughout the cell cycle. Its binding is significantly increased in the late S phase as the replication fork moves into telomeres. It is not clear to us why RPA showing an increase in binding during S-phase poses a problem for our proposal that RPA has an additional function in promoting telomerase activation.

We are not disputing that PRA performs multiple functions at telomeres that contribute to telomere maintenance. The main point of the paper here is that RPA has a unique and separate additional function, specifically in the telomerase activation step, that is distinct from its roles during replication or DNA damage responses, and this function likely reflects direct and extensive protein-protein interactions that RPA makes with both Trt1 and Tpz1.

**Reviewer #3:** In this manuscript, Moser and colleagues investigate the role of RPA in telomerase activity. Previous works in different model organisms suggested that RPA may regulate telomerase access and activity at telomeres, but the underlying molecular mechanisms remain to be investigated more closely, particularly in Schizosaccharomyces pombe.

Using extensive genetic analyses and a structural modeling approach, the authors dissect the regulation of telomerase recruitment and activity by RPA and Tpz1. More specifically, they analyzed many separation-of-function mutants in RPA, Tpz1 and Trt1 for their ability to recruit telomerase or to maintain telomeres, and used AphaFold3 modeling to interpret the role of point mutants and guide the analyses of interaction interfaces. They propose a ternary RPA-Trt1-Tpz1 complex held together with multiple interfaces between RPA-Trt1, RP1-Tpz1, and Trt1-Tpz1 (through the TEL-patch). For example, they characterize the ssb1-W523C mutant capable of telomerase recruitment but not activity, since telomeres are not stably maintained, suggesting that RPA is important for telomerase activity. Finally, the authors provide some pieces of evidence in Saccharomyces cerevisiae and with human RPA, as well as structural modeling insights, to suggest the conservation of the regulation of telomerase by RPA.

Overall, this work is excellent and the investigation very extensive and detailed, based on rigorous genetic analyses and convincing structural modeling interpretation. I have only a few minor comments, listed below:

We thank Reviewer #3 for an overall very positive assessment of our current work.

Line 77: It is not clear that the names of RPA subunits inside or outside the parentheses correspond to the human complex. Clarifying the names of the RPA subunits in the different mentioned organisms as early as possible would help.

They are all human subunit names. (They represent alternative names used for the same human RPA subunits.)

To clarify this point, we have modified this section of the paper to refer to these subunits as RPA1/RPA70, RPA2/RPA32, and RPA3/RPA14 (line 82 in the revised manuscript).

Line 142, “Surprisingly, D235K led to a slight increase in telomere length”: This is not obvious on the image, unless there is a quantitative analysis to support this result.

As already detailed in response to Reviewer #1, we decided to simplify and step back in our interpretation of this mutant, and it now simply notes that this mutant did not cause telomere shortening.

Line 159, “Chromatin immunoprecipitation (ChIP) assays for Trt1”: Although the primers are mentioned in the method section, it could be useful to explain in the text which telomere/subtelomere is targeted by qPCR.

We have modified Fig S1A to indicate the annealing sites of the primers used in the ChIP assays.

Lines 169-170, “Multiple double mutant clones either lost telomeres completely or displayed subtelomeric rearrangements and telomere amplification”: How many generations or passages were needed to observe these rearrangements? Is this consistent with alternative maintenance pathways? Can the authors comment on the variability of the rearrangements?

We selected two independent double-mutant strains after a meiotic cross and spore dissection for *L222A* and *Y264H* in combination with *tpz1-K75A*. They showed consistent results (after 5x restreaks), and we did not characterize these mutants further (Fig S2F).

For *tpz1-K75A ssb1-C482S*, 4 independent clones were chosen for restreaks. At 5x restreaks, 3 out of 4 completely lost telomeres, while the remaining clone showed very faint telomeres. This clone also completely lost telomeres by 10x restreak. In Fig S2G, we show 3 clones after 5 restreaks that have lost telomeres. Since all 4 clones lost telomeres, telomere loss in this strain appears to be predominant and consistent across this double mutant combination.

For *tpz1-K75A ssb1-L355P*, we initially restreaked 2 independent clones. After 5x restreaks, one clone lost telomeres while another clone showed short telomeres with an additional prominent ~1.5 kb band, likely representing amplified sub-telomeric repeats that also hybridize to telomeric repeats. Upon further restreaks of this strain to 10x, the telomere-binding patterns did not change, suggesting that this strain has found a way to stably maintain short telomeres with amplified subtelomeres. Because of inconsistent telomere phenotypes, we returned to 4 independent clones of early-generation stocks for *tpz1-K75A ssb1-L355P* and performed additional restreaks. After 5x restreaks, one clone showed a faint telomere signal, another clone showed a short telomere band along with an amplified ~1.5 kb band, and the remaining 2 clones completely lost telomeres. After 10x restreaks for those 4 clones, the telomere signal and banding pattern did not change compared to 5x restreak strains. Thus, in Fig 2H, we decided to show 10x restreaks (repeat) for the 4 independent clones of *tpz1-K75A ssb1-L355P* with mixed phenotypes.

For *tpz1-K75A ssb1-Y474N*, we also initially restreaked 2 independent clones. After 5 restreaks, one clone showed a very faint, rearranged telomere, while another showed a very strong telomere signal with highly elongated telomeres. The clone with highly elongated telomeres was restreaked 10x and 15x, and both generations showed telomeres that were elongated and heterogeneous, suggesting stable maintenance of highly elongated telomeres in this clone. Because of inconsistent results for the initial 2 clones tested, we then repeated restreaks for 2 more clones (for a total of 4 independently derived clones). After 5x restreaks, one clone completely lost telomeres, while the other showed highly heterogeneous telomeres with prominent bands at 1.5 and ~4 kb, which did not change further after 10x restreaks. For this clone, we have chosen to show 5x restreaks for these 4 independently restreaked strains, which reflect the mixture phenotype we observed for this double mutant combination.

Fig. 2, especially E and F: The Southern blots could be better aligned vertically to the ChIP graphs for samples a to f, which are the most important.

We considered changing the alignment of Fig 2E and 2F, but this would make the figure wider, which would then require it to be scaled down to fit a page in the journal. Since this is a big figure as it is, we decided against altering this figure.

Lines 227-229: Could the authors provide metrics on the quality and confidence of the models (pLDDT for example).

We generated a new supplemental figure (Fig S14) that shows the AlphaFold3 model, color-coded by pLDDT values. While this figure shows a single representative model for each of the fission yeast, budding yeast, and mammalian models, additional models generated by the AlphaFold server showed a comparable degree of confidence, as indicated by pLDDT values. In any case, we now provide the full original output files of the AlphaFold3 models generated by AlphaFold3 server as supplemental S3 data for all our models, if anyone is interested in analyzing them in more detail.

Lines 261-265: Do the authors envision the presence of RPA on the 3’ overhang at all phases of the cell cycle or only when the single-stranded DNA is long enough? Or do they think that RPA travels with the replication fork and interacts with Trt1 at the end of the S phase? The sources of single-stranded DNA are discussed in the Discussion section, but it might be useful to have a pointer to the discussion here to help the reader picturing the whole process.

We detected RPA binding at telomeres throughout the cell cycle in our previous cell-cycle ChIP assays (Moser et al., EMBO J. 2009). On the other hand, a significant increase in RPA binding was observed as the replication fork reaches telomeres (based on MCM and Pol epsilon binding).

Lines 306-307, “In contrast, ssb2-W112A caused reduced telomere binding despite normal Ssb2 protein expression”: Ssb2-W112A appears to be more expressed than the wt.

We do agree that the *ssb2-W112A* mutant cells seem to show increased expression compared to wt cells. Thus, we have modified our sentence as: *ssb2-W112A* caused reduced telomere binding despite “robust” Ssb2 protein expression. (Quantification of this particular gel found a ~4-fold increase in Ssb2 when normalized to tubulin expression.)

However, the important point here is that none of the *ssb2* mutants showed reduced Ssb2 protein expression, and a reduction in protein levels cannot account for the reduced accumulation of Ssb2 at short telomeres in the *ssb2-W112A* mutant compared to wt or other *ssb2* mutants.

Lines 416-418: Could the authors speculate on the mechanisms by which RPA-Trt1, RPA-Tpz1 and Trt1-Tpz1 interactions inhibit recombination? Do they lock RPA on the single-stranded DNA, thus preventing Rad51 loading?

At this point, we cannot be sure exactly what is happening, but that is certainly one possibility. Since we are unsure of the exact mechanism, we would rather not speculate too much on this and other possible mechanisms at this time.

Fig. 6B: Just based on these Southern blots, it is not clear whether the additional bands seen in the right-hand panel correspond to subtelomeric rearrangements as the authors state. They could correspond to telomere elongation, possibly telomerase-dependent or independent. Besides, it would be interesting to show the bands corresponding to Y’ elements, the intensity of which might reflect changes of Y’ element copy number.

We thank Reviewer #3 for his/her insights and suggestions, but we feel that further analysis should be left to future investigations.

Discussion: I found the discussion somewhat lengthy and at times redundant with the results section.

We apologize for the lengthy discussion, but we felt it was necessary to remind readers of the critical data most relevant to each discussion point, given the large amount of data presented in this manuscript.
