# Supplementary material for: Fission yeast RPA–TERT–Tpz1TPP1 complex promotes telomere extension and suppresses telomere recombination
Source: PLoS Genet. 2026 Sep 8;22(9):e1012297. doi: 10.1371/journal.pgen.1012297 (PMC13577582; doi:10.1371/journal.pgen.1012297)
Supplement: Peer review history

# Author comment: Author response to Decision Letter 0

**DOI:** 10.1371/journal.pgen.1012297.r001

**Transfer Alert**

This paper was transferred from another journal. As a result, its full editorial history (including decision letters, peer reviews and author responses) may not be present.

13 Apr 2026

# Peer review: Decision Letter 0

**DOI:** 10.1371/journal.pgen.1012297.r002

**Authors:** Geraldine Butler, Soni Lacefield

28 May 2026

PGENETICS-D-26-00361

Fission yeast RPA–TERT–Tpz1TPP1 complex promotes telomere extension and suppresses telomere recombination

PLOS Genetics

Dear Dr. Nakamura,

Thank you for submitting your manuscript to PLOS Genetics. After careful consideration, we feel that it has merit but does not fully meet PLOS Genetics's publication criteria as it currently stands. Therefore, we invite you to submit a revised version of the manuscript that addresses the points raised during the review process.

Please submit your revised manuscript by Jul 27 2026 11:59PM. If you will need more time than this to complete your revisions, please reply to this message or contact the journal office at plosgenetics@plos.org. When you're ready to submit your revision, log on to https://www.editorialmanager.com/pgenetics/ and select the 'Submissions Needing Revision' folder to locate your manuscript file.

Please include the following items when submitting your revised manuscript:

* A letter that responds to each point raised by the editor and reviewer(s). You should upload this letter as a separate file labeled 'Response to Reviewers'. This file does not need to include responses to any formatting updates and technical items listed in the 'Journal Requirements' section below.

* A marked-up copy of your manuscript that highlights changes made to the original version. You should upload this as a separate file labeled 'Revised Manuscript with Track Changes'.

* An unmarked version of your revised paper without tracked changes. You should upload this as a separate file labeled 'Manuscript'.

If you would like to make changes to your financial disclosure, competing interests statement, or data availability statement, please make these updates within the submission form at the time of resubmission. Guidelines for resubmitting your figure files are available below the reviewer comments at the end of this letter.

As the corresponding author, your ORCID iD is verified in the submission system and will appear in the published article. PLOS supports the use of ORCID, and we encourage all coauthors to register for an ORCID iD and use it as well. Please encourage your coauthors to verify their ORCID iD within the submission system before final acceptance, as unverified ORCID iDs will not appear in the published article. Only the individual author can complete the verification step; PLOS staff cannot verify ORCID iDs on behalf of authors.

We look forward to receiving your revised manuscript.

Kind regards,

Soni Lacefield, PhD

Academic Editor

PLOS Genetics

Geraldine Butler

Section Editor

PLOS Genetics

Aimée Dudley

Editor-in-Chief

PLOS Genetics

Anne Goriely

Editor-in-Chief

PLOS Genetics

**Additional Editor Comments:**

We thank you for your submission. Please be sure to respond to the reviewer's concerns and pay close attention to the requested statistical analyses.

**Journal Requirements:**

If the reviewer comments include a recommendation to cite specific previously published works, please review and evaluate these publications to determine whether they are relevant and should be cited. There is no requirement to cite these works unless the editor has indicated otherwise.

1) We do not publish any copyright or trademark symbols that usually accompany proprietary names, eg ©,  ®, or TM  (e.g. next to drug or reagent names). Therefore please remove all instances of trademark/copyright symbols throughout the text, including:

- ® on page: 32.

2) We have noticed that you have uploaded Supporting Information files, but you have not included a complete list of legends. Please add a full list of legends for your Supporting Information files after the references list.

**Reviewers' comments:**

Reviewer's Responses to Questions

**Comments to the Authors:**

**Please note here if the review is uploaded as an attachment.**

Reviewer #1: In the manuscript, Fission yeast RPA–TERT–Tpz1TPP1 complex promotes telomere extension and suppresses telomere recombination, the authors examine the role of the single-stranded DNA binding protein RPA in telomerase activation at fission yeast telomeres. While a role for RPA has been demonstrated in budding yeast and human cells, this manuscript is the first to characterize these interactions in fission yeast. The authors take advantage of AlphaFold 3 modeling combined with related experimental structures and existing genetic characterization across multiple species to propose interactions between the catalytic subunit of telomerase, the TPP1/Tpz1/Est3 protein, and the largest and second largest subunits of RPA that appear to facilitate telomerase action at telomeres. The authors present an analysis of an impressive number of mutants with characterization of telomere length, protein association with telomeres by ChIP, and expression levels. In some cases, reciprocal suppression and epistasis experiments support the proposed model. The work expands our understanding of interactions that regulate telomerase activity at telomeres and the analysis of similarities and differences between human telomerase and major model systems is useful and informative.

While the experiments are extensive and the results are presented clearly, in some cases it is difficult to ascertain reproducibility and no statistical tests are presented to support claims. Additional quantification and attention to statistical significance is warranted. This is particularly true for the ChIP experiments since the analysis of Trt1 association with telomeric DNA underpins a major conclusion of the paper that the interactions analyzed primarily affect telomerase action and not recruitment.

Major comments:

1. In cases where telomere length is measured, the number of times independent cultures were analyzed should be indicated (at least in cases where only a single sample is shown). Ideally, telomere length should be quantified to demonstrate reproducibility and the extent of the effect, particularly when the changes are subtle.

2. 142-144: Authors state, “Surprisingly, D235K led to a slight increase in telomere length.” Figure S1E. If there is an increase, it is very hard to discern given that the markers didn’t run the same amount on the two sides of the gel and these samples are in the middle (and the two colonies don’t look the same). This example illustrates why telomere lengths should be quantified throughout the manuscript.

3. The authors should state more clearly what the n values are for the ChIP experiments and what each point on the graph represents. It looks to me like there are often different numbers of points for different restreaks (Figure 2D-G). Why is that? I assume that the Southern blot is taken from one repeat? That should be stated.

4. ChIP assays require statistical analysis to support the statements made, with appropriate adjustment for multiple comparisons.

5. Why are the protein expression levels not quantified in S2B? Even with only 2 samples, it would be useful to have the quantification given.

Minor comments:

1. In experiments like those shown in Figure 2D, it would be helpful to be clearer that the mutant trt1 protein is the Myc-tagged version that is being immunoprecipitated. As shown, the labeling seems to indicate that WT trt1-myc is being expressed in these cells.

2. In Figure 5, it would be very helpful to color code the different interactions to help the reader keep track.

3. 6E needs to be done additional times and statistical analysis done before claiming there is a difference.

4. Figure 6J: spiens rather than sapiens

Reviewer #2: Recent studies have demonstrated that RPA activates telomerase in humans and budding yeast by interacting with TERT and TPP1 orthologs, indicating a direct role of RPA in its activation. In this study, Moser and colleagues used genetic and structural modelling to demonstrate the formation of a ternary RPA–Trt1–Tpz1 complex in fission yeast. This is interesting work that brings new insights into the mechanism underlying RPA-driven telomerase activation in the light of previous studies. However, some conclusions are sometimes reached without accounting for all possible hypotheses (e.g. for W523C mutant). A large number of mutants have been tested, which makes the reading difficult to follow. I would recommend to remove some data to lighten the study and to focus on the most interesting results that support the ternary RPA–Trt1–Tpz1 complex. I raised several points that should be addressed before publication.

-Mutants L222A, D235K and T265A that are equivalent to mutation associated with telomeropathies in human have been tested in fission yeast. D235K led to a slight increase of telomere length (TL). Their interpretation is that gain of function of D235K increase RPA-Rad3 association. In contrast, the L222A which is also a gain of function mutant shows a modest shortening. Since the affinity for ssDNA have not been tested for L222A, D235K and T265A mutants, I will be cautious with the Rad3 interpretation that is contradicted by the L222A mutant. Moreover, the genetic analysis of these mutants adds little to the article’s main focus and can safely be omitted if not developed further.

-“Chromatin immunoprecipitation (ChIP) assays for Trt1 detected moderate increases in telomere binding for all mutants except Y474N, which showed slightly reduced binding (Figs 1D and S2D). Thus, telomere shortening in these strains is unlikely to result from reduced telomerase recruitment and instead reflects impaired telomerase-mediated extension.”

In figure 1D, telomerase recruitment has been tested in rpa1 mutants with short TL. I agree that telomerase seems to be recruited at a higher level than WT (although we miss statistical analysis here), however one possible explanation for this increase would be that telomeric replication stress provoked by Rpa1 mutations causes telomerase recruitment, since it has been shown that telomerase can be recruited at stalled or broken replication forks.

This is particularly true for the W523C mutation for which the authors conclude that “Taken together, these results identify ssb1-W523C as a separation-of-function mutant that uncouples telomerase recruitment from telomerase activation.” According to the Alphafold model, this residue contacts ssDNA and previous published results from the Nakamura lab showed that this mutant strain is sensitive to replication stress (HU and MMS), indicating that this substitution highly likely affects ssDNA properties of RPA. (ssDNA binding assays and DNA 2D-gel electrophoresis might eventually be done to demonstrate the neutrality of this mutation). Thus, it seems to me that this mutant it clearly not a separation-of-function mutant. On the top of that, the equivalent mutation of W523C (RPA1-W528C in human) has a modest effect on telomerase processivity (Fig 6E). This mutation is somehow reminiscent to D223Y mutation that impairs ssDNA binding properties of RPA, cause sensitivity to DNA damaging agents and provokes the accumulation of RPA at telomeres.

It is likely that the amount of RPA detected by ChIP at telomeres in this study reflects mainly the level of RPA recruited during replication at forks and not the level of RPA that act as telomerase-processivity factor, leading to a misinterpretation of the results.

In addition, “A similar pattern was observed in the catalytically inactive trt1-D743A mutant (Fig 2E), indicating that ssb1-W523C cells are not defective in telomerase recruitment, but instead fail to engage in productive elongation of telomeres.” I don’t understand this conclusion. Comparing Fig 2D and 2E, it seems clear that Trt1-D734A exacerbates the shortening of telomeres, clearly indicating that W523C mutant shortens its telomeres not only because of a defect of telomerase activation

In conclusion, I found the analysis of W523C mutant uncomplete and misinterpreted, burdening this work. I would recommend to focus the work on residues that alter Rpa1-Trt1 and Rpa2-Tr1t interactions.

-line 253: tpz1-E74 instead of tpz1-K74?

-Again, I advise caution in interpreting the Rpa2-W112A data, since this W112 contacts ssDNA. Overall, It is intriguing that ssDNA contact residues are also involved in Trt1 interaction. Does it reveal a change in the mode of binding of RPA to ssDNA to accommodate telomerase association.

-Direct telomerase assay in Fig 6E showed that the equivalent mutation of W523C (RPA1-W528C) has a modest effect on telomerase processivity. Thus, it seems contradictory with the separation-of-function W523C mutant.

Does the equivalent mutation of Rpa2-W112A has been tested by telomerase assay?

-Budding yeast data doesn’t seem to bring more insights. This can be removed to lighten the work.

- One aspect that is not discussed here is that RPA is mainly recruited at telomeres in S-phase than much earlier telomerase (shown by the Nakamura lab EMBO 2009). How does this fit with the model presented here?

Again, a main concern here is that asynchronous ChIP of RPA at telomeres presented here reflects mainly the level of RPA recruited during S-phase and mask the amount of RPA that acts as a processivity factor for telomerase.

Reviewer #3: In this manuscript, Moser and colleagues investigate the role of RPA in telomerase activity. Previous works in different model organisms suggested that RPA may regulate telomerase access and activity at telomeres, but the underlying molecular mechanisms remain to be investigated more closely, particularly in Schizosaccharomyces pombe.

Using extensive genetic analyses and a structural modeling approach, the authors dissect the regulation of telomerase recruitment and activity by RPA and Tpz1. More specifically, they analyzed many separation-of-function mutants in RPA, Tpz1 and Trt1 for their ability to recruit telomerase or to maintain telomeres, and used AphaFold3 modeling to interpret the role of point mutants and guide the analyses of interaction interfaces. They propose a ternary RPA-Trt1-Tpz1 complex held together with multiple interfaces between RPA-Trt1, RP1-Tpz1, and Trt1-Tpz1 (through the TEL-patch). For example, they characterize the ssb1-W523C mutant capable of telomerase recruitment but not activity, since telomeres are not stably maintained, suggesting that RPA is important for telomerase activity. Finally, the authors provide some pieces of evidence in Saccharomyces cerevisiae and with human RPA, as well as structural modeling insights, to suggest the conservation of the regulation of telomerase by RPA.

Overall, this work is excellent and the investigation very extensive and detailed, based on rigorous genetic analyses and convincing structural modeling interpretation. I have only a few minor comments, listed below:

Line 77: It is not clear that the names of RPA subunits inside or outside the parentheses correspond to the human complex. Clarifying the names of the RPA subunits in the different mentioned organisms as early as possible would help.

Line 142, “Surprisingly, D235K led to a slight increase in telomere length”: This is not obvious on the image, unless there is a quantitative analysis to support this result.

Line 159, “Chromatin immunoprecipitation (ChIP) assays for Trt1”: Although the primers are mentioned in the method section, it could be useful to explain in the text which telomere/subtelomere is targeted by qPCR.

Lines 169-170, “Multiple double mutant clones either lost telomeres completely or displayed subtelomeric rearrangements and telomere amplification”: How many generations or passages were needed to observe these rearrangements? Is this consistent with alternative maintenance pathways? Can the authors comment on the variability of the rearrangements?

Fig. 2, especially E and F: The Southern blots could be better aligned vertically to the ChIP graphs for samples a to f, which are the most important.

Lines 227-229: Could the authors provide metrics on the quality and confidence of the models (pLDDT for example).

Lines 261-265: Do the authors envision the presence of RPA on the 3’ overhang at all phases of the cell cycle or only when the single-stranded DNA is long enough? Or do they think that RPA travels with the replication fork and interacts with Trt1 at the end of the S phase? The sources of single-stranded DNA are discussed in the Discussion section, but it might be useful to have a pointer to the discussion here to help the reader picturing the whole process.

Lines 306-307, “In contrast, ssb2-W112A caused reduced telomere binding despite normal Ssb2 protein expression”: Ssb2-W112A appears to be more expressed than the wt.

Lines 416-418: Could the authors speculate on the mechanisms by which RPA-Trt1, RPA-Tpz1 and Trt1-Tpz1 interactions inhibit recombination? Do they lock RPA on the single-stranded DNA, thus preventing Rad51 loading?

Fig. 6B: Just based on these Southern blots, it is not clear whether the additional bands seen in the right-hand panel correspond to subtelomeric rearrangements as the authors state. They could correspond to telomere elongation, possibly telomerase-dependent or independent. Besides, it would be interesting to show the bands corresponding to Y’ elements, the intensity of which might reflect changes of Y’ element copy number.

Discussion: I found the discussion somewhat lengthy and at times redundant with the results section.

**********

**Have all data underlying the figures and results presented in the manuscript been provided?**

Large-scale datasets should be made available via a public repository as described in the *PLOS Genetics*  data availability policy, and numerical data that underlies graphs or summary statistics should be provided in spreadsheet form as supporting information.

Reviewer #1: **No:** I do not see data corresponding to the ChIP graphs in the supplement.

Reviewer #2: Yes

Reviewer #3: Yes

**********

PLOS authors have the option to publish the peer review history of their article (what does this mean?). If published, this will include your full peer review and any attached files.

If you choose “no”, your identity will remain anonymous but your review may still be made public.

**Do you want your identity to be public for this peer review?**  For information about this choice, including consent withdrawal, please see our Privacy Policy.

Reviewer #1: No

Reviewer #2: No

Reviewer #3: No

**Figure resubmission:**

While revising your submission, we strongly recommend that you use PLOS’s NAAS tool (https://ngplosjournals.pagemajik.ai/artanalysis) to test your figure files. NAAS can convert your figure files to the TIFF file type and meet basic requirements (such as print size, resolution), or provide you with a report on issues that do not meet our requirements and that NAAS cannot fix.

After uploading your figures to PLOS’s NAAS tool - https://ngplosjournals.pagemajik.ai/artanalysis, NAAS will process the files provided and display the results in the "Uploaded Files" section of the page as the processing is complete. If the uploaded figures meet our requirements (or NAAS is able to fix the files to meet our requirements), the figure will be marked as "fixed" above. If NAAS is unable to fix the files, a red "failed" label will appear above. When NAAS has confirmed that the figure files meet our requirements, please download the file via the download option, and include these NAAS processed figure files when submitting your revised manuscript.

**Reproducibility:**

To enhance the reproducibility of your results, we recommend that authors of applicable studies deposit laboratory protocols in protocols.io, where a protocol can be assigned its own identifier (DOI) such that it can be cited independently in the future. Additionally, PLOS ONE offers an option to publish peer-reviewed clinical study protocols. Read more information on sharing protocols at https://plos.org/protocols?utm_medium=editorial-email&utm_source=authorletters&utm_campaign=protocols

# Author comment: Author response to Decision Letter 1

**DOI:** 10.1371/journal.pgen.1012297.r003

27 Jul 2026

Attachment Submitted filename: Response to Reviewers.docx

# Peer review: Decision Letter 1

**DOI:** 10.1371/journal.pgen.1012297.r004

**Authors:** Geraldine Butler, Soni Lacefield

19 Aug 2026

Dear Dr Nakamura,

We are pleased to inform you that your manuscript entitled "Fission yeast RPA–TERT–Tpz1TPP1 complex promotes telomere extension and suppresses telomere recombination" has been editorially accepted for publication in PLOS Genetics. Congratulations!

Before your submission can be formally accepted and sent to production you will need to complete our formatting changes, which you will receive in a follow up email. Please be aware that it may take several days for you to receive this email; during this time no action is required by you. Please note: the accept date on your published article will reflect the date of this provisional acceptance, but your manuscript will not be scheduled for publication until the required changes have been made.

Once your paper is formally accepted, an uncorrected proof of your manuscript will be published online ahead of the final version, unless you’ve already opted out via the online submission form. If, for any reason, you do not want an earlier version of your manuscript published online or are unsure if you have already indicated as such, please let the journal staff know immediately at plosgenetics@plos.org.

In the meantime, please log into Editorial Manager at https://www.editorialmanager.com/pgenetics/, click the "Update My Information" link at the top of the page, and update your user information to ensure an efficient production and billing process. Note that PLOS requires an ORCID iD for all corresponding authors. Therefore, please ensure that you have an ORCID iD and that it is validated in Editorial Manager. To do this, go to ‘Update my Information’ (in the upper left-hand corner of the main menu), and click on the Fetch/Validate link next to the ORCID field.  This will take you to the ORCID site and allow you to create a new iD or authenticate a pre-existing iD in Editorial Manager.

If you have a press-related query, or would like to know about making your underlying data available (as you will be aware, this is required for publication), please see the end of this email. If your institution or institutions have a press office, please notify them about your upcoming article at this point, to enable them to help maximise its impact. Inform journal staff as soon as possible if you are preparing a press release for your article and need a publication date.

Thank you again for supporting open-access publishing; we are looking forward to publishing your work in PLOS Genetics!

Yours sincerely,

Soni Lacefield, PhD

Academic Editor

PLOS Genetics

Geraldine Butler

Section Editor

PLOS Genetics

Aimée Dudley

Editor-in-Chief

PLOS Genetics

Anne Goriely

Editor-in-Chief

PLOS Genetics

www.plosgenetics.org

BlueSky: @plos.bsky.social

----------------------------------------------------

Comments from the reviewers (if applicable):

Congratulations on the acceptance! We would like the authors to consider adding to the text to respond to reviewer 2's comments on potential alternative models for Rpa3-GFP associated with telomere shortening and the possibility that it may not be a complete separation of function allele.

Reviewer's Responses to Questions

**Comments to the Authors:**

**Please note here if the review is uploaded as an attachment.**

Reviewer #1: The authors have addressed my concerns. I appreciate the extensive dataset provided (along with transparent statistical analysis) to support key findings in the manuscript.

Reviewer #2: I appreciate the authors' efforts in addressing the comments and implementing the requested modifications to the manuscript, including the new figures.

In Figure 1D, the authors assume that telomerase recruitment is equivalent to telomere elongation. Have they considered that telomerase might also play a protective role at telomeric DNA ends? In other words, detecting telomerase by ChIP does not necessarily indicate a failure to maintain telomere length, contrary to their conclusion.

I still have concerns about the analysis of the W523C mutant. My reasoning was based on its sensitivity to HU and MMS (Figure 3B in Xu et al., 2023, Nakamura’s lab). However, the fact that the W523C strain in this previous study has likely circular chromosomes made the interpretation confusing. According to new figure S4A, I agree that W523C is less severe than D223Y.

That said, the accumulation of Rpa3-GFP associated with telomere shortening is not definitive proof. One alternative explanation is that telomere shortening increases replication stress as demonstrated, leading to ssDNA accumulation and RPA recruitment.

I would urge extreme caution when drawing conclusions about W523C as a true separation-of-function mutant. In my view, demonstrating a separation-of-function for this mutant requires direct confirmation of the ssDNA-binding properties of the mutated complex.

Reviewer #3: The authors have addressed the few comments I had. Congratulations to them for the extensive work.

**********

**Have all data underlying the figures and results presented in the manuscript been provided?**

Large-scale datasets should be made available via a public repository as described in the *PLOS Genetics*  data availability policy, and numerical data that underlies graphs or summary statistics should be provided in spreadsheet form as supporting information.

Reviewer #1: Yes

Reviewer #2: Yes

Reviewer #3: Yes

**********

PLOS authors have the option to publish the peer review history of their article (what does this mean?). If published, this will include your full peer review and any attached files.

If you choose “no”, your identity will remain anonymous but your review may still be made public.

**Do you want your identity to be public for this peer review?**  For information about this choice, including consent withdrawal, please see our Privacy Policy.

Reviewer #1: No

Reviewer #2: No

Reviewer #3: No

----------------------------------------------------

**Data Deposition**

If you have submitted a Research Article or Front Matter that has associated data that are not suitable for deposition in a subject-specific public repository (such as GenBank or ArrayExpress), one way to make that data available is to deposit it in the Dryad Digital Repository. As you may recall, we ask all authors to agree to make data available; this is one way to achieve that. A full list of recommended repositories can be found on our website.

The following link will take you to the Dryad record for your article, so you won't have to re‐enter its bibliographic information, and can upload your files directly:

http://datadryad.org/submit?journalID=pgenetics&manu=PGENETICS-D-26-00361R1

More information about depositing data in Dryad is available at http://www.datadryad.org/depositing. If you experience any difficulties in submitting your data, please contact help@datadryad.org for support.

Additionally, please be aware that our data availability policy requires that all numerical data underlying display items are included with the submission, and you will need to provide this before we can formally accept your manuscript, if not already present.

----------------------------------------------------

**Press Queries**

If you or your institution will be preparing press materials for this manuscript, or if you need to know your paper's publication date for media purposes, please inform the journal staff as soon as possible so that your submission can be scheduled accordingly. Your manuscript will remain under a strict press embargo until the publication date and time. This means an early version of your manuscript will not be published ahead of your final version. PLOS Genetics may also choose to issue a press release for your article. If there's anything the journal should know or you'd like more information, please get in touch via plosgenetics@plos.org.

# Editor report: Acceptance letter

**DOI:** 10.1371/journal.pgen.1012297.r005

**Authors:** Geraldine Butler, Soni Lacefield

PGENETICS-D-26-00361R1

Fission yeast RPA–TERT–Tpz1TPP1 complex promotes telomere extension and suppresses telomere recombination

Dear Dr Nakamura,

We are pleased to inform you that your manuscript entitled "Fission yeast RPA–TERT–Tpz1TPP1 complex promotes telomere extension and suppresses telomere recombination" has been formally accepted for publication in PLOS Genetics! Your manuscript is now with our production department and you will be notified of the publication date in due course.

The corresponding author will soon be receiving a typeset proof for review, to ensure errors have not been introduced during production. Please review the PDF proof of your manuscript carefully, as this is the last chance to correct any errors. Please note that major changes, or those which affect the scientific understanding of the work, will likely cause delays to the publication date of your manuscript.

Soon after your final files are uploaded, unless you have opted out or your manuscript is a front-matter piece, the early version of your manuscript will be published online. The date of the early version will be your article's publication date. The final article will be published to the same URL, and all versions of the paper will be accessible to readers.

For Research Articles, you will receive an invoice from PLOS for your publication fee after your manuscript has reached the completed accept phase. If you receive an email requesting payment before acceptance or for any other service, this may be a phishing scheme. Learn how to identify phishing emails and protect your accounts at https://explore.plos.org/phishing.

Thank you again for supporting PLOS Genetics and open-access publishing. We are looking forward to publishing your work!

With kind regards,

Sharmila Kamatchi

PLOS Genetics

On behalf of:

The PLOS Genetics Team

Carlyle House, Carlyle Road, Cambridge CB4 3DN | United Kingdom

plosgenetics@plos.org | +44 (0) 1223-442823

plosgenetics.org | Twitter: @PLOSGenetics
